# *Phycocharax rasbora*, a new genus and species of Brazilian tetra (Characiformes: Characidae) from Serra do Cachimbo, rio Tapajós basin

**DOI:** 10.1371/journal.pone.0170648

**Published:** 2017-02-15

**Authors:** Willian Massaharu Ohara, Juan Marcos Mirande, Flávio Cesar Thadeo de Lima

**Affiliations:** 1 Museu de Zoologia da Universidade de São Paulo, CEP, São Paulo, São Paulo, Brazil; 2 CONICET-Fundación Miguel Lillo, Miguel Lillo, San Miguel de Tucumán, Argentina; 3 Museu de Zoologia da Universidade Estadual de Campinas “Adão José Cardoso”, Campinas, São Paulo, Brazil; Laboratoire de Biologie du Développement de Villefranche-sur-Mer, FRANCE

## Abstract

A new genus and species of characid fish is described from rio Braço Norte, a tributary of rio Teles Pires, Tapajós basin, Mato Groso, Brazil. The new taxa can be diagnosed from the remaining characids by a unique combination of characters that includes the presence of a single row of relatively compressed premaxillary teeth, large teeth with four to nine cusps on premaxillary and dentary, absence of pseudotympanum, incomplete lateral line with 7–13 pored scales, sexually-dimorphic males with distal margin of anal fin approximately straight, and presence of a nearly triangular and horizontally elongated blotch from the posterior half of the body to caudal peduncle. The most parsimonious phylogenetic hypothesis, using morphological data, recovered the new genus and species in a clade including *Paracheirodon axelrodi* and *Hyphessobrycon elachys*.

## Introduction

The Characidae is one of the richest families among bony fishes, and depending of the alternative proposals followed [1,2], the family comprise between 1,100 to over than 1,200 species [3]. Although much advance to understand the relationships within Characidae family has been accomplished, it still constitutes in an unsettled issue. A stable classification of the Characidae is still far to be established, given the great diversity and complexity of the family and the lack of comprehensive analysis combining morphological and molecular data [4]. Many members of the Characidae have low variation either in molecular data [2] as in morphological [5,6], and most deep relationships within the family are incongruent between different analysis or have low support.

During recent ichthyological field surveys in the northern of the Brazilian State Mato Grosso, was discovered from the rio Braço Norte, a tributary of the rio Teles Pires (Serra do Cachimbo, rio Tapajós basin), an attractively-colored characid tetra that proved to be not assignable to any previously known genus. The new taxon is described herein due to its unusual combination of single row of compressed premaxillary teeth, pseudotympanum absent, and sexual dimorphism with males exhibiting a straight distal margin of the anal fin, while females present the more generalized condition among characids (i.e., an anal fin with a distinct anterior lobe). In addition, the new taxon shows a remarkable color pattern with a large and elongated triangular blotch extending along middle flanks from vertical through the base terminus of dorsal fin to the caudal peduncle end. Such blotch is similar in shape and position to the one present in the Neotropical characid *Hemigrammus pulcher* Ladiges and the Asian cyprinids of the genus *Trigonostigma* Kottelat & Witte. The aim of the present study is to describe this new taxon, assigned herein to a new genus, due to both phylogenetic position and unique combination of characters.

## Materials and methods

Fishes were prior anesthetized with eugenol solution (1 ml/L), then fixed in 10% formalin, and finally preserved in 70% ethanol. The field studies did not involve endangered species. Collection permit was granted by Instituto Brasileiro do Meio Ambiente e dos Recursos Naturais Renováveis (IBAMA 2621–1). Counts and measurements follow Fink & Weitzman [7] and Menezes & Weitzman [8], except for counts of the horizontal scale rows below the lateral line, which were counted between lateral line and pelvic-fin insertion. Horizontal scale rows between the dorsal-fin origin and lateral line does not include the scale of median predorsal series situated just anterior to the first dorsal-fin ray. The distance from the pelvic-fin origin to the anal-fin origin was added to the set of measures. Counts and measurements were taken on the left side of specimens, whenever possible. Measurements are presented as proportions of standard length (SL), except for subunits of the head, which are given as proportions of head length (HL). The frequency of each count is provided in parentheses after the respective count, asterisks indicate holotype values. Counts of vertebrae, supraneurals, procurrent caudal-fin rays and gill rakers of the first arch were taken from 108 cleared and stained specimens (c&s) prepared according to Taylor & Van Dyke [9]. Vertebrae of the Weberian apparatus were counted as four elements, and the fused PU1+U1 of the caudal region as a single element. Sexual dimorphism related with the color in life was confirmed by the examination of gonads via incision of 22 selected specimens. Animal research involving fish at the Museu de Zoologia da Universidade de São Paulo is associated with the project number 226/2015, approved by the Ethics Committee on Animal Use (CEUA) of Instituto de Biologia da Universidade de São Paulo (IB-USP). The list of material examined is provided as S1 Appendix. Institutional abbreviations follow Mirande [1,10] with inclusion of INPA (Instituto Nacional de Pesquisas da Amazônia, Manaus), MUSM (Museo de Historia Natural de la Universidad Nacional Mayor de San Marcos, Lima), UFRGS (Universidade Federal do Rio Grande do Sul, Porto Alegre), MPEG (Museu Paraense Emílio Goeldi, Belém), MNRJ (Museu Nacional do Rio de Janeiro, Rio de Janeiro), and ZUEC (Museu de Zoologia da Universidade Estadual de Campinas “Adão José Cardoso”, Campinas).

Phylogenetic relationships of the new taxon among characids were assessed by parsimony using TNT software [11] following the characters of Mirande [1,10] and Mirande et al. [12,13]. Thirty-five additional species were included to the dataset to evaluate possible close relatives to the new taxon or to check its possible inclusion in some genus already known, as well as to have a most comprehensive framework within the Characidae phylogeny. The coding of the new taxon plus 212 species analyzed herein are included in S1 Table and the dataset is available online at MorphoBank [14,15]. The complete list of 387 characters analyzed herein, with comments on those added (two new) or modified (15) from Mirande et al. [12], is provided as S2 Appendix.

As in the mentioned papers (i.e. [1,10,12,13]), analysis were performed under extended implied weighting [16,17] in a broad range of values of K (concavity constant). Differing from previous contributions, the phylogenetic analysis in a broad range of values of K was herein performed only to evaluate the possible relationships and generic assignments of the new taxon, rather than to reach to some general phylogenetic hypothesis for the family. Support was calculated with Symmetric Resampling and results are expressed as GC values according to Goloboff et al. [18].

### Nomenclatural acts

The electronic edition of this article conforms to the requirements of the amended International Code of Zoological Nomenclature, and hence the new names contained herein are available under that Code from the electronic edition of this article. This published work and the nomenclatural acts it contains have been registered in ZooBank, the online registration system for the ICZN. The ZooBank LSIDs (Life Science Identifiers) can be resolved and the associated information viewed through any standard web browser by appending the LSID to the prefix “http://zoobank.org/”. The LSID for this publication is: urn:lsid:zoobank.org:pub:D0FD20E4-D2EB-4E52-9094-DDFE45EE0BE0. The electronic edition of this work was published in a journal with an ISSN, and has been archived and is available from the following digital repositories: PubMed Central, LOCKSS.

## Results

### Family characidae agassiz

#### *Phycocharax*, new genus

urn:lsid:zoobank.org:act:27E0CD39-AD63-4D08-BE0F-60670B17406F

(Figs 1–3)

**Fig 1:**
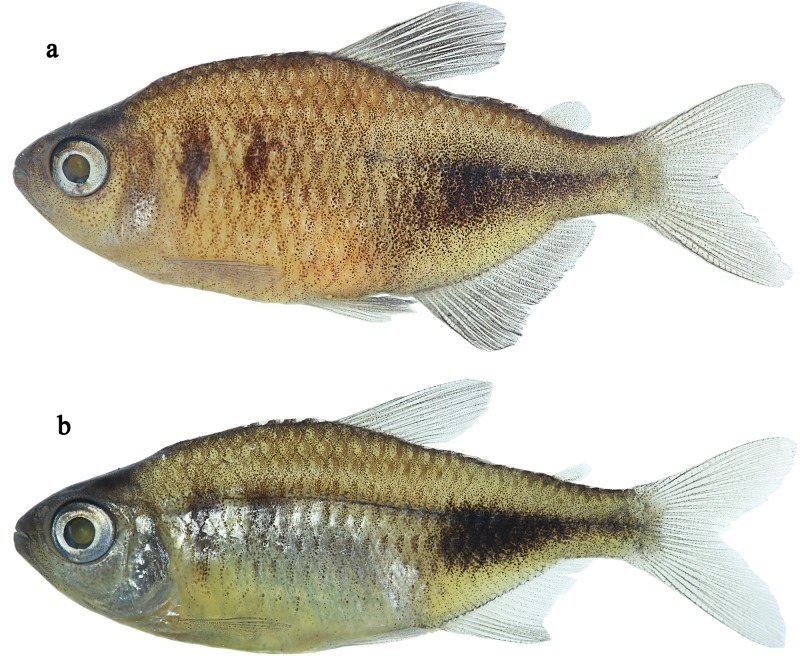
*Phycocharax rasbora*, holotype, MZUSP 119843, 29.1 mm SL, male; MZUSP 115341, 25.3 mm SL, paratype, female.

**Fig 2:**
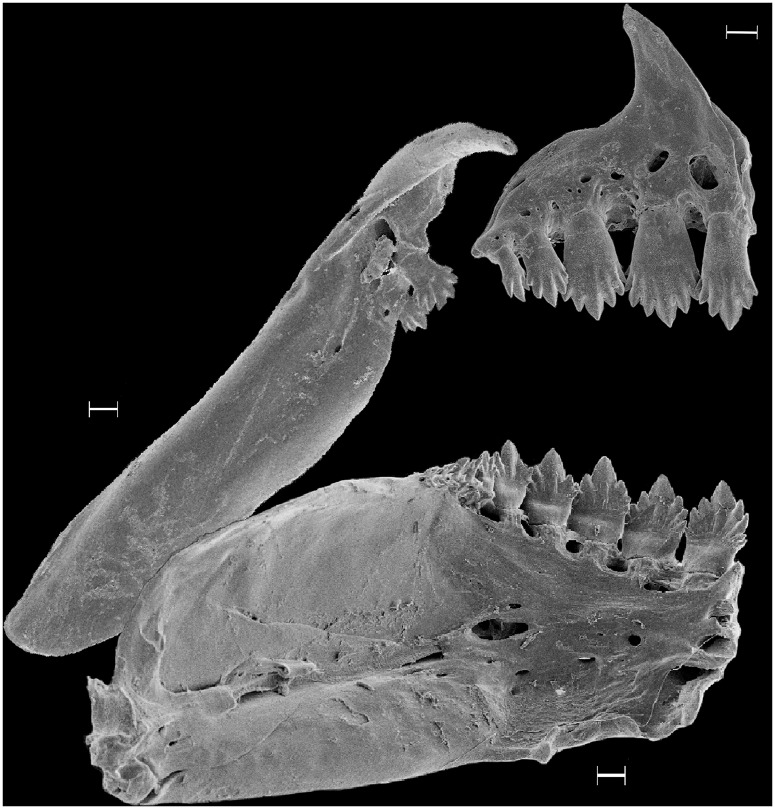
*Phycocharax rasbora*, medial view of left side; premaxilla, 29.8 mm SL; dentary and maxilla, 29.1 mm SL, paratypes, both MZUSP 115341. Scale bar: 100 μm.

**Fig 3:**
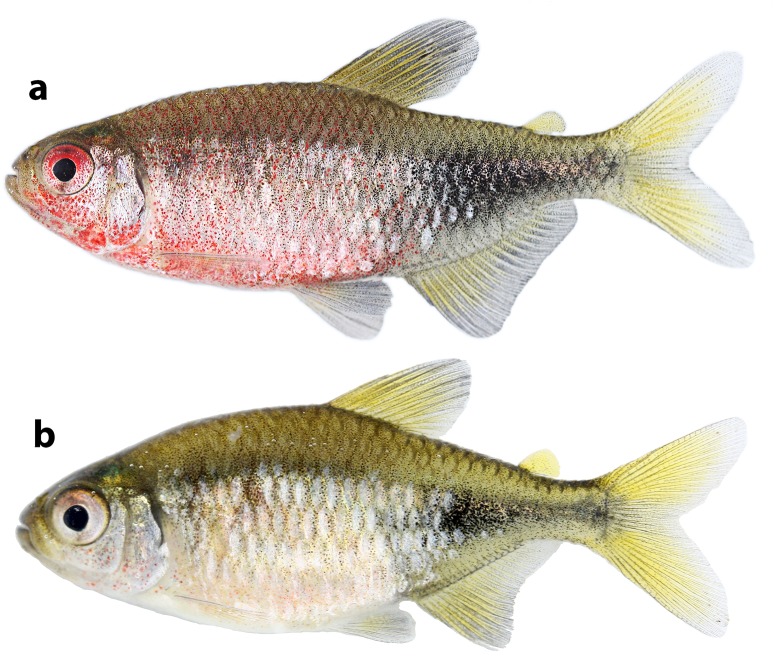
*Phycocharax rasbora*, MZUSP 119843, paratype, 29.1 male (a) and MZUSP 115313, paratype, 26.4 mm SL, female (b), immediately after collection.

**Type species:** *Phycocharax rasbora*, new species, by monotypy and original description.

**Diagnosis:** *Phycocharax* can be diagnosed from the remaining characid genera by the combination of the following character-states, none of them unique: 1) presence of single row of relatively compressed premaxillary teeth (Fig 2); 2) large teeth on premaxillary, and dentary with four to nine cusps (Fig 2); 3) pseudotympanum absent; 4) incomplete lateral line with 7–13 pored scales; 5) anal fin dimorphic, males with distal margin approximately straight, and decreasing gently posteriorly (Figs 1a and 3a), while females present slightly concave margin of anal fin with anteriormost branched rays distinctly longer than remaining rays (Figs 1b and 3b); 6) presence of horizontally-elongated somewhat triangular blotch extending from vertical through dorsal-fin terminus to caudal peduncle end (Figs 1 and 3).

**Etymology:** From the Greek *phykos*, meaning “algae”, in allusion to the feeding habit of the new taxon owing to the dominance of this resource in its stomach contents, plus *charax*, meaning “pointed stake” or “palisade of pointed sticks”, the first generic name in Characidae. Gender masculine.

#### *Phycocharax rasbora*, new species

urn:lsid:zoobank.org:act:B26FDDEB-E6C0-4BB4-9DE3-2AD71673352C

(Figs 1–3)

**Holotype:** MZUSP 119843, 29.1 mm SL, Brazil, Mato Grosso, Guarantã do Norte, Igarapé do Arnaldo, small stream of the rio Braço Norte, a tributary of rio Peixoto de Azevedo, rio Teles Pires drainage, rio Tapajós basin, 9°37’13”S 54°57’38”W, 9 Feb 2014, W. M. Ohara & J. Bilce.

**Paratypes:** All from Brazil, Mato Grosso, Guarantã do Norte at rio Braço Norte as data of holotype. INPA 52858, 25, 17.1–30.1 mm SL; MCP 49957, 20, 15.8–29.7 mm SL; MNRJ 45956, 18, 20.2–30.5 mm SL; MZUSP 115341, 115 (8 c&s), 15.0–32.1 mm SL, collected with holotype. CI-FML 7164, 4 (2 c&s), 25.8–29.0 mm SL; MZUSP 115344, 42, 17.1–30.5 mm SL; ZUEC 10440, 10 (2 c&s), 17.5‒28.6 mm SL, PCH Braço Norte IV reservoir, 9°37’51”S 54°58’38”W, 28 April 2012, J. Bilce & R. Rosa. MZUSP 115313, 3, 26.4–32.1 mm SL, immediately downstream of the PCH Braço Norte IV, 9°41’22”S 54°57’56”W, 9 Feb 2014, W. M. Ohara & J. Bilce. MZUSP 115343, 20, 27.9–33.2 mm SL; ZUEC 12775, 3, 32.3–34.1 mm SL, rio Braço Norte, Braço Norte IV reservoir, 9°37’48”S 54°58’15”W, 28 April 2012, J. Bilce et al. ANSP 200243, 10, 20.3–29.2 mm SL; CAS 241425, 10, 18.5–31.0 mm SL; CI-FML 7165, 10, 21.5–29.2 mm SL; MPEG 33928, 10, 23.4–29.0 mm SL; ZUEC 12776, 20, 14.3–30.8 mm SL; MZUSP 119405, 309, 11.6–32.1 mm SL, same locality as holotype; O. T. Oyakawa, W. M. Ohara & M. Pastana, 4 Aug 2015.

**Diagnosis:** Same as for the genus.

**Description:** Morphometric data presented in Table 1. Small sized characid, largest examined specimen with 34.1 mm SL. Body compressed, moderately short and deep (Fig 1a) to horizontally elongated (Fig 3a). Greatest body depth situated slightly anterior to vertical through dorsal-fin origin. Dorsal profile of head convex from upper lip anterior tip to vertical through anterior nostril; slightly concave or straight from that point to tip of supraoccipital spine. Dorsal profile of body convex from supraoccipital spine tip to base of last dorsal-fin ray, approximately straight or convex from that point to adipose-fin insertion and slightly concave between adipose-fin insertion and origin of anteriormost dorsal procurrent caudal-fin ray. Ventral profile of head and body convex from anterior dentary tip to anal-fin origin. Ventral profile of caudal peduncle slightly concave. Pseudotympanum absent.

**Table 1:** Morphometric data for holotype and 38 paratypes of *Phycocharax rasbora*. Values for the holotype included in range, range and mean ± SD (standard deviation).

Characters	Holotype	Range	Median±SD
Standard length (mm)	29.1	23.7–32.1	27.9±1.9
**Percentage of standard length**			
Body depth at dorsal-fin origin	39.7	33.2–40.8	36.7±2.0
Snout tip to dorsal-fin origin	53.5	49.8–56.4	53.2±1.5
Snout tip to pectoral-fin origin	28.4	27.1–32.4	28.7±1.2
Snout tip to pelvic-fin origin	51.0	47.3–56.0	51.8±1.9
Snout tip to anal-fin origin	65.2	63.7–71.1	68.7±1.8
Caudal-peduncle depth	11.3	10.5–12.9	11.5±0.5
Caudal-peduncle length	14.3	12.4–16.3	14.1±0.9
Pectoral-fin length	19.8	16.9–21.8	19.3±1.2
Pelvic-fin length	19.2	15.7–19.5	17.1±1.0
Pelvic-fin origin to anal-fin origin	17.9	15.9–20.2	18.1±1.0
Dorsal-fin base length	13.8	12.9–18.5	15.0±1.2
Dorsal-fin length	30.0	23.5–31.7	27.6±1.9
Dorsal-fin origin to caudal-fin origin	53.1	49.3–54.5	51.8±1.3
Anal-fin base length	27.2	20.5–27.4	24.8±1.8
Anal-fin length	17.9	16.7–21.8	19.0±1.1
Posterior margin of eye to dorsal-fin origin	39.9	37.4–42.1	40.0±1.1
Head length	25.7	24.2–28.6	25.6±1.0
**Percentage of head length**			
Horizontal length eye	41.0	36.7–42.7	40.0±1.4
Snout length	26.8	21.7–27.4	24.6±1.5
Least interorbital width	33.2	30.7–36.7	33.2±1.6
Upper jaw length	44.0	38.1–44.3	40.5±1.7

Jaws of equal size, mouth terminal. Posterior terminus of maxilla reaching vertical through anterior margin of pupil. Maxilla approximately at 45° angle relative to longitudinal axis of body. Anterior and posterior nostrils separated only by skin fold, posterior opening crescent-shaped. Frontals meeting each other only through the epiphyseal bar. Rhinosphenoid present with developed dorsal process. Relatively long sphenotic spine, reaching dorsal margin of hyomandibula. Frontal fontanel triangular and parietal fontanel large. Infraorbital series with five or six elements. Anterior region of third infraorbital not reaching preopercle.

Single row of premaxillary teeth, 4(9) or 5*(37) slightly compressed teeth with four to nine cusps. Symphyseal tooth narrower distally than subsequent two teeth and slightly asymmetric. Maxilla with 0(2), 1(8), 2*(33) or 3(1) compressed teeth on anterodorsal margin of bone, with three to seven cusps (Fig 2); anteriormost tooth usually largest. Maxilla suddenly expanded ventrally to maxillary teeth. Dentary with 4(2) or 5(6) slightly compressed teeth with four to nine cusps, followed by 3(2), 4(2), 5(2) or 8(2) relatively smaller conical or tricuspid teeth. Central cusp of all teeth more developed than remaining lateral cusps. Premaxillary teeth with cusp edges slightly curved outward, and dentary teeth cusp edges curved inward.

Scales cycloid, moderately large, *circuli* restricted to anterior field and five to twelve slightly divergent *radii* extending to posterior margin of scales. Incomplete lateral line slightly deflected downward with 7(6), 8*(11), 9(9), 10(4) 11(3), 12(1) or 13(2) perforated scales. Lateral series with 30(1), 31(8), 32*(15), 33(9) or 34(1) scales including pored scales. Horizontal scale rows between dorsal-fin origin and lateral line 5*(38). Horizontal scale rows between lateral line and pelvic-fin origin 3(7) or 4*(30). Scales in median series between tip of supraoccipital spine and dorsal-fin origin 6(1), 9(10) or 10*(20). Circumpeduncular scale rows 12(1), 13(4) or 14*(31). Anteriormost anal-fin rays base with single row of 2(9), 3*(18) or 4(6) scales. Caudal fin with scales only basally.

Dorsal-fin rays ii, 8(1) or 9*(42), not including small ossification anterior to first dorsal fin unbranched ray present in all examined specimens. Dorsal-fin origin slightly ahead of midbody. First dorsal-fin pterygiophore located behind neural spine of 10th(3) or 11th(5) vertebrae. Adipose fin present. Anal-fin rays iv(7) or v(1), 16(24), 17(20) or 18*(2), anteriormost rays slightly longer, subsequent rays decreasing posteriorly in size; distal margin of anal fin slightly concave in females or straight in males (see “Sexual Dimorphism”, below). Anteriormost anal-fin pterygiophore inserted posterior to haemal spine of 16th(2), 17th(5) or 18th(1) vertebrae. Pectoral-fin rays i, 9(1), 10(24), 11*(16) or 12(4). Pectoral-fin tip typically not reaching pelvic-fin insertion. Pelvic-fin rays i, 7*(46). Pelvic-fin tip almost reaching anal-fin insertion. Caudal fin with i, 9*(39) rays on the upper and 8*, i (39) rays on lower lobe. Caudal fin forked with lobes similar in size, and rounded tips. Ten (5) or 11 (3) dorsal procurrent caudal-fin rays, and 7(1), 8(3) or 9(3) ventral procurrent caudal-fin rays.

Total vertebrae 32(4), 33(3) or 34(1). Precaudal vertebrae 16(6) or 17(2); caudal vertebrae 16(4) or 17(4). Supraneurals 4(1) or 5(7) with narrow bony lamellae on upper portion. Branchiostegal rays 4(8). First gill arch with 1(1), 2(6) or 3(1) gill rakers on hypobranchial, 8(4) or 9(4) on ceratobranchial, 1(8) on cartilage between ceratobranchial and epibranchial, and 6(7) or 7(1) on epibranchial. One row of gill rakers on first ceratobranchial; two rows on remaining ones.

**Color in alcohol:** Overall body color beige to light brown, darker dorsally (Fig 1). Snout, top of head, anterior portion of maxilla, and dentary tip dark; numerous small dark chromatophores scattered on infraorbital series, opercle, and gular area, mainly in males (females lack dark chromatophores on gular area and infraorbitals). Scales on upper portion of body presenting conspicuous reticulated pattern formed by dark pigmentation concentrated along scale borders, gradually fading to lower flanks, typically more intense in males (Fig 1a) than in females (Fig 1b). Two vertically elongated and narrow humeral blotches, the first lying at level of third and fourth pored lateral-line scales, extending vertically over two or three vertical scales rows above lateral line and over one or two below it. Second blotch lying at level of sixth and seventh pored lateral line, extending vertically over two scales rows above lateral line. Humeral blotches usually conspicuous, excepting in specimens smaller than 20.8 mm SL.

Posterior region of flanks with conspicuous horizontally-elongated blotch, approximately triangular, of variable size, and extending posteriorly from vertical through dorsal-fin terminus to caudal peduncle end; its intensity ranging from blurred to sharply defined. One series of 9–20 longitudinal, anteriorly directed V-shaped marks of variable intensity along horizontal septum, more discernible in alcohol-preserved specimens. Dorsal and anal fins with numerous large dark chromatophores concentrated along interradial membranes, giving overall dark coloration to these fins, more intense in males (Fig 1a) than in females (Fig 1b). Pelvic, caudal, and adipose fins also with dark chromatophores, but in little amount in females. Pectoral fin hyaline in both sexes.

**Color in life:** Males noticeably more colored than females (Fig 3). Dorsal portion of body light beige. Humeral blotches and large dark triangular blotch located on posterior portion of the body conspicuous. Upper portion of eye red to shiny red in males (Fig 3a) and pale red in females (Fig 3b). Dorsal, adipose, caudal, and anal fins pale yellow with interradial membranes more intensely pigmented by dark chromatophores in males than females. Pectoral and pelvic fins hyaline. Head and anterior portion of body of males exhibiting red pigmentation irregularly scattered (Fig 3a). Red pigmentation in males situated anterior to vertical through anal-fin origin and more concentrated in anteroventral portion of body and head. Head and body of females predominantly silver yellowish.

**Sexual dimorphism:** Males notably more brightly colored than females (Fig 3: see “Color in life”) with conspicuous concentration of dark chromatophores on body and fins (Fig 1: see “Color in alcohol”). In addition, males show anal-fin base slightly convex and distal margin approximately straight with rays decreasing posteriorly in size, whereas females present anal-fin base straight and distal margin clearly concave, with anteriormost rays distinctly longer than remaining rays, forming a discrete anterior lobe (Fig 3). Profile along anal-fin base approximately straight (in females) or slightly convex (in males). Males apparently reach larger size than females (largest specimens examined, male 34.1 mm SL vs. female 31.4 mm SL). Secondary sexual characters related to bony-fin hooks on fins were not found and are certainly absent given the sexual maturity of many examined specimens (see “Ecological notes”). Gill filaments lack fusion.

**Fig 4:**
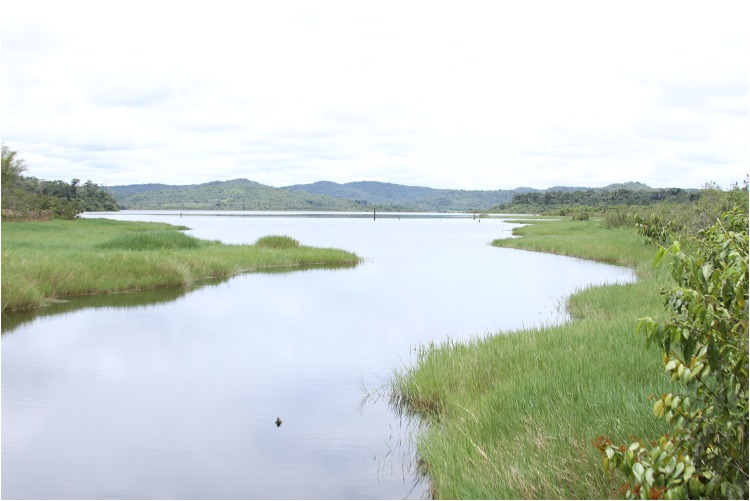
Igarapé do Arnaldo, a tributary of rio Braço Norte, near Braço Norte hydroelectric dam, Guarantã do Norte, Mato Grosso, Brazil, type-locality of *Phycocharax rasbora*.

**Ecological notes:** *Phycocharax rasbora* was collected primarily in dammed portions of the rio Braço Norte. Contrasting from other tributaries of the rio Tapajós basin, which are primarily clearwaters rivers, the rio Braço Norte is a blackwater tributary of the rio Teles Pires. In the site of occurrence of *P*. *rasbora* was build a small hydroelectric dam (PCH Braço Norte IV), and apparently the reservoir condition promoted the proliferation of algae. As consequence of the damming, the species thrived and can be considered as highly abundant at the dammed portion of the rio Braço Norte. In addition, *P*. *rasbora* seems to be uncommon in the lotic stretch of rio Braço Norte downstream of the dam, where only three specimens were collected. The type locality of the *P*. *rasbora*, the igarapé do Arnaldo, is a small tributary which was flooded during the damming of river, presenting muddy substrate with a great amount of decomposed organic matter with undergrowth marginal vegetation formed by grasses (Fig 4). In this area, *Phycocharax rasbora* was the most abundant species with 77% of all specimens collected. The section of the rio Braço Norte downstream the dam present sandy bottom with pebbles and rocks. *Phycocharax rasbora* occurs syntopically at Braço Norte reservoir with other Characiformes species, such as *Cyphocharax* cf. *spilurus* (Curimatidae), *Leporinus friderici* (Anostomidae), *Serrapinus* aff. *micropterus*, *Astyanax* aff. *bimaculatus* (Characidae), *Hoplerythrinus unitaeniatus* (Erythrinidae), Gymnotiformes, as *Gymnotus diamantinensis* (Gymnotidae), and Cichliformes, i.e., *Aequidens* sp. and *Apistogramma* sp. (Cichlidae). Although little abundant downstream the dam, *P*. *rasbora* was found co-occurring with the following Characiformes: *Leporinus desmotes* (Anostomidae), *Hemiodus quadrimaculatus* (Hemiodontidae), *Astyanax* aff. *bimaculatus*, *Bryconops* sp., *Jupiaba polylepis*, *Jupiaba paranatinga*, *Moenkhausia hasemani*, *Serrapinnus* aff. *micropterus* (Characidae), and with a single species of Siluriformes, *Hypostomus* sp. (Loricariidae).

*Phycocharax rasbora* was collected during the rainy seasons (April 2012; February 2014) and a dry season (August 2015). Males and females presented gonads well developed in both seasons. Oocytes with different sizes are present in the gonads, suggesting that females present multiple spawning, as well as several other small characids (e.g. [19,20]). Stomach contents of ten examined specimens contained mainly algae and vegetal matter.

**Fig 5:**
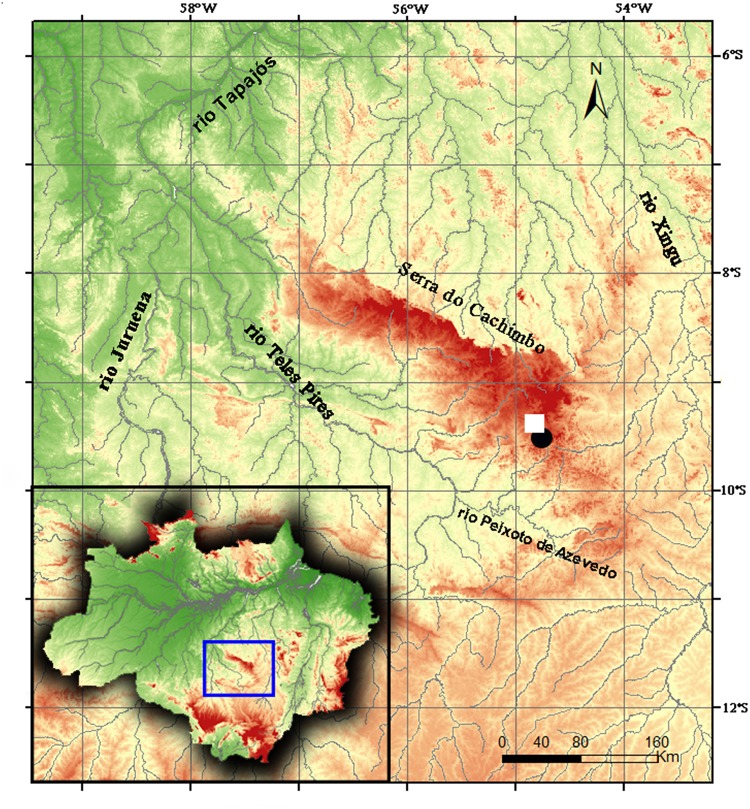
Map of Tapajós and Xingu Rivers and adjoining areas, indicating distribution and type-locality (square) of *Phycocharax rasbora*.

**Distribution:** *Phycocharax rasbora* is so far known only from its type locality at upper rio Braço Norte, a right-bank tributary of the rio Peixoto de Azevedo, part of rio Teles Pires drainage, rio Tapajós basin (Fig 5). The rio Braço Norte drains the Serra do Cachimbo at northern Mato Grosso State, Brazilian Amazon.

**Etymology:** From the Bengali word “rasbora”, the common name of the fish *Rasbora rasbora* (Hamilton). Rasbora is a generic name encompassed a great radiation of small cyprinids from southeastern Asia, including the species currently allocated in the genus *Trigonostigma* [21], which possess a dark triangular blotch on body sides very reminiscent in shape and position similarly as found in the new species. Gender masculine. A noun in apposition.

**Fig 6:**
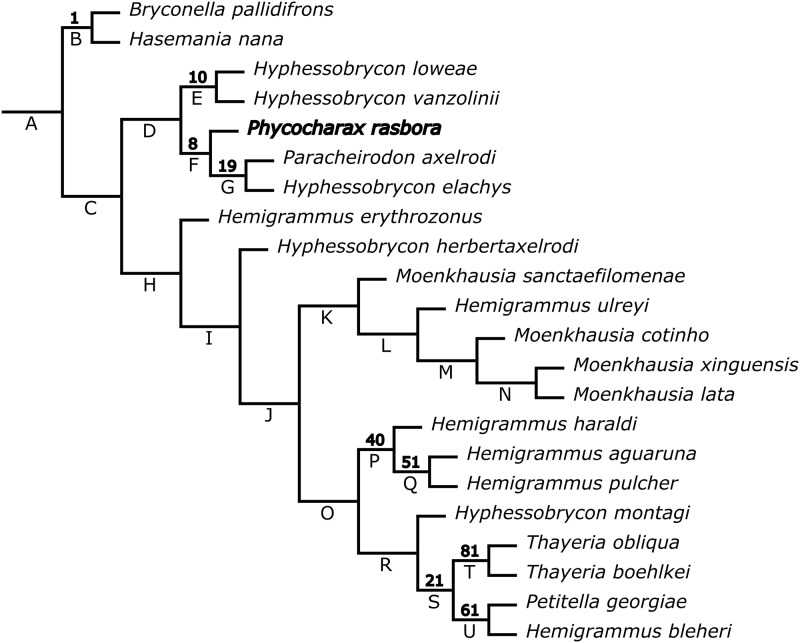
Phylogenetic relationships of *Phycocharax rasbora* under implied weighting (K = 7.5–11.4). Numbers above branches denote GC values; negative values are not shown. Synapomorphies. A: 243(0); B: 68(1), 180(1), 253(0), 307(0); C: 285(0); D: 158(1), 224(0), 246(0); E: 29(1), 65(0), 138(1); F: 107(1); G: 294(0), 365(1); H: 331(1); I: 141(0), 163(1); J: 246(0), 325(0); K: 93(1), 347(1); L: 20(1), 29(1), 45(0); M: 91(0), 235(1); N: 293(0), 314(1); O: 333(1); P: 180(1); Q: 126(1), 182(0); R: 164(1); S: 29(1), 148(0), 307(0), 347(0); T: 247(0), 370(2); U: 96(1), 141(1), 163(0). Autapomorphy of *P*. *rasbora*: 350(1).

**Phylogenetic analysis:** The analysis gave relatively divergent hypotheses, especially by instabilities of some taxa whose relationships are not in the scope of this paper. However, in a broad range of values of K between 5.9 and 23.5, most parsimonious trees ranging from 2750 to 2869 steps (CI = 0.138–0.144, RI = 0.652–0.669), results are stable concerning the new taxon. In that range of parameters, *Phycocharax rasbora* is recovered as the sister species of [*Paracheirodon axelrodi* (Schultz) + *Hyphessobrycon elachys* Weitzman] (Fig 6). Although far from conclusive, the clade recovered by *P*. *rasbora* disclosed positive GC value, suggesting a well-supported relationship. In most analysis the clade composed of *Phycocharax rasbora*, *Paracheirodon axelrodi* and *Hyphessobrycon elachys* is sister group of clade formed by *Hyphessobrycon loweae* + *H*. *vanzolinii*. It is worth noting that this latter clade is relatively little supported compared with its sister clade (i.e. *P*. *rasbora*, *P*. *axelrodi* and *H*. *elachys*). The single synapomorphy found for the *Phycocharax* clade is the abrupt expansion of the maxilla ventrally to the maxillary teeth (character 107, state 1). The section of the phylogenetic tree where *Phycocharax* is included, with clade supports and obtained synapomorphies provided (Fig 6). The complete phylogenetic hypothesis including clade supports is available in S1 Fig.

## Discussion

The characters combination of the new taxon presented by phylogenetic analysis is not shared by any known genus. According to the traditional classification of Characidae [22,23,24], *Phycocharax* could be included into the subfamily Cheirodontinae, given that it possesses a single row of aligned, expanded, and compressed premaxillary teeth. However, the new taxon proved not share the synapomorphies of Cheirodontinae according to the current phylogenetic analysis [1,25]. Cheirodontinae have as synapomorphies the absence of humeral blotch, the presence of single row of aligned, pedunculate, and similarly-shaped premaxillary teeth, and the presence of pseudotympanum between the anteriormost unmodified pleural ribs [25]. Although the new taxon shares the single premaxillary row of distally expanded teeth, they are different from cheirodontine teeth since they have irregularly sizes with the medial teeth more cuspidate and broader than the lateral ones (vs. number of cusps and shape with similar in Cheirodontinae’s teeth). Furthermore, *Phycocharax* lacks pseudotympanum and possess two humeral blotches (vs. presence of pseudotypanum and humeral blotch absent in Cheirodontinae). Cheirodontinae have as unreversed synapomorphies the presence of an anguloarticular lateral ridge and an abrupt dorsoventral expansion of the interopercular posterior region, according to Mirande[1]. Both characters are absent in *Phycocharax*.

Most non-cheirodontine and non-iguanodectine genera of Characidae have usually two or three premaxillary teeth rows. Among those taxa, only the monotypic genera *Carlana* Strand, *Erythrocharax*, *Myxiops* Zanata & Akama, and also the species *Paracheirodon axelrodi* have distally expanded premaxillary teeth with five or more cusps, similarly as is presented by *Phycocharax*.

*Carlana* shares with the remaining Rhoadsiinae the elongation of the maxilla, along with the acquisition of strong conical teeth during growth [7], missing feature in *Phycocharax* of any size observed. *Erythrocharax* has been diagnosed from remaining Characidae genera by having several features not shared by *Phycocharax*, such as the medial articulation of contralateral pelvic bones (not observed in one c&s specimen of *Erythrocharax altipinnis* examined in this study—MZUSP 119081), humeral blotch absent, presence of pseudotympanum, and absence of fifth and sixth infraorbitals bones [4]. In addition, the nasal openings of the *Erythrocharax* are separate by a narrow skin without fold, while in *Phycocharax* this character is contiguous to each other and limited by a skin fold, which is the generalized condition in Characidae. *Erythrocharax* was recovered as sister group of Rhoadsiinae and included in a clade with the Aphyoditeinae and Cheirodontinae (S1 Fig). *Myxiops* in turn is distinguishable from *Phycocharax* by having complete lateral line, and maxillary teeth edges aligned with premaxillary teeth edges, forming a continuous line between both bones [26]. In contrast, *Phycocharax* possess incomplete lateral line, and does not have the maxillary teeth forming a continuous line with premaxillary teeth. In the present analysis, *Myxiops aphos* was obtained as the sister group of a large clade including, among others, the Aphyocharacinae, Aphyoditeinae, Cheirodontinae, Gymnocharacinae, Rhoadsiinae, and Stevardiinae. The evaluation of the phylogenetic relationships of *Myxiops* could be more accurately assessed with a better taxon sampling, especially in *Deuterodon*. However, according to the analysis presented herein, *Deuterodon* is not a taxon closely related to *Phycocharax*.

The anal-fin dimorphism observed in *Phycocharax rasbora*, with males possessing a straight distal margin or almost so, is similar to the found in some species of *Hyphessobrycon* Durbin, primarily *H*. *bifasciatus* Ellis, *H*. *heliacus* Moreira, Landim & Costa, *H*. *igneus* Miquelarena, Menni, López & Casciotta, *H*. *kayabi* Teixeira, Lima & Zuanon, *H*. *loweae* Costa & Géry, *H*. *peugeoti* Ingenito, Lima & Buckup, and *H*. *procyon* Pastana & Ohara [27,28]. Between the two *Hyphessobrycon* species examined herein, *H*. *bifasciatus* and *H*. *loweae*, just this latter was herein recovered as close related to *Phycocharax rasbora*. However, the referred clade includes both species that exhibit sexual dimorphism as those that not.

The black triangular blotch on posterior flank portion of the *Phycocharax* is unique among the characids, but a similar-shaped blotch is present in *Hemigrammus pulcher*. In contrast, *H*. *pulcher* is easily distinguished from *Phycocharax rasbora* by having, as well as the remaining *Hemigrammus* species, two rows of premaxillary teeth (vs. single row). In addition, the premaxillary teeth of the new taxon are relatively compressed and distally expanded, while those of *H*. *pulcher* are relatively cylindrical with parallel lateral margins. Furthermore, *H*. *pulcher* was obtained by phylogenetic analysis in a clade along with *H*. *haraldi* Géry and *H*. *aguaruna* Lima, Correa & Ota (Fig 6), corroborating the hypothesis that *H*. *pulcher* belongs to the *Hemigrammus ocellifer* (Steindachner) species group [29,30] and, therefore, different from *P*. *rasbora*.

Phylogenetic relationships of the new taxon were supported only by a few characters in many analysis performed here. In most analysis, *Phycocharax rasbora* was recovered as the sister group of *Paracheirodon axelrodi* plus *Hyphessobrycon elachys*, in a clade that also includes *Hyphessobrycon loweae* and *Hyphessobrycon vanzolinii*. The uncertainty about the phylogenetic relationships of the new taxon, in addition to its unique combination of characters, lead us to erect a new monotypic genus in the Characidae. The erection of a new generic name when there is a stable and well-supported sister-group relationship with some available genera is undesirable in most cases, mainly when the candidates are morphologically similar. However, in this particular case, neither the phylogenetic relationships are stable or even well supported, nor the candidate genera are similar enough to include this new taxon under a common diagnosis.

## Supporting information

S1 Table Characters states of all Characidae species analyzed herein plus *Phycocharax rasbora*, new genus. Analyzed characters are listed in S2 Appendix. Polymorphisms of states 0 and 1 are denoted with a “z”.
(DOCX)

S1 Fig Phylogenetic relationships among the Characidae based in morphological data, under implied weighting with K values between 11.9 and 13.1. Numbers on nodes are GC values after a Symmetric Resampling. Clades with negative values (shown between brackets) are weakly supported.
(TIF)

S1 Appendix List of material examined. Only c&s and alcohol specimens are listed.
(DOCX)

S2 Appendix List of characters used in the phylogenetic analysis. (DOCX)
