# Supplementary material for: Phycocharax rasbora, a new genus and species of Brazilian tetra (Characiformes: Characidae) from Serra do Cachimbo, rio Tapajós basin
Source: PLoS One. 2017 Feb 15;12(2):e0170648. doi: 10.1371/journal.pone.0170648 (PMC5310855; doi:10.1371/journal.pone.0170648)
Supplement: S1 Table — Analyzed characters are listed in S2 Appendix. Polymorphisms of states 0 and 1 are denoted with a “z”. (DOCX) [file pone.0170648.s001.docx]

**S1 Table. Characters states of all Characidae species analyzed herein plus *Phycocharax rasbora*, new genus.** Characters follow Mirande [1] and Mirande et al. [2]. Polymorphisms of states 0 and 1 are denoted with a “z”.

*Phycocharax rasbora* new species.

1000010000 0010000000 1z11010000 10010001z1 000111010- 1101-000-0 0000100000 1010000000 0010000110 100-110010 0000001010 0000100000 00zz001100 0000----11 1z0000zz00 1100100100 0000000000 0000001100 0100100010 0010100110 0100000101 0000001100 1110000000 1001z10100 0100101z10 0010000000 0000000001 0010001000 1000010000 1001z00011 0000011z00 0010010100 0010------ ----001110 0000010001 1000111000 0000000000 0000?00000 0???????

Species added

*Acestrorhynchus lacustris*

1000010000 1010000000 0111110011 0001001100 000111010- 0010000001

0120100001 -01z?01100 00000?0010 0-111z0010 0000000-10 0000100000

0010001000 01?------- ----001111 0000100010 0000000010 ?010001011

1-00110011 0001101000 1100--1111 0010001100 00002?1101 0010011100

0010101100 00z0000100 0100000000 0000010001 0011110101 0010000110

0000111z00 0101001010 010------- ---0?0???? ?000000010 00?????000

0-00010000 000?0??0?0 ??11000

*Agoniates halecinus*

1000010000 0010100001 1010010011 000z000100 0001110011 0010001001

0120000000 0011001100 00z00?0010 0-0-110010 0000000-10 0000100000

0000001100 00100--0-- ----0-1111 00001000?- 0000001000 0000001010

1-00110010 0011101000 0100000101 0010001000 0100000101 0000111110

01100011z0 0010000100 0100010001 1000011000 0011110001 0010000110

0001111100 0001101000 01???????? ???0011000 0000000010 0011?1?000

0-00000000 000??0?0?0 ???????

*Atopomesus pachyodus*

1000010000 0010000z01 1010010001 00z0000101 000111010- 1101-0000?

0000100010 1010000000 00100001?? ?-??11-010 0000000011 0000100000

0010001100 0000------ -100001110 0000100000 0001000000 0100001-00

0100100010 0010100110 0100000101 1000001z00 0011000010 0001010100

0110111010 001z000001 0000000001 0000010001 0000000001 0010000110

0000011000 0100101000 01???????? ???0011100 0?00100011 0?111??001

0-00000000 000?0??0?0 ???????

*Boulengerella lateristriga*

10--0---01 -0--0?011- 0-0?-10111 000z001101 01011??10- z1101---00

01000011-- 0-10?0110? 110---01?? ??0-1---10 ??001-0-10 0000100000

0010001100 0000------ -110001111 10001?0-00 000?101001 ?01000??00

z100110010 000110z00- 110-000111 0000001000 0011001010 001000-100

00110011z0 0000000100 011000011- -001000001 00?1????00 0010000010

0??1000000 01z0100000 100------- ---??????? ?1?00??010 0????0?000

0-00000000 000?0??0?0 ??00000

*Brachychalcinus copei*

1000010000 0010000000 1011010010 1001000101 001001010- 1101-00000

0000100010 1010000000 0010000110 0-10100010 0000000z10 0000100000

0000111100 00100-1z11 0100001100 1z00z00000 001?000000 0000001100

0000100010 0010100110 0100000101 0000001100 0110000001 1001010100

0110101z10 0000000000 00001-0001 0000010000 0000001001 10z1100110

0100111100 0101101000 01???????? ???0011100 0000z??011 0?01???0??

??00000000 000?0000?0 ??11000

*Brittanichthys axelrodi*

1011010000 0010000001 1011010000 000z000111 000111110- 1101-001-0

00001011-- 10100--000 0010000-10 100-110010 0000---011 0000100000

000z001000 0000------ -110010000 --00110000 0000000010 1010001100

0100100-11 z010100110 0101000101 1000001100 0100000000 1001110100

0100111010 0010000000 00001-0001 0001010001 0000001001 0000000110

0000111001 0100111000 01110001-- -000011100 0000z0???? ???1??????

??00000000 000????0?0 11?????

*Brycon gouldingi*

1000010000 0010000001 100?-10z11 0001000100 0001010z0- 0110100000

0100100000 0111010000 1000010010 0-11000010 0000000-10 0000100000

0010101100 1011-1-011 01z1001111 1000z00101 0000000000 0000001000

0100100010 000?100010 0100000111 1000001100 0100001001 1000011100

0011001100 0000000100 0100000000 0000010001 0001110001 0011000110

0000111000 0101101000 01???????? ???0001100 00000?0010 0001?0?000

0-000?000? 000??0?0?0 ???????

*Bryconamericus stramineus*

1000011000 0010100000 1010010000 0010000101 000111010? 1101?00000

0010000000 1010000000 0011??0110 0?10100010 0000000010 0000100000

0010z11100 0010z?0011 0000001100 1z00100000 0000110000 0100001100

0000100010 0010100110 0100000101 0000001100 011z000000 1001010100

01101?0010 0011000000 00000?0000 0000010001 0000100000 0010000010

0000111000 01001010z0 0111100010 0000011100 0000000011 0001110000

0?00000000 000?000010 ??11100

*Bryconella pallidifrons*

1000010000 001000010- 1011010010 100z000101 000111010- 1101-000-0

000zz011-- 101----000 0010000--0 100-100010 000000001z 0000100000

0000001100 00100-1011 110z001100 1100100000 0011000000 0z00001101

010010--10 0010100110 0100000100 1000001100 11110000?? 1001010100

01z0101010 0000000000 00001-0001 0000010001 0000100001 001z000110

0000110000 0100101000 0111100010 0000011100 0000100010 0001110000

0000000000 000?001000 ???????

*Charax leticiae*

1000010000 0010000001 1100-10100 0000010111 000001010- 1?01-00000

0020100z00 1010000001 0010010110 0-10100010 0000000-10 0000100001

1000001000 01z0------ -110001111 00001z0000 000z000000 1000001111

1-00110011 00101001z0 1101010100 1000001100 0111000101 1011011100

0110111110 1100000000 0000000001 0000010001 0000001001 0010000110

1101111110 0100101000 0111100000 0000010--- 0000000010 0111110001

1000000000 000?001000 ??11100

*Ectrepopterus uruguayensis*

1000010000 001000000z 10110100z0 1001000101 000101010- 1101-00000

0020z00000 1010000000 0010000110 1?0-110010 0000000010 0000100000

0000001100 00z00--011 z100001110 1000100000 000z000000 0000001100

0100100010 0010100110 0100000101 0000001100 011z000000 1001010100

01001z1z10 0000000000 0000000001 0000010001 000010z001 0011000110

0000111z00 0z00101000 0111111100 1200011100 0000z00010 0001110000

0-00000000 000?00?000 0??????

*Erythrocharax altipinnis*

1010010-0- 0010000z0- 1010010010 10?0000101 000111010- 1?01-00000

000010zz-- 101-----1- 0-100-0110 100-110010 0000000011 0000100000

0000001110 0000----11 1000001100 1100z00000 0000000000 0000001000

010010--11 0010100110 0100000100 1000001101 1111000010 1001010100

0110101010 0000000000 0000010001 000001z001 0000100001 0010000110

0000111000 0100101000 010------- ---001???? ?000z00010 0??1110001

0000000000 000?00z100 ???????

*Hemigrammus aguaruna*

1000010000 0010000000 1011010000 1001000101 000111010? 1101?00000

0000z0z010 1010000000 0010000110 1?0?100010 0000000010 0000100000

00z0111100 0010001011 01000011z0 1000100000 0010000000 0z00001101

0000100010 0010100110 0100000101 0000001100 0111000000 1001010100

01001z1010 0010000000 00000?0001 0000010001 0000000001 0010000110

0000111000 0100101000 01110000?? ??10011100 0000?00010 00011??000

000000???? 000?0000?0 ???????

*Hemigrammus haraldi*

1000010000 0010000000 1011010000 0001000101 000101010- 1101-00000

0000100010 1010000000 0010000110 100-100010 0000000010 0000100000

0000001100 0010001011 0100001100 1000100000 0010000000 0000001101

0100100010 0010100110 0100000101 0000001100 0110000000 1001010100

0100111010 0010000000 0000000001 0000010001 0000000001 0011000110

0000111000 0100101000 0111000--- --10011100 0000100010 00011??000

0000000000 000?000000 ???????

*Hemigrammus marginatus*

1000010000 0010000001 1011010010 1001000101 000101010- 1101-00000

0000100010 1010000000 0010000110 100-100010 0000000010 0000100000

0000001100 00100-1011 01000011z0 1000100000 001z000000 0000001100

0100100010 0010100110 0100000101 0000001100 0110000000 1001010101

0100111010 001z000000 00001-0001 0000010001 0000000001 0011000110

0000111000 0100101000 010------- ---0011100 0000?01010 0001110000

0-00000000 000-0000?0 ???????

*Hemigrammus pulcher*

1000010000 0010000000 10110100z0 1001000101 000101010? 1101?00000

00001000z0 1010000000 0010000110 100?100010 0000000010 0000100000

0010011100 0010001011 0100001100 1000100000 001z000000 0z0000110z

0000100010 0010100110 0100000101 0000001100 0110000000 1001010100

0100101010 0010000000 0000000001 0000010001 0000000001 0011000110

0000111000 0100101000 01110000?? ??10011100 0000100010 00011??000

0000000000 000?00z000 ???????

*Hemigrammus rodwayi*

1000010000 0010000001 1011010010 1001000101 000111010? 1101?00000

0000100010 1010000000 0010000110 1?0?100010 0000000010 0000100000

0000001100 0010001011 0100001100 1000z00000 0011000000 0000001100

0100100010 0010100110 0100000101 0000001?00 0110000000 1001010101

01z01z1010 0011000000 0000000001 0000010001 0000000001 0011000110

0000111000 0101101000 01???????? ???001??00 0000???010 0001??????

????000000 000?00?0?0 ??11000

*Hyphessobrycon compressus*

1000010000 0010000000 1011010010 z001000101 000101010- 1101-00000

0000100z10 101000-000 0010000110 100-110010 0000000010 0000100000

0000001100 00100-1011 0100001110 1000?00000 0000000000 0000001100

0100100010 0010100110 0100000101 0000001100 01z0000000 1001010100

01z0111010 00z0000000 00000?0001 0000010001 0000z0z001 0011000110

0000111z00 0100101000 01110000-- -000011??? 0?10000010 0001110000

0-00000100 000?00?000 0??????

*Hyphessobrycon erythrostigma*

1000010000 0010000001 1011010010 1001000101 000001010? 1101?00000

0001000010 1010000000 0010000110 100?110010 0000000010 0000100000

0000001100 0010001011 1100001110 0000100000 0011z00000 000000110z

0100100010 0010100110 0100000101 0000001100 0100000101 1001010100

0100111010 0010000000 0000000001 0000110001 0000000001 0011000110

0000111100 010z101000 01???????? ???001z100 0000???010 0001??????

????000000 000?00?0?0 ??11100

*Hyphessobrycon loweae*

1000010000 001000000z 1011010010 1001000101 000111010- 1101-00000

0000000010 101000--00 0010000110 100-110010 0000000010 0000100000

0000001100 00100-1111 0100001z00 1100100100 0000000000 0z0000110z

0100100010 0010100110 0100000100 0000001100 0110000000 1001010100

01001?1010 00z0000000 0000010001 0000010001 0000000001 0010000110

0000111000 0100101000 010------- ---001?100 0000100010 0??1110000

0000000000 000?00?000 ???????

*Hemigrammus* aff. *lunatus*

1000010000 0010000000 101z010000 1001000101 000101010- 1101-00000

0000100010 101000--00 0010000110 100-100010 0000000010 0000100000

0000011100 00100-1011 0100001100 1100100100 00zz000000 000000110z

0100100010 0010100110 0100000101 0000001100 0111000000 1001010100

0110111010 0010000000 0000000001 0000010001 0000z00001 0011000110

0000111000 0100101000 0111000--- --00011100 0000000010 00011??000

00000z0000 000?00z000 ???????

*Hyphessobrycon moniliger*

1000010000 0010000001 1011010010 1001000101 000101010- 1101-00000

0000100000 101000--00 0010000110 100-110010 0000000010 0000100000

0000001100 00100-1011 01000011z0 1000100000 0001000000 000000110?

0100100010 0010100110 0100000101 0000001100 0110000000 1001010100

0110101110 0010000000 0000000001 000-110001 000000z001 0010000110

0000111z00 0100101000 010------- ---0011100 0000z00010 00011??000

000000?000 000?00z0?0 ???????

*Hyphessobrycon montagi*

1000010000 0010000001 1011010000 1001000101 0001z1010- 1101-00000

0000100010 1010000000 0010000110 100-100010 0000000010 0000100000

00z0001100 0010001011 01000011z0 1000100000 0011000000 000000110z

0100100010 0010100110 0100000100 1000001100 0110000000 1001010100

0100101010 0010000000 0000000001 0000010001 0000000001 0011000110

0000111000 0100101000 010------- ---0011100 0000z00010 00011??000

000000z000 000?000000 ???????

*Hyphessobrycon vanzolinii*

1000010000 001000010- 1011010010 0001000101 0001z1010- 1101-00000

00z0000010 1010000000 0010000110 100-110010 0000000010 0000100000

0000001100 0010001111 11000011z0 1100000100 0001000000 0000001100

0100100010 0010100110 0100000101 0000001100 0110000z01 1001z10100

0110101010 00z0000000 0000000001 0000010001 0000000001 0011000110

0000111000 0100101000 010------- ---0011100 0000z00010 00011??000

000000???? 000?001000 ???????

*Moenkhausia cf. cotinho*

1000010000 001000010- 1011010010 1001000101 000101010- 1101-00000

0000100010 1010000000 0010000110 0-10100010 0000000z10 0000100000

00z0001100 00100-1011 0100001100 1100100000 001?000000 0000001000

0100100010 0010100110 0100000101 1000001100 0110000000 1001110100

0100101010 0010000000 0000000001 0000010001 0000000001 0011000110

0000111000 0100101000 01???????? ???0011100 0000z?1010 0?01???0??

??000000?? 000?0??0?0 ???????

*Moenkhausia heikoi*

1000010000 0010000001 1011010010 0001000101 000101010- 1101-00000

0000100010 1010000000 0010000110 0-10100010 0000000010 0000100000

0000001100 00100-1011 0100001110 1000100000 00z1000000 0000001100

0100100010 001??????? ?10???0??? ?????????? ??10?????? ???1z10100

0110101010 0010000000 0000000001 0000010001 0000001001 0011000110

0000111100 0101101000 01???????? ???0011100 00?0z0z010 0?01?1?000

0-00000000 000??0?0?0 ???????

*Moenkhausia jamesi*

1000010000 001000010- 1011010010 1001000101 001001010- 1101-00000

0000100010 1010000000 0010000110 0-10100010 000000zz10 0000100000

0010z01100 00100-1010 010000z000 1z00100000 00z?000000 0000001100

0000100010 0010100110 0100000101 000000110z 1110000000 1001z10100

0110111010 0010000000 0000??0001 0000010001 0000001001 0011000110

0000111100 0101101000 01???????? ???0011100 0000??1011 0001???000

0-00000000 000?0?00?0 ???????

*Moenkhausia lata*

1000010000 0010000001 1011010010 1001000101 000101010- 1101-00000

0000100010 1010000000 0010000110 0-10100010 0000000110 0000100000

0000001100 00100-1011 0100001000 1000z00000 0010000000 0000001100

0100100010 0010100110 0100000101 0000001100 0110000000 1001110100

0100101010 0010000000 0000000001 0000010001 0000000001 0001000110

0000111100 0101101000 010------- ---0011100 0000??1010 00011??000

0-0000000- 000?00?0?0 ???????

*Myxiops aphos*

1000010000 001000010? 1011010000 0000000111 000111010? 1101?00000

00001011?? z010000000 0010000110 0?10110010 000001z010 0000100000

0000001100 0000????11 1100001100 1100100100 0000000000 0z00001100

0000100010 0010100110 0100000101 0000001100 0110000000 1001?1?100

0110??1010 00z0000000 0000000001 0000010001 0000100001 0011000110

000011z000 0101101000 010??????? ???0011100 0000000011 0001110000

0?00000000 000?0000?0 ???????

*Orthospinus franciscensis*

1000010000 0010000000 1011010010 1001000101 00z001010- 1101-00000

0000100010 1010000000 0010000110 0-10100010 0000000010 0000100000

0000001100 00100-1010 0100001z00 1100100000 00z?000000 ?000001100

0000100010 0010100110 0100000101 0000001100 0110000001 1001010100

0110101010 0010000000 00001-0001 0000010001 0000001001 00z1100110

0100111100 0100101000 011111z000 0200011100 00?00??010 0?01???0??

??00000000 000?0000?0 ??11000

*Petitella georgiae*

100001?000 001000010- 1011010010 100z000101 000111010- 1101-00000

0000100010 1010000000 0z10000110 100-110010 0000---011 0000100000

0000001100 0010---011 111000000- ---0zz0000 0001000000 000000110z

0100100010 0010100110 0100000101 0000001100 0110000000 1001010100

0110101010 0010000000 0000000001 0000010001 0000000001 0001000110

0000110000 0100101000 01110000-- -010011100 0000?010?? ?001010000

0-00000000 000?000000 ???????

*Salminus hilarii*

1000010000 001000011- 100?-10z?1 0000010000 000111010- 1010100000

0120z00000 01110z0001 10000?0010 0-11100010 0000000-10 0000100000

0000001100 00100-?01- -110001111 z000100010 0000000000 0000001001

1-00110010 0000100010 1100z00111 1000001100 0110001101 0000011100

0011001100 0000000100 0000000001 000001000z 0001110001 1011000110

0000111z00 0101101010 01???????? ???000??0? ?0?00??010 0001???0??

??00000000 000????0?0 ??11000

*Tetragonopterus chalceus*

1000010000 0010000001 1011010010 0001000101 00z001010- 1101-00000

0000100010 0010010000 0010000110 0-11100010 0000000110 0000100000

0000001100 00100-1011 01000011z0 1000100000 0011000000 0000001100

0000100010 0010100110 0100000101 0000001100 0110000001 1001010100

0100101010 0000000000 0000000001 0000010001 0000001001 0011000110

0000111100 0101101000 01???????? ???0111101 0000??1010 0001???0??

??00001000 000??0?0?0 ??11100

Remaining species

*Puntigrus tetrazona*

----1---00 -?0-00zzz- 1-0---3101 00?z000100 010-----0- 0--001-100

0---1----- -----0000- -100--00z0 ?-0-11---- ??-?---0z1 0??-0-000-

00?0-01--- 0--------- ------0000 ---------- -----0-000 ?0000z0--0

0-00101-10 001?100-00 000-00-?00 1001???101 111100-010 100000-000

00zz0010-- 000000000- 10?001zz0- -01-000001 100-zz1000 0001000101

0010000000 0000100000 000------- ---00z1001 0100z000?- -z?001z000

0-00000000 000?0210?z ??1zzz0

*Acestrocephalus sardina*

1000010000 0010000z01 1000-10zz0 00?001?101 000101010- 0001-00000

0020100001 001z010001 00100z0110 0-10100010 0000000-10 0000100001

?0z0001000 01100--0-- -000001111 0000100010 00-0000000 1000001111

1-00111011 0000100100 1101010100 1000001100 1111000101 1011011100

01101111?0 1000000000 00000?0001 0?00010001 0000000001 0010000110

00011111z0 010z1010z0 0111?????? ???2010--- 0000010010 0111110001

1100000000 000?0010?0 ???????

*Acestrorhynchus pantaneiro*

1000010-01 1010000z0? 0011110011 000z011100 00011101z- 0010100001

0120100001 -01z001?00 0010000010 0-11100010 0000000-10 0000100000

0000001000 01?------- ----001111 0000100010 000?000010 1010001011

1-00110011 0001101000 1100--1111 0010001100 00002?1101 0010011100

0011101100 0010000100 0100010000 0000010001 001z110101 0010000110

0000111000 0z010010z0 010------- ---0000--- 0000000010 0011110000

0-00010000 00000010?0 0?11000

*Acrobrycon ipanquianus*

1000011000 001010000z 1010010000 00z000010z 000111010- z101-00000

0000000000 1010000000 0011--0110 0-10100010 0000000010 0000100000

00z0011100 00100-1011 000000111z 1000100000 0000010000 0z00001100

0000100010 0010100110 0100000101 0000001100 0110000000 1001010100

0110z01010 0010000000 0000010000 0000010001 0000110000 0010000010

0000111z00 0100101000 0111100111 0000011100 0000000110 0001110000

0-00000000 0001000010 11?????

*Agoniates anchovia*

1000010000 0010?00001 1010010011 0000000100 0001110011 0010001001

0120000000 0011001?00 00?0010010 0-0-1?0010 0000000-10 0000100000

0000001100 00100--0-- ----0-1111 00001000?- 000?001000 0000001010

1-00110010 0011101000 0100000101 0?10001100 0100000101 0000111110

01110011?0 0011000100 0100010001 1?00011000 001111z001 0010000110

00?1111100 0001101000 01???????? ???0011000 0000000010 0011110000

0-00000000 000?0010?0 ???????

*Alestes macrophthalmus*

100000-00? 0????0010- 1000-121?1 ?0?1000?00 0001010?11 01?0?0?100

???????0?0 001??1000? 11?0??0?11 0-??01010? ??-?---210 0??0110000

0??010?100 0010?--020 0000000000 --00?00?00 000??01000 ?10??01000

000?00?010 ?0?1100000 010?0001?? 1000001100 ??000000?? ?000?1?1?0

011100??00 00?0000100 01000?000z 0?000?0001 100110?000 0000000111

0110011000 0111000000 100------- ---00?1001 0000000011 10?1?00000

0-00?00000 000??0?0?0 ???????

*Apareiodon affinis*

0---10--00 000-10111- 1-00-11101 0000010100 0101110011 0110100100

0-?0000010 0010000000 11000-0000 0-0-11?010 0000010001 00000-1000

0-100011-0 00?------- ----1-1100 110------- ---1100000 0000001000

0000100011 1001100000 0000000001 100??01?01 110010-010 1000?0-100

0011000000 0011000100 00000?0001 010000?001 000z101001 1001000111

0010000000 0101101000 000------- ---0001001 010000000- -?-0011000

0-00000000 000?0110?0 ??11110

*Aphyocharacidium bolivianum*

1010011000 001000010- 1010010000 01?z000101 000111010- 1101-00000

0010000010 1010000000 0010000110 0-0-110010 0000000011 0000100000

0001001100 0000----11 1110011110 1000110010 0001100000 0000001100

0100100010 0010100110 0101000101 0000001100 1111000000 1001010100

01101z1010 0010000000 00000?0001 0?00010001 0000000001 0010000110

0000z1100z 01001010z1 0111100010 100001???? 0000000010 00?1z??001

0000000000 000?00?010 ???????

*Aphyocharax anisitsi*

10000--100 001011010- 1010100100 0000001111 100111010- 1101-00000

00100001-- 0010001100 0z10000110 1110111010 0000000010 0001100000

0001001100 0000----1- -1000111z0 0000100000 0000100000 0000001100

0100100010 0010100110 0100000101 0000001100 0111000000 1001010100

0100111000 0010000000 00001-0z00 1000010000 0000100001 0010000110

000011z000 0100101000 011110001? 1000011100 0000000010 0001110000

0-00000001 0000000010 ??11000

*Aphyocharax dentatus*

10000--100 001011010- 1010100100 0000001111 100111010- 1101-00000

00100001-- 0010001100 0010000110 1110111010 0000000010 0001100000

0001001100 0000----1- -1z0011110 0000100000 0000100000 0100001100

0100100010 001010011z 0100000101 0000001100 0111000000 1001010100

0110111000 0010000000 00001-0000 1000010001 0000100001 0010000110

0000111000 0100101000 0111100000 0000011100 0000000010 000111z000

0-00000001 000?000010 ??11000

*Aphyocharax nattereri*

10000---00 001011000z 1000-00100 0000001111 100111010- 1101-00000

00100011-- -01000?11- 0-10000-10 110-111010 0000000-10 0001100000

0000001100 0000----1- -100011110 0000100000 000?000000 0000001100

0100100010 0010100110 0100000101 0000001100 0111000000 1001010100

01001110-0 0010000000 00001-0000 100001000z 0000100001 0010000010

0000111000 0100101000 0111100000 0000011100 0000100010 000111?000

0-00000000 0000000000 0??????

*Aphyodite grammica*

1010010000 0010000000 101z010000 0001000101 000111010- 1101-00000

00101011-- 1010000000 0010000110 100-110010 0000000011 0000100000

0000001100 0000----1- -1100?1z00 0000110000 0001100000 0000001100

0100100010 0010100110 0100000101 0000001100 1110000000 1001010100

0100111010 0010000000 00001-0001 0000010001 0000100001 001z000110

0000111000 0100101000 01111????? ??00011100 0000101010 01?1110001

0000000000 000?00?0?0 ???????

*Argopleura magdalenensis*

1000011000 0010100000 1010010000 0010000101 000101010- 1101-00000

00100000z0 z010000000 0010000110 0---100010 0000000010 0000100000

00z0z11100 00100-101z 0000001z00 1000100000 00000z0000 0100001100

0000100010 0010100110 010000010z z000001100 1110000010 1001z10100

011z101010 0011000000 0000z-000z 000z01000z 0000100000 0010000010

0000111100 01001010z0 01110000-- -000011100 0000000110 0001110000

0-00000000 000?z00010 11?????

*Astyanacinus moorii*

1000010000 0010000000 1010010000 10?1000101 000z01010- 1101-00000

00x0100000 0011000000 0010000110 0-10100010 0000000110 0000100000

00100z1100 00100-1011 01000011z0 1100100000 0010000000 0000001100

00001z0010 0010100110 0100000101 0000001100 0110000001 1001010100

0110101110 0000000000 0000000001 00000100?1 0000000001 00110??110

0000111100 0100101000 01???????? ???0001100 0000000010 0001110000

0-00010000 000?1000?0 ???????

*Astyanax abramis*

1000010000 0010000000 1000?10000 0001000101 000z01010? 1101?00000

0000100000 0011000000 0010000110 0?10100010 0000???110 0000100000

0010111100 00100?1010 0100000000 ??00z00100 00z00z0000 0000001100

0000100010 00z0100110 0100000101 0000001100 01z0000000 1001010100

0110101010 0000000000 00000z0001 0000010001 0000z01001 0011000110

0000111100 0100101000 01111zzz0z 000000110z 0000000011 0001110000

0?00010000 010?101000 ??11000

*Astyanax altiparanae*

1000010000 0010000z00 1000-10000 0001000101 000z01010- 1101-00000

0000100010 0011000000 0010000110 0-10100010 0000---110 0000100000

0010111100 00100-101z 0100000000 --00000100 00000z0000 0000001100

0000100010 0010100110 0100000101 0000001100 0110000000 100101z100

0110101010 0000000000 0000000001 0000010001 0000z01001 0011000110

0000111z00 0100101000 01111????0 0??0001101 0000000011 0001110000

0-00010000 010010z0?0 ??11000

*Astyanax asuncionensis*

1000010000 0010000000 1000?10000 0001000101 000101010? 1101?00000

0000100010 0011000000 0010000110 0?10100010 0000???110 0000100000

0010111100 00100?1010 0100000000 ??00z00100 00z00z0000 0000001100

0000100010 00z0100110 01z0000101 z000001100 0110000000 1001010100

0110101010 0000000000 0000000001 0000010001 0000z01001 0011000110

0000111z00 0100101000 01111zzz0z 000000110z 0000000011 0001110000

0?00010000 010?10z000 ??11000

*Astyanax cf. eigenmanniorum*

1000010000 001000000z 1010010000 z001000101 000101010? 1101?00000

0000100010 0010000000 0010000110 0?10100010 0000??0110 0000100000

00z0011100 00100?1111 0100001000 1z00100100 0000000000 0000001100

0000100010 0010100110 0100000101 z000001100 0110000000 1001010100

0110101010 0000000000 0000000001 0000010001 0000101001 0011000110

0000111000 0100101000 0111100000 0000011100 0000000010 0001110000

0?00000000 000?00?000 ???????

*Astyanax cf. rutilus*

1000010000 0010000001 1010010000 0001000101 000101010- 1101-00000

0000100010 0010000000 0010000110 0-10100010 0000--0110 0000100000

00z00z1100 00100-1z11 0100001000 1100100100 0000000000 0000001100

0100100010 0010100110 0100000101 0000001100 0100000000 100101z100

0110101010 0000000000 0000000001 0000010001 0000101001 0011000110

0000111100 0100101000 0111100000 0000011100 0000000010 0001110000

0-00000000 000?000000 ???????

*Astyanax chico*

1000010000 0010000000 1010010000 0001000101 000101010- 1101-00000

0000100010 001z000000 0010000110 0-10100010 0000--0110 0000100000

0010011100 00100-1111 0100001000 1z00z00100 000?000000 0000001100

0000100010 0010100110 0100000101 0000001100 0110000001 1001010100

0110101010 0000000000 0000000001 0000010001 0000101001 0011000110

0000111000 0100101000 0111111100 0000011100 0000000011 0001110000

0-00000000 000?001000 ???????

*Astyanax correntinus*

1000010000 0010000001 10z0010000 0001000101 000z01010- 1101-00000

0000100010 0010000000 0010000110 0-1z100010 0000--0110 0000100000

0010011100 00100-1110 0100001000 1100100100 0000000000 0000001100

0000100010 0010100110 0100000101 0000001100 0110000001 1001010100

0110101010 0000000000 0000000001 0000010001 0000001001 0011000110

0000111100 0100101000 01111000?? ??00011100 0000000010 0001110000

0-000z0000 000?100000 ???????

*Astyanax endy*

1000010000 0010000000 1010010000 0001000101 000101010- 1101-00000

0000100010 0010000000 0010000110 0-10100010 0000000110 0000100000

00z0011100 00100-1111 0100001z00 1z00100100 0000000000 0000001100

0000100010 0010100110 0100000101 0000001100 0110000000 1001010100

0110101010 0000000000 0000000001 0000010001 0000101001 0011000110

0000111000 0100101000 0111100000 0000011100 0000000011 0001110000

0-00000000 000?000000 ???????

*Astyanax latens*

1000010000 0010000000 10z0010000 z001000101 000101010- 1101-00000

0000100010 1010000000 0010000110 0-10100010 0000000110 0000100000

00z00z1100 00100-1011 01000011z0 1000100000 0010000000 0000001100

0100100010 0010100110 0100000101 0000001100 0100000000 1001010100

01101z1010 0000000000 0000010001 0000010001 0000101001 0011000110

0000111100 0100101000 0111100000 0000011100 0000000010 0001110000

0-00000000 000?000000 ???????

*Astyanax lineatus*

1000010000 0010000000 1000-10000 000z000101 000101010- 1101-00000

0000100000 0011000000 0010000z10 0-10100010 0000--0110 0000100000

0010111100 00100-1010 0100001000 1z00z00100 00z?100000 0000001100

0000100010 0010100110 0100000101 0000001100 0110000000 1001010100

0110101010 0000000000 0000000001 0000010001 0000z01001 0011000110

0000111z00 0100101000 0111111100 0000001100 0000000011 0001110000

0-00000000 0000100000 0?11000

*Astyanax mexicanus*

1000010000 0010000z00 10z0010000 0001000101 000101010- 1101-00000

0000100000 0010000000 0010000110 0-10100010 0000--0110 0000100000

00z0011100 00100-101z 0100001000 1100100100 0000000000 0000001100

0100100010 0010100110 0100000101 0000001100 0110000000 1001010100

0110101z10 0000000000 0000000001 0000010001 0000z01001 0011000110

0000111000 0100101000 011110000z 0000001100 0000000010 0001110000

0-00000000 000?00?000 ??11000

*Astyanax paris*

1000010000 0010000000 1010010000 0001000101 000101010- 1101-00000

0000100000 0010000000 0010000110 0-10100010 0000000110 0000100000

0010001100 00100-1011 01000011z0 1000100000 0010000000 0000001100

0000100010 0010100110 0100000101 0000001100 0110000000 1001010100

0110101010 0000000000 0000000001 0000010001 z000101001 001z000110

0000111000 0100101000 010------- ---0011100 0000000010 0001110000

0-00000000 000?000000 ???????

*Astyanax pelegrini*

1000010000 0010000000 1010010000 0001000101 000101010- 1101-00000

0000100010 0010000000 0010000110 0-11100010 0000--0110 0000100000

0010111100 00100-1010 0100001000 1100z00000 0000100000 0000001100

0000100010 0010100110 0100000101 0000001100 0100000000 1001010100

0110101010 0000000000 0000000001 0000010001 0000001001 0011000110

0000111110 0100101000 0111100000 0000001100 0000000011 0001110000

0-00000000 000?100000 ???????

*Astyanax puka*

1000010000 0010000000 1010010000 z001000101 000101010- 1101-00000

0000100010 0010000000 0010000110 0-10100010 0000--0110 0000100000

00z0011100 00100-1111 0100001000 1100100100 0000000000 0000001100

0000100010 0010100110 0100000101 0000001100 0110000001 1001010100

0110101010 0000000000 0000010001 0000010001 0000z01001 0011000110

0000111z00 0100101000 0111100000 0000011100 0000000011 0001110000

0-00000000 000?000000 ???????

*Astyanax troya*

1000010000 0010000000 1010010000 z001000z01 000101010- 1101-00000

0000100000 0011000000 0010000110 0-10100010 0000--0110 0000100000

0010011100 00100-1111 0100001000 1100100100 0000000000 0z00001100

000010z010 0010100110 0100000101 0000001100 011z000001 1001010100

0110101z10 0000000000 0000010001 0000010001 0000z01001 0011000110

0000111000 0100101000 0111111100 0000011100 0000000011 0001110000

0-000z0000 000?00z000 ???????

*Attonitus ephimeros*

1000011000 0010100z0? 1000-10000 0010001101 000111010- 1101-00000

0010000000 1010001000 0011--0110 0-10110010 0000000011 0000100000

0010001100 00100-1011 1000001110 1000z00000 0000101000 0100001100

0000100010 0010100110 0100000100 0000001101 1111000010 1001010100

0110101010 0011000000 0000010000 0000010001 0000100000 0010000010

0000110000 0100101000 0111100011 0000011100 0000000010 0001111000

0-00000000 000?00?010 10?????

*Aulixidens eugeniae*

1000010000 000010010- 1010010000 001z000101 00011101?? 1101-00000

0010000000 1010000000 0011--0110 0-10110001 0000---011 0z00100000

0010011100 0000----11 1000000000 --00000100 000?100000 ?100001100

0100100010 0010110110 0100000101 0000001100 1111000010 11-1010100

0110110010 0010000000 0000z-0000 0000010001 0000100000 0010000z10

0000111000 0100101000 0111100011 1000011100 0000001011 0??1???0??

0-00000000 000?0000?0 ???????

*Axelrodia lindeae*

1010010000 0010000001 1010010000 01?z000101 000111010- 1101-00000

00-0100010 -010000000 0010000-10 100-110010 0000000011 0000100000

0000001000 0000------ -110011110 0000110000 0101100000 0000001z00

0100100-1z 0010100110 0101000100 1000001000 0111000010 1001010100

0110111010 0010000000 0000000001 0?00010000 0000000001 0010000110

0000111z01 01001011z1 0111100011 000001???? 0000000010 0??1z??001

0000000000 000?00?010 ???????

*Bario steindachneri*

1000010000 001000010- 1011010010 1001000101 000111010- 1101-00000

00001000z0 ?010000000 01100?0110 0-??100010 0000000010 0000100000

0000011100 00100-1011 0100001z00 1z00100000 001?000000 0000001100

0000100010 0010100110 0100000101 0000001100 0110000000 1001010100

0100101010 0000000000 0000000001 0000010001 0000100001 0010000110

0000111000 0101101000 0111111101 1200z11-11 0000z11010 00011??0??

0-00000000 00z??0?0?0 ???????

*Bramocharax bransfordii*

1000010000 0010000000 1011010000 0001000101 000111010- 11?1-00000

0000100000 0010000000 00100?0110 0-??100010 0000000-10 0000100000

00z0011100 00100-1011 0z00001111 1100100000 0000000000 0000001100

0000110010 0010100110 0100000101 0000001100 0110000001 1001010100

0110101110 0000000000 0000000001 0000010001 0000001001 0011000110

0000111100 0100101000 011??????? ???0011100 0000000010 0?011??000

0-00000000 000?00?0?0 ???????

*Brycinus carolinae*

100000-000 00???0011- 1-0--121?1 ?0??000100 010111?111 0??0100100

0?00000000 001001000? 11????0?11 0-0-010100 ????---21? 0??0110000

0110100100 0010?--020 0000000000 --00000?01 000?101000 ?100001000

000100?010 00111??000 ?10??001?? 100000110? ?1000000?? ?000?111?0

001000???0 0000000100 01000?0001 0?000?0001 1001100000 0000000111

0110010000 0111?00000 100------- ---0001001 0000100011 1??11??000

0-00000000 000??010?0 ??10000

*Brycon falcatus*

1000010?00 0010000001 101??100?1 0001000z00 0001110z0- 0110100000

???0100000 0111010000 10000?0010 0-11000010 0000000-10 0000100000

0010?01100 1011-1-011 0101001111 1000100101 00z?000000 ?000001000

00001??010 0000100000 01000001?? 1000001100 0100001001 1000011100

0111001100 0000000100 010000000? ?000010001 0001110001 0011000110

0000111000 0101101000 011??????? ???0001100 0000010010 00?1???0??

?-00000000 000??0?0?0 ??11000

*Brycon meeki*

1000010?00 ??10??0001 101?0100?1 0001000000 0001010z0- 01?0100000

0?00100000 000101000? ?0000?0?10 0-??000010 ??00000-10 0??0100000

0010?01100 1011-1-01? 010?001111 1000100?01 00??00?000 ?00000100?

0?00100010 00?0100?00 0100000111 100?001100 ??0000?001 ?0000111?0

0z11001?00 0000000?00 01000?0000 0?00010001 000111?001 0010000110

0000111100 0111101000 01???????? ???00????? 00000?001? ?0?????000

0-00000000 000??0?0?0 ???????

*Brycon orbignyanus*

1000010000 0010000001 1000-100?1 0001000100 000z01000- 0110100000

0100100000 00z1010000 10000?0010 0-11000010 0000000-10 0000100000

0010101100 1011-1-111 0111001111 1100100101 0000000000 10000010z0

0000100010 0000100010 0100000111 1000001101 1100001001 1000011100

0111001100 0000000100 0000000000 0000010001 0001110001 0011000110

0000111z00 0101101000 01???????? ???0001100 00000?0010 0001100000

0-00010000 000?00?0?0 ??11000

*Brycon pesu*

1000010?00 001000011- 1011010011 0001000100 0101110z0- 0110100000

0100000000 00z1010z00 ?000000010 0-11000010 0000000-10 0000100000

0010101100 1011-1-011 0101001111 1000100101 00z0000000 z000001000

00001z0010 00001000z0 0100000111 100z001100 0110001001 1000011100

0110001000 0000000100 0100000000 0000010001 0001110001 001z000110

0000111000 0101101000 01110000-- -0?0001100 0100010010 0?01?1?000

0-00000000 00000010?0 0?11000

*Brycon polylepis*

1000010000 0010000001 10100100?1 0001000100 000111000- 0110000000

0100100000 0001010000 10000?0010 0-??000010 0000000-10 0000100000

0010101100 1011-1-010 0101001111 10001z0101 0000000000 ?100001000

0100100010 00z01000z0 0100000111 1000001100 0100000001 1000011100

0z11001z00 0010000100 0z00000000 0000010001 0001110001 0011000110

0000111100 0101101000 01???????? ???0011100 00?00??011 0?01???000

0-0000000? 000?00?0?0 ???????

*Bryconadenos tanaothoros*

1000011000 001010010- 1010010000 0010000101 000111010- 1101-00000

0010000000 101000z000 0011--0110 0-101z0010 0000000010 0000100000

0010111100 00100-1011 0000001100 1100000000 0000100000 01000011z0

0000100010 0010100110 0100000100 z000001100 111z000010 1001010100

01101z1010 0011000000 00z0000000 0000010001 0000100000 0010000010

000011100z 0100101000 01110000-- -000011100 0000000011 0001???000

0-00000000 000100?010 1??????

*Bryconaethiops macrops*

100000-000 00??0001z- 1-0--121?1 00?1000100 0001010011 0110100100

??000000?0 0?1001000? 1100??0?10 0-0-010100 ????---210 0??0110000

0?10100100 ?011-?-020 0000000000 --00000?01 000?101000 ?100001000

0001000010 00111??000 ?10??001?? 100??01101 11000000?? 10001111?0

011?001000 0000000100 01000?000z 0?1?000001 100100?000 0000000111

0110011000 0111000000 100------- ---0001001 0000100011 10?11??000

0-00000000 000??0?0?0 ???????

*Bryconamericus agna*

1000011000 0010100000 1010010000 0010000101 000111010- 1101-00000

0010000000 1010000000 0011--0110 0-10110010 00000z0010 0000100000

0010111100 00100-1111 0000001100 1100000100 0000100000 0100001100

0000100010 0010100110 0100000101 0000001101 1111000000 1001010100

0110101010 0010000000 0000z-0000 0000010001 0000100000 0010000010

0000111000 0100101000 0111100000 0000011100 0000000011 0001110000

0-00000000 000?000010 ???????

*Bryconamericus cf. iheringii*

1000011000 0010100000 1010010000 0010000101 000111010- 1101-00000

0010000000 101000z0z0 0011--0110 0-10110010 0000000010 0000100000

0010011100 00100-1011 00000011z0 1000z00000 0000100000 0z00001100

0000100010 0010110110 0100000101 0000001101 1110000000 1001010100

0110101010 0010000000 0000000000 0000010001 0000100000 0010000010

000011z000 0100101000 0111111011 0000011100 0000000011 0001110000

0-00000000 000?000010 0?11100

*Bryconamericus emperador*

1000010000 0010100000 1000?10000 00z1000001 000101010- 1101-00000

00z0000000 0010000000 0010000110 0-10100010 0000000110 0000100000

0010011100 0010z-0011 0000001100 1000100000 0010000000 0000001000

0000100010 0010100110 0100000101 0000001100 0110000000 1001010100

0110101010 0000000000 0100000001 0?00010001 0000100001 0011000110

0000111100 0101101000 0111111z?? ??00011100 0000000010 0001110000

0-00000000 000?0010?0 ???????

*Bryconamericus exodon*

1000011000 001010000z 1010010000 001z000101 000111010? 1101?00000

0010000000 1010000000 0011??0110 0?10100010 0000000010 0000100000

0010111100 00101?0011 0000001100 1000100000 00001z0000 0100001100

0000100010 00101z0110 0100000101 z000001100 z11z000000 1001010100

01101z0010 0011000000 0000000000 0000010001 0000100000 0010000010

0000111000 010z101000 01111000z0 0000011100 0000000011 0001110000

0?00000000 0000000010 0?11100

*Bryconamericus mennii*

1000011000 0010100000 1010010000 0010000101 000111010- 1101-00000

0010000000 101000z000 0011--0110 0-10100010 0000000010 0000100000

0010z11100 00100-1011 00000011z0 1000z00000 0000100000 0100001100

0000100010 0010100110 0100000101 0000001100 0111000000 1001010100

0110101010 0010000000 0000000000 0000010001 0000z00000 0010000010

0000111000 0100101000 010------- ---0011100 0000000011 0001110000

0-00000000 00000000?0 ???????

*Bryconamericus rubropictus*

1000011000 001010000z 10100z0000 0010000101 000111010? 1101?00000

0010z00000 101000z0z0 0011??0110 0?z0110010 0000000010 0000100000

0010011100 00100?1011 0z000011z0 1000000000 0000100000 0z00001100

0000100010 0010110110 0100000101 0000001101 111z000000 1001010100

01101z1010 0010000000 0000000000 0000010001 0000100000 0010000010

0000110000 0100101000 01111zz011 0000011100 0000000011 0001110000

0?00000000 000?001000 ???????

*Bryconamericus scleroparius*

1000010000 001010010- 1000-10000 0001000101 000101010- 1101-00000

0000000000 0011000000 0010000110 0-10100010 0000000110 0000100000

0010011100 0010z-1011 0000001100 1100100100 0000000000 0000001000

0000100010 0010100110 0100000101 0000001100 011z000000 1001010100

0110101110 0000000000 0000000001 0000010001 0000101001 0011000110

0000111100 0101101000 01111110?? ??00011100 0000000010 0001110000

0-00000000 000?00?0?0 ???????

*Bryconamericus thomasi*

1000011000 001010000z 1010010000 0010000101 000111010- 1101-00000

0010000000 1010001000 0011--0110 0-0-110010 0000000010 0000100000

0010011100 00100-1011 00000011z0 1000z00000 0000100000 0z00001100

0000100010 0010110110 0100000101 0000001101 1111000000 1001010100

0110101010 0010000000 0000000000 0000010001 0000100000 0010000010

000011z000 01001010z0 0111101011 0000011100 0000000011 0001110000

0-00000000 000?00z010 ???????

*Bryconexodon juruenae*

1000010000 1010000001 1011010010 10000z0001 0001010z?? 1101-00001

0010100000 0011010000 0010000110 0-10100010 0000000-10 0000100000

00z0001101 00??------ ----001111 0000-00010 00-0000000 0000001100

0000100010 0010100110 0100000100 1000001100 1111000001 1001z10100

0110101?10 0000000000 0000z00001 0?00010001 0000100001 0011000110

0000111000 0101101000 0111?????? ??00011100 0000000010 00?1?1?0??

?-00000000 000?0000?0 ???????

*Bryconops affinis*

1000010000 0010000001 1011010011 0001000100 000111010- 0110000000

1100100010 0111010000 1010000011 0-0-100010 0000000210 0000100000

0000001100 00100-101z 0100001100 1z00100101 0011000000 0000001000

0000100010 0011100010 0100000111 1000001100 0110000001 1001110100

0111001000 0010000100 01000z0000 0000010001 0001100001 00z0000110

0000111z00 0101101010 0111100000 0000011100 0000000010 0001110000

0-00000000 000?00?0?0 ??10000

*Bryconops melanurus*

1000010000 0010000001 1011010011 0001000100 000111010- 0110000000

11201000z0 0111010000 1010000011 0-0-100010 0000000210 0000100000

0000001100 00100-1111 0100001100 1z00100101 0011000000 0000001000

0000110010 0010100010 0100000111 1000001100 0111000001 1001110100

0111001000 0010000100 01000z0000 0000010001 0001100001 0000000110

0000111100 0101101010 011??????? ??00011100 0000000010 0?01???0??

?-00000000 000000?000 0010000

*Carlana eigenmanni*

1011010000 0010000z00 1011010000 000z000101 000101010- 1101-00000

00000011-- 1010000000 0010000110 1?0-110010 000000z?1z 1000100000

0000001100 0000----11 11000011zz 1111z00100 0001000000 00000011zz

00001z0010 0010100110 0100000101 0000001101 1111000000 100101z100

01101z1110 0000000000 00000?0001 0?00010001 0000101001 001z010110

0000111100 010z101000 0111100000 000001???? 0000000010 0??11??0??

?-00000000 000?00?000 ???????

*Carlastyanax aurocaudatus*

1100010000 001010010- 1000-10000 0010000101 000101010- 1101-00---

-0101011-- 10-000111- 0010000-10 0-0-100010 0111010010 0100100000

0010100100 1011-00010 0000001110 1000101000 0000z00000 0101001100

0010100-10 0010100110 0100000100 1001001100 0111000000 0001110100

01101z1110 0000000000 00z000z00z 000z010000 0000z00000 0010000010

0000111000 01z0101000 0z0------- ---0011100 0000000011 00?1100000

0-00000000 000?000000 ???????

*Carnegiella strigata*

100000-?00 000-11011- 1-00-10001 0000000100 01011---0- 1001-01---

-1-00-11-- -0-----000 0--00-0--- 100-111010 0000000010 0000100000

0000000100 0000----11 0110001000 0000100000 0010101000 0000001000

1-00100-1z 0011000000 0100000101 0001001000 111100-000 100000-100

010010--01 00--001100 -0?00-111- -001010000 0110110000 0010000000

0201011z00 010010z000 00110000-- -000011001 0100-00010 00z1110000

0-00000000 000?001001 ??11000

*Chalceus macrolepidotus*

1010010000 001000010- 1000-10z11 0001000100 000111010- 1110000000

0000100000 0111110000 0100000011 0-0-010010 0000000-10 0000100000

0010101100 1011-1-011 0100001111 1000100101 000?001000 0000001000

0000100010 0011100000 0100000111 1000001100 0110000000 1000111100

0010001000 0000000100 0100000001 0000010001 1011110001 1000000111

0010000000 0111100000 000------- ---0001001 0100100010 0011100000

0-00000000 000?00?0?0 ??111z0

*Characidium borellii*

0---10--00 000-00011- --11010011 00000--100 0001110110 0110000100

0-?01000-0 -01-00-000 0000--1000 0-0-110010 0000---011 00000-0000

0010001100 0000------ ----0-0000 --00100000 0000100010 0000001001

0000100010 1011100000 0110000111 0000001001 111100-010 100000-100

0010001000 0011000?00 1010010001 0100000001 1000100001 0001000100

0210000000 0100101000 0010100000 0-00011100 0000000010 0?-1111100

0-00000000 000?001100 ??11000

*Characidium rachovii*

0---10--00 000-00011- --11010011 00000--100 0001110110 0-10000100

00?01-00-- -01----000 0-00--11-- 100-1-0010 0000---011 00000-0000

00z0001100 0000------ ----0-0000 --00000000 0000100010 0000001001

010010001? 1011100000 0100000111 0000001001 111100-010 10000z-100

0000101000 0010000?00 10100?0001 0100000001 1000100001 1101000100

0210000000 010-101000 000------- ---0011100 0000000010 00-1111101

0-00000000 000?00110z ??11000

*Charax stenopterus*

1000010000 0010000001 1zz0010000 0000010111 000001010- 1001-00000

0020100z10 1010000001 0010000-10 1?0-110010 0000000-10 0000z00000

1000001000 01z0------ -110001111 0000110000 0001000000 0000001111

1-00110-11 0010100100 0101010100 1000001100 0111000101 1011011100

0110111110 1100000000 0000000001 0000010001 0000001001 0010000110

1001111110 0100101000 0111100000 0?00010--- 0-10000010 0111110001

1000000000 000?000000 ???????

*Cheirodon interruptus*

101001-000 001010010- 1010010000 000z000101 000111010- 1101-00000

0010000010 1010000000 0-10000110 100-110010 0000--1011 0000100000

0001001110 0000----11 1100001000 1100000100 0000100000 0z00001101

0100100-10 10101z0111 0100000101 0000001101 1110000000 1001010100

0110111010 0000000000 00000z0001 0000010000 0000100001 0011000110

000011z000 0100101111 0111100011 0000011100 0000000010 00?1110001

0000000000 0000001010 0011100

*Coptobrycon bilineatus*

100001001- 0010100000 1000-10000 z000000101 000111010- 1101-00000

0000?000-- 1010----1- 00?0000110 1?0-1--010 0000---010 0000100000

00z0001100 00100-1011 1000000000 --00000100 0000100000 0z00001100

0100100-10 0010100110 0100000100 000000110z 111z000010 1001010100

01z01z10-0 0000000001 0000000001 0000010000 0000100001 0011000010

0000z10000 110z101000 010------- ---0011100 0000z00010 00?11??0??

?-00?00010 000?0001?1 ???????

*Creagrutus anary*

1100011000 0010100000 1010010000 0011000101 000111010- 1101-00000

0010000000 1010001000 0010000110 0-10100011 1111010010 0100100000

0110110100 1011-10010 00000011z0 1000000000 0000100000 0101001100

001110z010 0010100110 0100000100 z000001000 0110000010 100101z100

0110100010 0011000000 0010010000 0000010001 0000101000 0000000010

00000z0000 0100101010 01110000-- -000011100 0000000011 1001000000

0-00000000 000?00?0?0 ???????

*Creagrutus atrisignum*

110001z000 0010100z01 10z0010000 0010000z01 00011101z1 1101-00000

0110000000 101000z000 0010000110 0-10100011 1111010011 0100100000

0110110100 1011-10010 00000011z0 1000000000 0000000000 0101001100

0011100010 0000100110 0100000100 0000001000 0111000000 0001010100

0110101110 0011000000 0010z10000 0000010001 0000100000 0000000010

0000010000 0100101000 0111100000 0000011100 0000000011 1001???000

0-00010000 000?00?0?0 ???????

*Creagrutus cracentis*

1000011000 0010100001 100--10000 0010000001 000111010- 1101-00000

0010000000 1010001000 0110000110 0-10100010 111100001? 0100??0000

01??1?1100 00101-1011 0000001111 100010?000 0000000000 0101001100

0011100010 0010100110 0100000100 0001001000 1110000010 1001010100

011010?010 0011000000 0010010000 -000010001 0000100000 0000000010

0000000000 0100101000 0?11100000 00?0011100 000000001? ?0?1?0?000

0-00000000 000?00?0?0 ???????

*Creagrutus gephyrus*

1000010000 001010010- 100--10000 0010000001 000111010- 1101-00000

0000000000 1010001100 0010000110 0-10100011 1111000010 0100100000

0110101100 0011--0011 0000001111 100010?000 0000000000 0100001101

0111100010 0010100110 0100000110 1001001000 0110000000 1001010100

011010?010 0011000000 0010110000 0000010001 0000000000 0000000010

0000010000 0100101010 0?11100011 00?0011100 0000000011 10?111?000

0-00000000 000?00?0?0 ???????

*Creagrutus meridionalis*

1100011000 0000100001 1010010000 0010000z01 0001010111 1101-00000

0110100000 1010000000 0010000110 0-10100011 1111010010 0100100000

0110110100 1011-10010 0000001100 1000000000 0000000000 0101001100

0011100010 0010100110 0100000100 0001001000 0111000010 1001010100

0110101010 0011000000 0010z10000 0000010001 0000001000 0000000010

0000z10000 01001010z0 0111100010 0000011100 0000000011 1001???000

0-00000000 000000?0?0 ???????

*Creagrutus muelleri*

1100010000 001010010- 100--10000 0010000101 000111010- 1101-00000

?100000000 1011001000 0010?00110 0-10100011 1111?00010 0100100000

0110100100 1011-00011 0000001100 100000?000 0000010000 0101001100

0111100010 0000100110 0100000111 0001001000 0111000010 1001010100

011010?010 0010000000 0010010000 0000010001 0000000000 0000000010

00000z0000 0100101010 0?0------- ---0011100 0000000011 10?110?000

0-00000000 000?00?0?0 ???????

*Creagrutus peruanus*

1100010000 0010100000 100--10000 0010000101 000111010- 1101-00000

0100110000 1010001100 0010000110 0-10100011 1111?00010 0100100000

0100110100 1011-00011 00000011z0 100000?000 0000010000 0101001100

0111100010 0000100110 0100000101 0001001000 0111000010 1001010100

011010?010 0010000000 0010011000 0000010001 0000100000 0000000010

00000z0000 0100101010 0?0------- ---0011100 0000000011 00?110?000

0-00000000 000?00?0?0 ???????

*Creagrutus taphorni*

1100010000 0010100000 1010010000 00?0000101 000111010- 1101-00000

0010100000 1010001000 0010000110 0-??100011 1111010010 0100100000

0110110100 1011-10011 00000011z0 1000000000 000?100000 ?101001100

001110?010 00001?0110 010000010? 1001001000 ?111000010 1001010100

01101?1010 0011000000 0010110000 0?00010001 000010z000 0010000010

00000z0000 0100101010 0111100011 0000011100 0000000011 10?1110000

0-00000000 000??0?0?0 ???????

*Cyanocharax alburnus*

1000011000 0010100000 1010010000 001z000101 000111010- 1101-00000

0010000000 1010000000 0011--0110 0-10100010 0000000010 0000100000

00z0011100 00100-1011 0z000011z0 1000100000 0000100000 0100001100

0100100010 0010100110 01z0000101 000000110z 1110000000 1001110100

0110101010 0010000000 0000000000 0000010000 0000100000 0010000010

0000111000 0100101000 0111100000 0000011100 0000000010 0001110000

0-00000000 000000?010 0??????

*Cyanocharax obi*

1000011000 0010100000 10z0010000 0010000101 000111010- 1101-00000

00100000z0 z010000000 0011--0110 1?0-100010 0000000010 0000100000

00z0011100 00100-1011 0z000011z0 z000100000 00001z0000 0100001100

0000100010 0010100110 0100000101 000000110z 1110000000 1001010100

0110101010 00--000000 0000z1100z z000010000 0000z00100 0010000010

0001111z00 0100101000 0111100011 0000011100 0000000010 0001110000

0-00000000 000?001000 ???????

*Cynopotamus argenteus*

1000010000 1010000001 1100-10100 0000011101 0011010z0- 0001-00000

0020100001 0010010001 0010010110 0-11100010 0000000-10 0000100001

0000001000 01??------ ----001111 0000100000 0000000000 1000001111

1-00111011 0000100100 1101010101 1000001100 111z01-101 1011011100

0111111110 z100000000 0000000001 0000010001 000000z001 0010000110

1001111110 0100101010 0111?????? ??02010--- 0000010010 0111110001

1100000000 000?00?0?0 ??11100

*Cyphocharax spilotus*

0---10--00 000-000001 100?-10101 0000000100 0001110111 1110000100

0-000z0010 001z000000 ?000000000 0-0-110010 000----001 00000-0000

0010001--- 0--------- ------0000 ---------- ---1100000 0000000-00

0100000010 1011110010 0100000001 1001110111 11??00-010 100000-100

0000101000 0010000100 1000000001 0000000001 1001101001 1001000111

0010000000 0111101000 000------- ---0000--- 000000000- -1-?00?000

0-00000000 0000?2?0?0 ??11110

*Deuterodon iguape*

1000010000 0010000z00 1011010010 1001000101 0001z1010- 1101-00000

0000100000 101z000000 0010000110 0-10100010 0000010010 0000100000

0010001100 00100-1111 01000011z0 1100100100 0000000000 0z00001100

0000100010 0010100110 0100000101 z000001100 0110000000 1001z10100

0110101010 00z0000000 0000000001 0000010001 0000z01001 0001000110

0000111000 0101101000 010------- ---0011100 0000000010 0001110000

0-00000000 000?00?0?0 ??11000

*Deuterodon langei*

1000010000 001000010- 10110100?0 1001000101 000101010- 1101-00000

0000100000 1011000000 0010000110 0-10100010 0000011010 0000100000

0010001100 00100-1111 0100001110 1100100100 000?000000 z000001110

0000100010 0010100110 0100000101 0000001100 0110000001 1001110100

0110101010 0000000000 00001-0001 0?00010001 0000001001 0001000110

0000111000 0101101000 010------- ---0011100 0000000010 0?01???0??

?-00000000 000?0000?0 ???????

*Diapoma speculiferum*

1000011000 0010100000 1010010000 0010000101 000111010- 1101-00000

0010000000 1010000000 0011--0110 1?0-100010 0000000010 0000100000

0010011100 00100-1011 0000001110 1000100000 0000100000 0100001100

0100100010 00101z0110 0100000101 0000001100 0110000000 1001z10100

0110100010 00--000000 0000001000 0000010000 00001z0100 0010000z10

0001111100 0100101000 01111000?? 0000011100 000000-110 0001110000

0-00000000 000100?010 11?????

*Diapoma terofali*

1000011000 0010100001 1010010000 0010000101 000111010? 1101?00000

0010000000 101000??00 001z??0110 1?0?100010 0000000010 0000100000

0010011100 00100?1011 0z000011z0 1100100000 0000100000 0100001100

0100100010 0010100110 0100000101 0000001100 11z0000000 1001110100

01101z1010 00??000000 00001?1000 0000010000 0000100000 0010000010

0001111100 0100101000 011110000? 0000011100 000000?110 0001110000

0?00000000 0001000010 11?????

*Distichodus maculatus*

0---10--00 0?1-00010- 100?-13101 00?z000100 0001110110 0110000100

0-00000010 00100----- 0000000000 0-0-01?010 00-----000 000-1-0000

0-000?---0 0010------ -----00000 --0011--10 0001100010 ?000000-00

0010001010 0001100000 0100000111 1000001101 1100000010 10000z-000

001100??0- 0010000100 100000000- -?1-000001 1011111001 1101000101

0010100000 0100011000 000------- ---1000--- 0000001010 01-0011000

0-00000000 000??010?0 ???????

*Engraulisoma taeniatum*

100000-000 000-z00z00 1000-10101 00?0000100 000111010- 0-10001001

0-000011-- 0000000000 10z--00-11 0-0-101010 0010---010 0000100000

0000001100 0010?-1011 0000000000 --00100000 0010100000 0100001000

0100100010 0010100000 0000000101 1001001001 111100?0?? 11--10-100

0z1011---1 0010000000 000001011- -001011000 0000100100 0010000010

0000011000 0000100000 00???????? ???0011001 0100000011 00?1110000

0-00000000 000?0010?0 ???????

*Exodon paradoxus*

1000010000 001000010- 1011010000 000z000101 000101010- 1101-00001

0020100000 0011010000 0010000110 0-10100010 0000000-10 0000100000

0000001001 00??------ -10-001111 0000100010 00-0000000 1000001110

1-00110010 0010100110 0100000100 z000001100 1111000001 1001010100

0100101010 0000000000 0000000001 0000010001 0000z01001 0001000110

0000111000 0101101000 010------- ---0001100 0000000010 0001111000

0-00000000 000?00?0?0 ??11100

*Galeocharax humeralis*

1000010001 101000010- 1z00-10110 0000010101 000101010- 0001-00100

0020100001 0010010001 0010z10110 0-11100010 0000000-10 0000100001

0000001000 01??------ ----001111 0000100010 0000000000 1000001111

1-0011z011 0000100100 1101010100 1000001100 111z01-101 101z011100

011z111110 1z00000000 0000000001 0000010001 0000001001 0010000110

0001111110 0101101010 0111100000 0002010--- 0000010010 0111110001

1100000000 000?0010?0 ??11100

*Grundulus bogotensis*

1000011000 0?10100000 100?-10000 0000000101 00011111?? 1101-00000

00--1000-- 101-0--01- 00?--?0--- 100-110010 0000000-10 0000100000

00z0001000 0000------ -1100?1111 00001z0000 000?100000 ?000001101

0100100010 0010100110 0100000101 0000001101 111z000010 10010101?0

0zz0101010 0000000001 000000000z 0000010000 0000110001 0011000110

0000110000 0100101000 010------- ---0011100 0-10000010 0??1???0??

?-00000000 000??001?1 ??11000

*Gymnocharacinus bergii*

1000011000 00-0100000 1000-10000 0000001101 000111010- 1z01-00010

00--1-00-- 1010001000 ?0?0000110 0---110010 0000000010 0000100000

0010001110 00100-1111 1000001100 1100000000 0000000000 0000001100

0100100010 0010100110 0100000100 1000001101 1110000010 1001010100

0010101010 0000000001 0010000000 1000010000 0000110101 0011000110

0000110000 01001010z0 010------- ---001???? 0-10-00011 00?1110000

0-00000000 000?00?001 ???????

*Gymnocorymbus ternetzi*

1000010000 0010000001 1011010010 z001000101 001001010- 1101-00000

0000100010 1010000000 0010000110 0-10100010 0000--0010 0000100000

0000001100 00100-1011 0100001000 1000z00000 001?000000 0000001100

0100100010 0010100110 0100000101 0000001100 0110000001 1001010100

0100101010 0000000000 0000000001 0000010000 0000001001 0011000110

1100111110 0z00101000 0111?????? ???0011100 0-10z11010 0001110000

0-00001000 000??0z0?0 ??11000

*Hasemania nana*

1000010000 0010000000 1011010000 0001000101 00011z010- 1101-00000

00000001-- 1010000000 0010000-10 100-100010 0000--0010 0000100000

0000001100 00100-1011 010000z000 --00100000 0001000000 0000001101

0100100-10 0010100110 0100000101 0000001100 011z000000 1001010100

0100110010 0000000000 0000000001 0000010000 0000100001 0011000110

0000110000 1100101000 010------- ---0011100 0000000010 00?1110000

0-00000000 000?00?0?z ??11000

*Hemibrycon dariensis*

1000011000 0010100000 1010010000 000z000101 0001110z0- 1101-00000

0010000000 1010000000 0011--0110 0-10100010 0000000010 0000100000

0010011100 0010z-1z11 000000111z 1100100000 0010010000 0000001100

0000100010 0010100110 0100000101 0000001100 0111000000 1001010100

0110101010 0010000000 000000000z 0000010001 0000100000 0010000010

0000111100 0100101000 011??????? ???0011100 0000000010 0001110000

0-00000000 000000?0?0 0??????

*Hemibrycon surinamensis*

1000011000 0010100000 1010010000 0000000101 000111010- 1101-00000

0010000000 ?01???0000 0011--0110 0-??100010 0000000-10 0000100000

0010011100 0010z-1011 0z00001111 1000100000 0010010000 0z00001100

0000100010 0010100110 0100000101 0000001100 011z000000 1001010100

011010??10 0010000000 000000000z 0?00010001 0000100000 0010000010

0000111100 0100101000 011????0?? ??00011100 0000000010 0001110000

0-00000000 000000?010 0??????

*Hemigrammus bleheri*

1000010000 001000010- 1011010010 100z000101 000111010- 1101-00000

0000100010 1010000000 0010000110 1?0-110010 0000--0010 0000100000

0010001100 00100-1011 110000z000 1000000100 000z000000 0000001100

0100100010 0010100110 0100000101 0000001100 011z000000 1001010100

0100101010 0010000000 0000000001 0000010001 z000000001 00z1000110

0000110000 0100101000 01110000-- -010011100 0000001010 0001110000

0-00000000 000?00?0?0 ??11000

*Hemigrammus erythrozonus*

1000010000 0010000001 1011010000 1001000101 000111010- 1101-00000

00001011-- 101000-000 00100?0110 1?0-110010 0000000010 0000100000

0010001100 00100-1011 11000011z0 1100100000 0001000000 0000001100

0100100010 0010100110 0100000101 0000001100 ?111000000 1001010100

0100110010 0010000000 0000010001 000001000z 0000000001 0011000110

000011z000 0100101000 01111010z0 10000?1100 0000z0z010 00?1?10010

0-00000000 00000000?0 001zz00

*Hemigrammus ulreyi*

1000010000 0010000001 1011010010 1001000101 000101010- 1101-00000

0000100010 1010000000 0010000110 1?10100010 0000000010 0000100000

0000001100 00100-1011 01000011z0 1000100000 001z000000 0000001100

0100100010 0010100110 0100000101 0000001100 011z000000 1001010100

01001z1010 0010000000 0000000001 0000010001 000000z001 0011000110

0000111000 0100101000 011110000z 1000011100 0000z01010 0001110000

0-000?0z00 000?00?0?0 ??11100

*Hemigrammus unilineatus*

1000010000 0010000z0z 1011010000 1001000101 000101010- 1101-00000

0000100010 1010000000 0010000110 100-100010 0000000010 0000100000

0000001100 00100-1011 0100001110 1z00100000 00zz000000 0z00001100

010010?010 0010100110 0100000101 z000001100 011z000000 1001010100

01z0111010 00z0000000 00000?0001 0000010001 0000100001 0011000110

0000111z00 0z00101000 0111100000 0000011100 0000z01010 00011??0??

?-00000100 000?000000 ??11100

*Hemiodus cf. thayeria*

100-10--00 001-000001 1011010011 0000000100 0001110111 0110000100

0000010000 0011110000 1100000010 0-0-110010 000000z001 00000-0000

0010001100 0000------ ----1-1110 110-----10 ---0100000 0000001000

0100100010 0001100010 0100000011 1000001101 1100000010 1000111100

0011001000 0010000100 1000000001 0000000001 1001110001 0000000111

0010000000 0111011000 000------- ---0001000 000000000- -?-0011000

0-00000002 000?0010?0 ??11110

*Heterocharax macrolepis*

1000010000 0010000001 1011010010 z001000111 0001010110 1100000001

0000000010 0?00010000 10-0110-10 0-11110010 0000000-10 0000100000

0000001000 00??------ ----001111 0000?00010 00-?000000 0000001z10

0000110001 000110z0z0 0100000111 1000001100 0110000001 1001110110

0100101010 0010000100 01000z0000 0?0001?001 0001101001 0010000110

00001111z0 0100z01000 01110000-- -?00011100 0000000010 00?1110010

0-00000010 000?001000 ???????

*Hollandichthys multifasciatus*

1000010000 0z10000000 100?-10z00 000z0z0101 000101010- 1101-00000

01201011-- 1010000000 0010000110 100-100010 0000000-10 0000100000

00z0001100 00100-1011 0100001111 1000100000 0001000000 000000111z

1-00110-10 0010100110 0100000100 1000001100 011z000z01 100101z100

0110101110 0000000000 0000000001 0100010100 0000101001 001z000110

0000111100 0100101000 0111100010 0000011100 0000000010 0?011??0??

?-00000000 0001000000 1111000

*Hoplias cf. malabaricus*

100000--01 0?0-00011- 0-0?-10111 00--0--100 010111010- 0111-1---0

0100000000 ---000-000 1000000000 0-0-100010 ???0000-10 0000100000

0000001000 00??------ ----001111 0000?00010 0000000011 000000??-0

1-00110-11 00011-0000 1110--0111 1000001101 1100?01101 101000-100

001z001100 0000000?11 -0101-000? 0001000001 0000100001 1100000100

0210000000 0000000000 000------- ---0001001 0100000010 0001101-00

0-00000000 00000010?1 0?00000

*Hoplocharax goethei*

1000010000 0010000z01 101101001z z001000111 0001010110 1101-00000

00000011-- 10000--000 10-00?0-10 1?10110010 0000000-10 0000100000

0000001000 00??------ ----001111 0000?00010 00-?000000 0000001z00

0000110001 0001100zz0 0100000100 1000001101 111z000000 1001z10110

0100111010 0z10000100 0000010000 0?00010000 0001100000 0010000010

0000111z00 0100101001 01110000-- -??0011100 0000000010 00?1110010

0-00000010 000?001000 ???????

*Hyphessobrycon anisitsi*

1000010000 0010000z00 1010010000 z001000101 000101010- 1101-00000

0000100010 0010000000 0010000110 100-100010 0000000110 0000100000

00z0011100 00100-1011 0100001z00 1z00000100 0000000000 0000001100

0z00100010 0010100110 0100000101 0000001100 011z000000 1001010100

0100101010 0000000000 00000?0001 0000010001 0000101001 0011000110

0000111z00 0100101000 0111100000 0000011100 0000000010 0001110000

0-00000000 000?000000 ??11000

*Hyphessobrycon bifasciatus*

1000010000 0010000000 10z0010000 z001000101 000101010- 1101-00000

0000100010 1010000000 0010000110 100-110010 0000--0110 0000100000

0000001100 00100-1111 0100001z00 1100000100 0000000000 0000001100

0100100010 0010100110 0100000101 0000001100 011z000000 1001010100

01101z1z10 0000000000 0000000001 0000010001 0000z01001 0011000110

0000111100 0100101000 0111100000 0000011100 0000000010 0001110000

0-00001000 0000000000 ??11000

*Hyphessobrycon elachys*

100001001- 0010000000 1011010000 z001000101 000111z10- 1101-00000

00001000-? 101-0--01- 0-10000-10 100-1--010 0000---010 0000100000

0000001100 00100-1011 0100000000 --00z00100 0001000000 0000001100

0100100010 0010100110 0100000101 0000001101 1110000010 1001010100

0100110010 0010000000 0000000001 0000010000 0000z00001 0010010110

000011z000 0100101000 010------- ---0011100 0000100010 00?10100?0

0-00100000 000?00?0?0 ???????

*Hyphessobrycon eques*

1000010000 0010000001 1011010000 1001000101 000101010- 1101-00000

0001000z10 1010000000 0z10000110 100-110010 0000000010 0000100000

0000001100 00100-1011 01000011z0 1z00100000 000z000000 0000001101

0100100010 0010100110 0100000101 0000001100 0110000001 1001010100

0110111z10 0000000000 0000000001 0000010001 0000000001 0011000110

0z00111z00 0z00101000 0111100000 0200011100 0000000010 0001110001

0000000100 000?0000?0 ??11100

*Hyphessobrycon herbertaxelrodi*

1000010000 0010000000 1011010000 1001000101 000111010- 1101-00000

0000100010 1010000000 0010000110 100-1z0010 0000000010 0000100000

0000001100 00100-1011 0100001110 1000100000 0010000000 0z00001100

0100100010 0010100110 0100000101 z000001100 0111000010 1001010100

01z011z010 0010000000 0000z10001 0000010001 000000z001 z01z000110

0000111000 0100101000 0111100000 1000011100 0000z00010 00?1?10000

0-00000000 000?000000 ??11100

*Hyphessobrycon megalopterus*

1000010000 0010000001 1011000000 0001000101 000101z10- 1101-000-0

00110011-- 101-----1- 0110000110 100-110010 000000--10 0000100000

0000001100 00z00----- -110001111 1000110000 0001000000 000000110z

0100100-10 0010100110 0100000101 0000001100 0110000z01 100101z100

0110111z10 0000000000 0000010001 0000010001 0000001001 0011010110

0100z11000 0100011000 010------- ---0010--- 0000z00010 0001110001

0000000100 000?000000 ??11100

*Hyphessobrycon meridionalis*

1000010000 0010000000 1010010000 0001000101 000101010- 1001-00000

00001000-- 101-0--01- 0010000-10 100-110010 0000000110 0000100000

0000001100 00100-1111 0100001100 1100z00100 0000000000 0000001100

0100100010 0010100110 0100000101 0000001100 0110000000 100101z100

0110111010 0000000000 0000000001 0000010000 000010z001 0011000110

0000111100 0100101000 01110111-- -200011100 0000000010 0001z10000

0-00000000 000?000000 ???????

*Hyphessobrycon pulchripinnis*

1000010000 001000010- 1011010000 z001000101 000111010- 1101-00000

00000011-- 001z000000 0010000110 100-1z0010 0000000010 0000100000

0000001100 00100-1011 0100001100 1100100000 0001100000 0000001100

0100100010 0010100110 0100000101 0000001100 011z000000 1001010100

01001z1010 0010000000 0000010001 0000010001 0000000001 0011000110

0000111z00 0100101000 010------- ---0011100 0000z00010 00?1?10000

0-00000100 000?0000?0 ??11000

*Hyphessobrycon socolofi*

1000010000 0010000000 1011010000 z001000101 00z1z1010- 1101-00000

0001000010 1010000000 0010000110 100-110010 0000000010 0000100000

0000001100 00100-1011 0100001110 1000100000 0001000000 0000001100

0100100010 0010100110 0100000101 0000001100 0100000000 1001010100

0100111010 0000000000 00000-0001 0000110001 0000000001 0011000110

0z00111100 0100101000 011111110z 0200011100 0000z00010 0001110001

0000000100 000?00?0?0 ???????

*Iguanodectes geisleri*

1000010000 ?01000010- 1011010011 00?10001?1 000111010- 0110000000

0-00000010 1110000000 1000000?10 0-??110010 ??00---200 0???100110

0010101100 00100-1011 0100000000 --00000100 000?10?100 ?00001100?

0110000010 0010100010 01000001?1 100z001001 11110000?0 ?001110100

0z11z?1000 0011000100 0z0000?z00 0000010001 0000110?01 0010000110

1000011000 0101101000 0111?????? ???0000--- 0000000011 -1?11100??

?-00000000 000?01?0?0 ???????

*Inpaichthys kerri*

1000011-00 0110-0010- 1010000000 0001000101 000111010- 1101-1-000

00100011-- 1-100-0000 0010000-10 100-110010 0000000010 0000100000

00z0001100 0000----1- -1z0011110 1000100000 0001100000 0000001100

0100100010 1010100110 0100000101 0000001100 0111000000 1001010100

0110111010 0000000000 0000010001 0000010000 0000100001 z01z000110

0000111000 0100101000 010------- ---0011100 0000000010 00?1010000

0-00000000 000?00?0?0 ??11100

*Jupiaba mucronata*

1000010000 0010000001 1011010010 z001000101 000101010- 1?01-00000

0000100010 1010000000 0010000110 0-10100010 0000000010 0000100000

00z0001100 00100-1111 01000011z0 1100100100 0000000000 z000001100

0000100010 0010100110 0100000101 100?00110? ?11z000000 10010101?0

0110101010 0000000000 0000000001 000-110001 000000z001 0011000110

0000111z00 0101101000 01???????? ???0011100 0000000010 0??1???0??

?-00010000 000?00?0?0 ???????

*Jupiaba scologaster*

1000010000 0010000001 1011010010 0001000101 000101010- 1101-00000

0000100010 1010000000 0010000110 0-10100010 0000000010 0000100000

0000001100 00100-1111 0100001110 1100100100 00z0000000 zz00001100

0000100010 0010100110 0100000100 1000001100 011100000z 1001010100

0110101010 0000000000 0000000001 000-110001 0000001001 0011000110

0000111000 0100101000 010------- ---0011100 0000000010 0001110000

0-00000000 000?00?0?0 ???????

*Knodus breviceps*

1000010000 001010010- 1010010000 001z000101 000111010- 1101-00000

00100000z0 1010000000 0011--0110 0-0?100010 0000000010 0000100000

0010111100 0010z-1011 0000001100 1100100000 000?100000 0100001100

0000100010 0010100110 01z0000101 0000001101 1111000000 1001010100

0110100010 0010000000 00000-0000 0000010001 0000100000 0010000010

000011z000 0100101000 01111000z0 0000011100 0000001011 0001???0??

?-00000000 000?00?0?0 0?11100

*Knodus gamma*

1000011000 0010100z0- 1010010000 z01z000101 000111010- 1101-00000

0010000000 101000z000 0011--0110 0-10100010 0000000010 0000100000

0010111100 00100-1z11 0000001100 1100100000 0000100000 0100001100

0000100010 0010100110 0100000101 0000001101 1111000000 1001010100

0110111010 0010000000 0000000000 0000010001 0000z00000 0010000010

0000111z00 0100101000 01111000z0 0000011100 0000001011 0001110000

0-00000000 000?00?0?0 ???????

*Knodus heteresthes*

1000011000 0010100z01 1010010000 0010000101 000111010- 1101-00000

0010000000 101000z000 0011--0-10 0-??110010 0000000010 0000100000

0010z11100 00100-1111 0000001100 1100100100 000?110000 0100001100

0100100010 0010100110 0100000100 1000001100 1111000010 1001010100

0110110010 0010000000 0000000000 0000010001 0000100000 0010000010

000011z000 0100101000 011110?0z0 0000?????? ???0001011 0?01???0??

?-00000000 000?0??0?0 ???????

*Knodus meridae*

1000011000 001010010- 1010010000 0010000101 000111010- 1101-00000

0010000000 101000z000 0011--0110 0-10100010 0000000010 0000100000

0010111100 00100-1z11 0000001100 1100z00z00 000z100000 0100001101

0000100010 0010100110 0100000101 0000001101 1111000000 1001010100

01101z0010 0011000000 0000??0000 0000010001 0000000000 0010000010

0000110000 0100101000 0111100011 0000011100 0000001011 0?01???0??

?-00000000 00000??000 0??????

*Knodus pectinatus*

1000010000 001010010- 1000-10000 0010000001 000111010- 1101-00000

0000000000 1010-01000 0011--0110 0-10100010 0000000010 0000100000

0000100100 00100-?011 0000001110 1000100?00 0000010000 0101001100

0000100010 0000100110 0100000100 0000001100 1110000000 1001110100

0110100010 0010000000 0010100001 0000010000 0000100000 0010000010

0000110001 01001010z0 0?0------- ---0011100 0000z00011 10?1?1?000

0-00000000 000?00?0?0 1??????

*Leporinus striatus*

100-10-001 -?0-0-1001 100?-001?1 0000000100 000111010- 0110000100

0-?0100010 001101000- 1100000000 0-0-110010 000----001 0??00-0000

0??0??0-00 00??------ ----0-0000 --00000-00 000?100000 ?000-00-00

001000001- z001100010 00000-0001 1001100101 1110001010 100000-100

0010001000 0010000?00 10001-0001 0000000001 z000101001 1001000111

0010000000 0111101000 000------- ---0001001 000000000- -1-0001000

0-00000000 000?02?1?0 ??11110

*Lonchogenys ilisha*

10000z-?00 0010000001 1011010010 0001000111 0001110110 1100000000

0000000010 0000010000 10-0010110 0-11110010 0000000-10 0000100000

0000001000 00??------ ----001111 0000000010 00-1000000 0000001101

0000110001 0001100010 0100000111 1000001100 0110000001 1001z10110

0100111010 0010000100 0000000000 0?00010001 0001101001 0010000110

00001111z0 0100101000 01110000-- -??0011100 0-10000010 01?1z10010

0-00000010 000?001000 ???????

*Markiana nigripinnis*

1000010000 1011000000 1000-10100 0001000z01 000z010z0- 1101-00000

0010000000 0011010000 1010z00z10 0-10100010 0000---210 0000100000

0010111100 00100-1011 0000000000 --00000100 0000100000 0000001100

0000101110 0010100110 0100000101 0000001100 0110000000 1001010100

0110101100 0000000000 0000010001 0000010001 0000z01001 001z000110

0z00111110 0101101000 01110000-- -000110--- 0000011011 0001100000

0-00000000 0000100000 ??11100

*Metynnis maculatus*

1000010000 000100000z 100?-10101 0001000100 001001010- 1110000000

01001011-- 0010000000 0000000010 0-0-100010 00-----210 0000100000

0110101100 00100-100- -001000000 --00?00-01 100?100000 000000??00

0100100010 01001000?0 0100000100 1000001001 1110001000 1000011100

1110111000 0000000100 00000?0001 0000010000 0001101011 1101000100

020011111? 0101001000 00???????? ???0000--- 0-11010011 10-1?010??

?-??000000 10000010?0 0?11111

*Micralestes stormsi*

100000-000 001-00010- 1000-10111 00?1000100 0001110111 1111-00100

0000010000 0010001000 11z0000011 0-0-11010z 00-----210 0000100000

0110111100 00100--111 0000000000 --00000101 0000101000 0100001000

0100100010 0011100010 0100000100 1000001100 111z000000 1000111100

0110101000 0010000100 0100010000 0000000001 0010101000 0000000111

0110010000 0111000000 100------- ---0001001 0000100011 00?1100000

0-00000000 000?00?0?0 ???????

*Microgenys minuta*

1000011000 001-100000 1000-10000 0010000101 000111010- 1101-00000

0010000000 1010001100 0010000110 0-10110010 0000010011 0000100000

0010101100 0010?-00-- -000001100 1000000000 0000100000 0100001100

0000100011 0010100110 0100000101 0000001100 1111000010 1001010100

0110101010 0010000000 00101-0000 0000010001 0000100000 00100??010

0000000000 0100001000 01???????? ???0011100 0000z00011 1001100000

0-00000000 000?0000?0 ???????

*Microschemobrycon casiquiare*

1010010000 001000010- 1010010000 0z00000101 000111010- 1101-00000

0010000000 1010000000 0011--0110 100-110010 0000000011 0000100000

0000001100 0000----1- -110011110 1000110000 0001100000 0100001100

010010z010 0010100110 0100000101 0000001100 1111000000 1001110100

01z0111010 0010000000 0000000001 0000010001 0000000001 0010000110

000011z000 0100101000 01???????? ???0011101 000000z010 0001110001

0000000000 000?00?0?0 ??00000

*Mimagoniates rheocharis*

100001??00 0010-00000 1010000000 z00z0001z1 000111010- 1?01-00000

0110000z-0 1010?0--00 0-11--0-10 10--100010 0000000010 0000100000

0000011100 00100-1011 0z00001110 1000100?00 00001z0000 0z00001100

0000100-10 0010100110 0100000101 1000001100 011z000000 1001010100

01101010-0 00--000000 00000?1000 0?00010z00 0000110001 0010000100

0001111100 0100101000 0110100111 0-10011100 000000-010 00?11??0??

?-00000000 0001000000 11?????

*Moenkhausia bonita*

1000010000 0010000001 1011010010 z001000101 000101010- 1101-00000

0000100010 1010000000 0010000110 0-10100010 0000000010 0000100000

0000001100 00100-1011 0100001z00 1000100000 0011000000 0000001100

0100100010 0010100110 0100000101 0000001100 1110000000 1001010101

0100100010 0011000000 0000000001 0000010001 000000z001 001z000110

0000111000 010z101000 010------- ---0011100 0000z01010 0001110000

0-00000000 000?0000?0 ???????

*Moenkhausia dichroura*

1000010000 0010000001 1011010010 1001000101 000101010- 1101-00000

0000100010 1010000000 0010000110 0-10100010 0000000010 0000100000

0000001100 00100-1010 010000zz00 1000100000 0010000000 0000001100

0100100010 0010100110 0100000101 0000001100 1100000000 100101z101

0110101010 0010000000 00000?0001 0000010001 0000z01001 0011000110

0000111z00 01011010z0 010------- ---0011100 0000001010 0001110000

0-00000000 00000000?0 ??11000

*Moenkhausia sanctaefilomenae*

1000010000 0010000000 1011010000 z001000101 000111010- 1101-00000

00000000z0 1010000000 0010000110 1010100010 0000000010 0000100000

00z0001100 00100-1011 0100001z00 1100z00000 001?000000 0000001100

0100100010 0010100110 0100000101 0000001100 0111000000 1001010100

0100101010 0000000000 00000?0001 0000010001 0000z00001 0011000110

0000111000 010z101000 01110000-- -?00011-11 0000101010 00?1110000

0-00000000 00100000?0 001z000

*Moenkhausia xinguensis*

1000010000 0010000001 1011010010 1001000101 0001010z0- 1101-00000

0000100010 101z000000 0z10000110 0-10100010 0000000010 0000100000

0000001100 00100-1011 0100001z00 1100100000 0010000000 0000001100

0000100010 0010100110 0100000101 0000001100 011000000z 1001z10100

0100101010 0000000000 0000000001 0000010001 0000001001 0001000110

0000111000 0101101000 01110000-- -?00011101 0000z01010 0001110000

0-00000000 0000000000 ???????

*Nantis indefessus*

1000011000 0010100000 1000?10000 0010000101 000111010? 1101?00000

00?0100z00 101000z0z0 0011??0?10 0?0?110010 00000z0010 0000100000

0010z01100 00100?1011 0100001110 1000100000 0000100000 0z00001zz0

0z00100010 0010100110 01z0000101 0000001101 1111000000 1001010100

0z101z1z10 00z0000000 0000000000 0000010001 0000100000 0010000010

00001z0000 01001z1000 011z1000z1 z??0011100 0000000010 0001110000

0?00000000 000?00z000 ???????

*Nematobrycon palmeri*

1000011000 001000010- 1011010000 100z0001z1 000101010- 1101-00000

00000011-- 1010000000 0-10000110 1-0-1z0010 0000000-10 0000100000

0010011100 00100-1011 00000?1111 1000100000 000?100000 0000001100

0100100010 0010100110 010000010? ?000001100 111z000010 1001010100

0110101010 0000000000 0000z00001 0000010001 0000100001 0010000110

0000111100 0100101000 010------- ---001???? 00000?0010 0??1???0??

?-00000000 00000????1 0011000

*Nematocharax venustus*

1000010000 0010000001 1011010000 z001000101 000101010- 1101-00000

0000100000 1010000000 0010000110 100-100010 0000000-10 0000100000

00z0001100 00100-1111 0100001111 11?0100100 0001000000 00000011z0

01001z0010 0010100110 0100000101 1000001100 0111000000 1001010100

0110101010 00z0000000 0000000001 0000010001 0000z00001 0011010110

0000111z00 0100101000 011110z00? ?00001z100 0000001010 0??11??000

0-00000000 000000?000 00?????

*Odontostilbe microcephala*

1010010000 001010010- 1010010000 0000000101 000111010- 1101-00000

0010000010 0010010000 0010000110 0-10110010 0000001011 0000100000

0001001110 0000----11 1100001100 1100z00100 0000100000 0100001101

0100100010 1010100111 0100000101 0000001101 1110000000 1001010100

0110111010 0010000000 0000000001 0000010011 0000100001 0011001110

0000111000 0100101000 0111100001 0000011100 0000000010 0001110001

0-00000000 000?000010 ??11100

*Odontostilbe paraguayensis*

1010010000 001010010- 1010010000 z000000101 000101010- 1101-00000

0010000010 0010000000 0110000110 0-10110010 0000001011 0000100000

0001001110 0000----11 1100001100 1100z00100 0000100000 010000110z

0100100010 1010100111 0100000101 0000001100 0111000000 1001010100

0100111010 0010000000 00001-0001 0000010011 0000001001 0011001110

0000111000 0100101000 0111100001 0000011100 0000z00010 0001010001

0-00000000 000000z010 0011100

*Odontostilbe pequira*

101001-000 001010010- 1010010000 0000000101 000111010- 1101-00000

0010000010 0010000000 0010000110 0-10110010 0000001010 0000100000

0001001110 0000----11 1100001100 1100z00000 000?100000 0z00001100

0100100010 z010100110 0100000101 0000001100 0110000000 1001010100

0110111010 0010000000 0000010001 0000010011 0000100001 0011001110

0000111000 0100101000 0111100001 0000011100 0000000010 0001110001

0-00000000 0000000010 0011100

*Odontostoechus lethostigmus*

1000010000 0010100z0? 1010010000 001z000101 000111010? 1101?00000

0010000000 1010001000 0011??0110 0?10110010 0000010010 0000100000

00100111z0 00z0????11 1??0001110 1100000000 0000100000 0100001100

0000100010 0010100110 0100000101 0000001101 111z000000 1001z10100

0110101010 0010000000 0000000000 0?00010001 0000100000 0010000010

000011z000 010z101000 011110001z z000011100 0000000011 00011??000

0?00000000 0000000010 0??????

*Oligosarcus bolivianus*

1000010000 001000000z 1010010000 000z10-001 000101010- 1101-00000

0020100001 0010000001 0010000110 0-10100010 0000000-10 0000100000

0010001100 01??------ ---0001111 1000100000 0010000010 1000001110

1-00111110 0000100110 0100000101 0000001100 0110000101 1001010100

0110101110 0000000000 0000010001 0000010001 0000100001 0011000110

0000111000 0101101000 0111100011 0000011100 0000000010 0001110000

0-00000000 000?001000 ???????

*Oligosarcus itau*

1000010000 0010000000 1010010000 0001000001 000101010- 1101-00000

0020100000 0010000000 0010000010 0-10100010 0000000-10 0000100000

0010001100 00100-1011 0100001111 1000100000 0000000010 1000001100

000011z110 0000100110 0100000101 0000001100 0110000001 100101z100

0110101110 0000000000 0000000001 0000010001 0000101001 0011000110

0000111000 0101101000 0111100000 0000011100 0000000010 0001110000

0-00000000 000?00?000 ???????

*Oligosarcus jenynsii*

1000010000 0010000z0z 1010010000 000110-001 000101010- 1z01-00000

0020100001 0010000001 0010000110 0-10100010 0000000-10 0000100000

0010001100 01??------ ---0001111 1000100000 0010000010 1000001110

1-00111110 0010100110 0100000101 0000001100 0110000101 1001010100

0110101110 0000000000 0000000001 0000010001 0000100001 001z000110

0000111z00 0101101000 01111000z1 0000011100 0000000010 0001110000

0-00000000 000?001000 ??11000

*Oligosarcus longirostris*

1000010000 001000010- 1010010010 000110-001 000101010- 1001-00000

0020100001 z010000001 0010000110 0-10100010 0000000-10 0000100000

0010001z00 01??------ ----001111 1000100000 0010000010 1000001101

1-00111110 00z0100110 0100000101 1000001100 0110000101 0011010100

0110101110 0000000000 0000000000 0000010001 0000100001 001z00?110

0000111z00 0101101000 01???????? ???0011100 0000000010 0001110000

0-00000000 000?000000 ??11000

*Oligosarcus menezesi*

1000010000 001000010- 1010010000 000110-001 000101010- z001-00000

0020100001 z010000001 0010000110 0-10100010 0000000-10 0000100000

0010001100 01100--011 100-001111 1000100000 0010000010 1000001110

1-00111110 00z0100110 0100000101 0000001100 0110000001 1011010100

0110101110 0000000000 0000000001 000z010001 0000100001 001z00?110

0000111z00 0101101000 01???????? ???001z100 0000000010 0001110000

0-00000000 000?000000 ???????

*Oligosarcus pintoi*

1000010000 0010000001 1010010000 000110-101 000101010- 1101-00000

002010000z 0010000001 0010000110 0-10100010 0000000-10 0000100000

00z0001100 01??---011 1000001111 1000100000 00z0000010 0000001100

1-00111110 00z0100110 0100000101 1000001100 0110000001 1001010100

0110101110 0000000000 0000000001 00000100?1 0000100001 001100?110

0000111100 0100101000 01???????? ???0011100 0000000010 0001110000

0-00000000 000?0010?0 ??11000

*Paleotetra entrecorregos*

?????????? ???????00? ?01?0??0?0 ???1??0??? ??010??0?? ???1-0001?

0000100000 ?0?100000? ?0???????? ????1??01? ????000?10 ????100000

0??10??100 00100-?011 10000?111? ?????0??00 00???????? ??????11??

????100?1? 001?????00 0100000??? ?????????? ?????????? ???0???1?0

0110?????? 0010??0?00 0?????000? 0?0??1???0 0?001000?1 00100??110

0?0011100? 010?101?0? 01???????? ???0?????? ???0-????? ??????????

?????????? ?????????? ???????

*Paleotetra aiuruoca*

10??0????? ?????????? ???????0?? ??????0??? ??010????? ???1-0?0??

0000100000 ??????0?0? ?0???????? ????1??01? ????000?10 ????100000

???1???100 00100-?011 10000?1110 ?????0??00 00???????? ?????????0

????100?1? 0????????? ??0??????? ?????????? ?????????? ??????????

0110?????? 0010??0?0? 0?????000? 0?0??1???0 0?001000?1 00100??110

0?0011100? ?10?101?0? 01???????? ???0?????? ???0-????? ??????????

?????????? ?????????? ???????

*Paracheirodon axelrodi*

1010010000 0010000001 1011010000 1001000101 000111110- 1101-00000

00001000-- 101000-000 0010000-00 100-110010 0000--1010 0000100000

0000001100 0000----11 110000z000 1100000100 0001100000 0000001100

0100100-10 00101z0110 0100000101 1000001100 0110000000 1001010100

010010z010 0010000000 0000000001 0000010001 0000z00001 0010000110

000001z000 0100101000 0110100000 0--0011100 0000z00010 0??1?10001

1?00100000 000000?0?0 ??11100

*Paragoniates alburnus*

10000??100 001011010- 100?-00100 00?00001?1 ?00101010- 1101-0??1?

001?000000 ?010001100 0?10000110 101011?010 ??00000-10 000?100000

0000001100 00z0?--?1- -1000?1111 1000100000 000??0??00 ?000?01100

0100100010 001010?110 0100000101 0?00001100 ??10000000 1001010100

01101???00 0010001000 00001-0zz? ??0001?001 0000110001 0010000110

0101111110 0100101000 010------- ---001???? 0000010010 00???100??

?-00000000 000000?0?0 00?????

*Parecbasis cyclolepis*

1010010000 001000010- 1011010000 000z000101 000101010- 1101-00000

0010000000 0010000000 01100?0110 0-??110010 0000---011 0000100000

0001001100 0000----1- -110010000 --00010000 000?000000 0000001100

0100100010 0010100110 0100000101 0000001101 1100000000 1001110101

0110101010 0010000000 0000000001 0000010001 0000001001 000z000110

0000111000 0z00101000 01???????? ???0011100 0000001010 00?11100??

?-00000000 000??0?0?0 ???????

*Parodon nasus*

0---10--00 000-10111- 1-0?-11101 0000010100 0101110011 0110100100

0-?0000000 0010000000 11000-0000 0-0-110010 ??0-010001 0??00-1000

00100011-0 00??------ ----1-1100 110-0----- ---1100000 0000001000

000010?011 0001100000 0100000001 1001001101 110010-010 100000-100

0010000000 0011000100 00000z0001 0100000001 000z1z1001 1001000111

0010000000 0101101000 000------- ---0001001 010000000- -?-0011000

0-00000000 000??110?0 ??11110

*Phenacogaster tegatus*

1000010000 0010000001 1010010000 0000000111 000101010- 1101-00000

0020100010 1010000000 0010000110 100-110010 0000000-10 0000100000

0000001100 00100-1011 0110001111 0000100000 0001000000 0z00001100

0100100011 0010100110 0101000101 0000001100 0111000101 1001011100

0110111010 0000000000 0000000001 0000010001 0000101001 0010000110

0001111110 0100101000 01110100-- -000011100 0000000010 0001110001

1000000000 000?00?010 ???????

*Phenagoniates macrolepis*

1000010-00 0?10110z00 1000-00100 00?00011z1 100101010- 1101-00010

0010000000 1010000100 00100?0-10 1010101010 0000110-10 000?100100

0000001100 0000----11 11000?1111 1000100000 000?100000 0000001100

0110100010 0010100110 0100000101 0000001100 0111000000 1001010100

01111?1000 0010000000 00001-000? ??0001?000 0000110000 0010000010

0101111110 0100101000 01???????? ???0000--- 00000?0011 00?11?0000

0-00000000 000?0010?1 ???????

*Piabarchus analis*

100z011000 0010100000 1010010000 0010000101 000111010- 1101-00000

0010000000 1010001000 0011--0110 0-10100010 0000000010 0000100000

0010111100 00100-1011 0000001100 1000z00000 0000100000 0100001100

0000100010 0010100110 0100000100 1000001100 1111000000 1001z10100

0110101010 0010000000 0000000000 0000010000 0000z00100 0010000010

0001111100 010z1010z0 0110100000 0--0011100 0000000011 00011??000

0-00000000 000?00?000 ???????

*Piabina argentea*

1100010000 0010100000 1010010000 0010010z01 000111010? 1101?00000

0010000100 101000z000 0011??0110 0?10100010 00100z0010 0000100000

0010111100 1011?00010 0000001100 1000000000 0000000000 0100001100

0000100010 0010100110 0100000111 0000001100 0110000010 1001010100

0110111010 0011000000 0000000000 0000010001 0000100000 0010000010

000011z000 0z00101000 011110001? 0000011100 0000000011 10?1z11000

0?00000000 0000000010 0?11100

*Piabucus melanostomus*

1000010000 0010000z00 1111010011 00?1000110 000101010- 0110000000

0-00010010 1010000000 1010000110 0-10110010 0000011200 000?100010

0010101100 0000----11 0100001100 1100100100 0000101100 0000011000

0110000010 0010100z10 0100000100 1000001001 1110000000 1001010100

0011011z00 0110001100 010001011- -001010001 001011z100 0010000010

1001111110 0101101000 01110000-- -000000--- 0000000011 -1?1010000

0-00000000 000??10000 ??11000

*Piaractus mesopotamicus*

1000010000 0001000z01 1000-10101 0001000100 000z01010- 1110000000

0100100000 0011010000 1000000010 0-10000010 00-0---210 0000110000

0110101100 00100--00- -001000000 --00000-01 0000100000 0000001000

0000100011 01001000?0 0110000100 1001001001 1100001001 1000011100

1110101100 1000000100 00000?0001 0000010000 00011z1001 1110000101

0200111000 0101001000 00???????? ???0000--- 0-11010010 00?1101000

0-00000000 100000?0?0 ??11110

*Poptella paraguayensis*

1000010000 0010000001 1011010010 1001000z01 001001010- 1101-00000

0000100010 1010000000 0010000110 0-10100010 0000000010 0000100000

0000001100 00100-1011 01000011z0 1z00000000 0010000000 0000001100

0000100010 0010100110 0100000101 z000001100 0110000001 1001010100

0100101010 0000000000 0000000001 0000010000 0000001001 0001100110

0100111100 0101101000 0111101000 0200011100 00000z1010 0001110000

0-0000z000 0000?01000 0?11000

*Prionobrama paraguayensis*

10000--100 001011010- 1010100100 0000001111 100111010- 1101-00010

00100011-- 1010001100 0010000110 1110111010 0000000-10 0000100000

0000001100 0000----1- -110011111 0000100000 0000100000 0100001100

0100100010 0010100110 0100000101 0000001100 0110000100 1001010100

0110111000 0010000000 00011-000? 0?00010000 0000100000 0010000010

0001111100 0000101000 0111100010 0000011100 0000000010 00?1010000

0-00000000 0000001010 0011100

*Pristella maxillaris*

1000010000 001000000z 1011000000 00010001z1 000111010- 1101-00000

0001000010 1010000000 0110000-10 100-1z0010 000000001z 0000100000

0000001100 00z00--011 01000011zz 0000100000 0001000000 0000001110

0100100010 00101z0110 0100000101 0000001100 z110000101 1001010100

01101z1010 0010000000 00000?0001 0000010001 0000000001 0011000110

000z111000 0z00101000 011110000z 1000011100 0000101010 0001010000

0-00000100 000?000000 ??11100

*Probolodus heterostomus*

1000010000 0010000000 1011010000 100z000101 000101010- 1101-00000

0000100010 101z000000 0010000110 0-??100010 0000000010 0000100000

0000001101 0000----1- -0000011z0 1000000000 000?000000 0000001100

0100100010 00101z0110 0100000101 z000001100 0110000000 1001010100

0110101110 0010000000 00000z0001 0000010001 0000101001 0011000110

0000111100 0101101000 0111101100 0000011100 0000000010 00011100??

?-00000000 000?00?0?0 ??11000

*Prochilodus lineatus*

0---10--00 00--000001 100?-10101 0001000100 000101010- 0110100100

0-?0100010 0011010000 1100000000 0-0-110010 ??00----0- 0??00-0000

0-00001--- 0--------- ------0000 ---------- ---1100000 00000-0-00

001000001- 1101100010 0100000001 1001110111 1100001010 100000-100

0011001100 0000000100 10000?0001 000000?001 100z101001 1001000111

0010000000 0111101000 000------- ---0001001 010000000- -0-0001000

0-00000000 00000210?0 0?11110

*Prodontocharax melanotus*

1010011000 0010z0010- 1010010000 z0?0000101 000111010- 1101-00000

0010000010 1010000000 0z10000110 0-10110010 0000010-11 0000100000

00z1001100 0000----11 1100001110 1z01100000 0001100000 0100001100

0100100010 1010110110 0100000101 1000001101 11z0000000 1001010100

0110111010 0010000000 0000000001 0000010011 0000101001 0011000110

0000110000 0100101000 01111000z0 0000011100 0000000010 00?1010001

0?00000000 000?000010 ???????

*Psellogrammus kennedyi*

1000010000 0010000000 1010010000 z001000101 000001010- 1101-00000

0000100010 1010000000 0010000110 z?10100010 0000---110 0000100000

0010111100 00100-1010 0100000000 --00000000 0000100000 0000001100

0100100010 0010100110 0100000101 0000001100 0110000000 1001010100

01101z1010 0000000000 0000000001 0000010001 0000001001 0011000110

0100111110 0100101000 01110000-- -002001100 0000000011 00011100??

?-00000000 000?100000 ??11000

*Pseudochalceus kyburzi*

1000010000 0010000z00 1011010000 0001000101 000z01010- 1101-00000

00000011-- 10-0000000 0010000110 100-100010 0000000-10 0000100000

00z0001100 00100-1011 0100001111 1000100000 00z0000000 0000001111

1-00110010 0010100110 0100000101 0000001100 0110000010 1011010100

0110101110 0000000000 0000000000 0100010001 000010z001 0011010110

0000111100 0100101000 010------- ---0011100 00000??010 0?01???0??

?-00000000 00000?00?0 00?????

*Pseudocorynopoma doriae*

1000010000 0010100z00 1000-10000 00z0000100 000111010- 1101-00000

0010000000 z01000z000 0011--0110 0-101z0010 0000000010 0000100000

00z0011100 00100-1011 01z00011z0 1z00100000 0000z00000 0000001y00

0100100010 0010110110 01z0000101 000z001100 z110000000 1001010100

01101z10-0 00--001000 0000011100 0000010000 0000110000 0010010110

01011111z0 0100101000 0111000z-- -000011100 0000000110 0001110000

0-00000000 0001000000 11?????

*Pyrrhulina australis*

100010--01 000-00011- 0-0?-10100 0000000100 010110-10- 0111-00100

0010010010 -010001000 1?000-0-00 100-110010 0000000010 0000100000

0010001000 00??------ ----001110 000011-010 0000100000 00000-???0

0100100?10 10101000z0 00000-0100 1000001101 1111000010 100000-100

00100000-0 0000000100 00?00?001- -000000001 0000110001 0010000110

0210000000 0000100000 000------- ---0001001 0100100010 0100001-00

0-00000000 000?0010?1 ??00000

*Rhaphiodon vulpinus*

1000010001 -000000001 0111100111 0000000100 0001110110 0010101000

01-0100001 0010010001 00000-0000 0-11100010 0000000-10 0000100000

0010001000 01??------ ----001111 000010-000 000?000001 1010001011

1-00111011 0011101000 1111001110 1000001101 11002-1101 101000?1?0

0011001110 0000011100 0--0--011- -001010000 0111110101 0010000111

0001111110 010100-000 010------- ---0000--- 0-z0010010 0011111000

0-01000000 000?0010?0 ??11110

*Rhoadsia altipinna*

1011010000 0010000z01 1011010000 0000000111 000z01010- 0101-00000

00000000-- 1010000000 0010000z10 100-110010 000001z01z 1000100000

0000001100 00100--011 11000011zz 1111z00100 0001000000 00000011zz

00001z0010 0010100110 0100000101 0000001100 11100000?? 1001010100

0110101110 0000000000 0000000000 0000010001 0000101001 0011010110

0000111100 0100101000 010------- ---001???? 0000000010 00?111?001

0?00000000 000??0?0?0 ???????

*Roeboexodon guyanensis*

1100?10001 0011000001 1011010000 0000100101 0001z1010- 1101-00001

?0-0000000 00100z00?0 0010000110 0-??1--010 0000000-10 000010000-

00z0001101 00??------ ---0001111 0000?0-000 000?000000 ?000001110

0100110010 0010100110 0100000100 1000001100 0111000000 1001110100

01z0101010 0010000000 0000000001 0000010001 0000100001 0010000110

0000111000 0100101000 0111?????? ???0011100 000000001? ?0?1110001

0?00000000 000??0?0?0 ???????

*Roeboides descalvadensis*

1000010000 1011000001 1110010000 0001010111 000001000- 1001-00000

00201011-- 0011010001 00100z0110 0-11100010 0000000-10 0000100000

1000001001 00??------ ----001111 0000100000 0000000000 1000001101

1-00110011 0010100100 0101010100 1000001100 011z000101 1011011100

0110111110 z100000000 0000z10001 0000010001 0000001001 001z000110

1101111110 010z101000 0111----?? ???0011100 0-10000010 0111110001

1000000000 000?000000 ??11100

*Roeboides microlepis*

1000010000 1010000001 110?-10100 0001010111 000001000- 1001-00000

00201011-- 0011010001 0010000110 0-11100010 0000000-10 0000100000

1000001001 00??------ ----001111 0000100000 0000000000 1000001111

1-00111011 0000100100 0101010100 1000001100 0110000101 1011011100

0110111110 1100000000 0000000001 0000010001 0000001001 0011000110

1101111110 01011010z0 01110000-- -000001100 0000010010 0111110001

1000010000 000?000000 ??11100

*Salminus brasiliensis*

1000010000 0?000001z- 1-0?-10zz1 00?0010000 000111000- 0z10100000

0120100000 001101000- 10000?0010 0-11100010 ??00000-10 0000100000

0000001100 00100-?01- -110001111 z000100010 000?000000 ?000001111

1-00110010 0000100010 1100000111 1000001100 0100001101 0000011100

0011001100 0000000100 0000000001 0000010001 0001110001 0011000110

0000111z00 0101101010 011?1????? ???0001100 0000000010 0001100000

0-00000000 000?0000?0 ??11000

*Serrapinnus calliurus*

1010010000 001010010- 1010010000 z000000101 000111010- 1101-00000

0010000010 0010000000 0110000110 100-110010 0000001011 0000100000

0001001110 0000----11 1100001100 1100000100 0000100000 0100001101

0100100010 1010110111 0100000101 0000001100 0111000000 1001010100

0100101010 0010000000 0000010001 0000010001 0000001001 0011000110

0000111001 0100101111 0111100011 0100011100 0000z00010 0001010001

0000000000 000000z010 0011100

*Serrasalmus maculatus*

1000010000 0001?0010- 1-00-10101 00?1010110 000001010- 1100000000

01001011-- 0010000000 1000000110 0-0-100010 0000---210 0000100000

0110100100 00??------ ----000000 --00000-00 1000100010 0000001000

0000100010 01001000?0 0100000100 1000001101 1110001001 1000011100

1110101100 1000000100 00000?0001 0000010000 0001101011 1100000101

0200111100 0101011000 000------- ---0000--- 0-11010011 100110100?

?-10000000 10000010?0 ??11111

*Stethaprion erythrops*

1000010000 0010000001 1011010010 10?1000101 001001010- 1101-00000

0000100010 0010000000 0010000110 0-10100010 0000000010 0000100000

0000001100 00100-1011 0100001z00 1000100000 001?000000 0000001100

0000100010 0010100110 0100000101 0000001100 ??10000000 ?001010100

01101?1010 0000000000 0000000001 0?01010000 0000001001 1001100110

0100111110 0101101000 01110???-- -200011100 1000011010 00?11??0??

?-00000000 000??0?000 ???????

*Stichonodon insignis*

10?0010000 ?010?0000- 10110100?0 00?1000101 001001010- 11?1-00000

0000000010 1010000000 ?110000110 0-10110010 0000---010 0000100000

0000001100 00100-1011 0100010000 --00100000 000?00?000 ?100001100

0100100010 0010100100 0100000101 0000001100 0100000?00 1001010101

01101?1?10 0000000000 0000010001 0?0001??00 0000001001 1001000110

0100111100 0101101000 011??????? ???0011101 0-10011010 0??1???0??

?-00000000 000?00?0?0 ???????

*Tetragonopterus argenteus*

1000010000 0010000001 101z010010 0001000101 000001010- 1101-00000

0000100010 1010010000 0010000110 0-11100010 0000000110 0000100000

0000001100 00100-1011 01000011z0 1000z00000 0011000000 0000001100

0000100010 0010100110 0100000101 0000001100 0110000001 1001010100

01z0101010 0000000000 0000000000 0000010001 0000001001 0011000110

01001111z0 01011010z0 0111100000 0000111101 0000z11010 0001110000

0-00001000 0000001000 0?11100

*Thayeria boehlkei*

1000010000 001000000z 1011010010 1001000101 000111010- 1101-00000

0000100010 101000001- 0010000110 100-100010 0000--0010 0000100000

-010001100 00100-1011 0100001000 1000100000 0011000000 0z00001100

0100100010 0010100110 0100000101 0000001100 011z000000 1001010100

0100100010 0010000000 0000000001 0000010001 0000000001 0011000110

0000110000 0100101000 010------- ---0011100 0000101010 00?1110000

0-00000002 000000?0?0 ??11000

*Thayeria obliqua*

1000010000 001000010- 1011010010 100z000101 000111010- 1101-00000

0000100010 1010000000 0010000110 100-100010 0000--0010 0000100000

00z0001100 00100-1011 010000z000 1000100100 0011000000 0000001100

0100100010 0010110110 0100000101 0000001100 0110000000 1001110100

0100100010 0010000000 0000100001 0000010001 0000000001 0010000110

0000110000 0100101000 010------- ---0011100 0000101010 00?11100??

??00000002 000?00?0?0 ??1z000

*Thoracocharax stellatus*

100000-?00 000-11011- 1-00-10000 0000000100 01011---0- 1001-01000

01-01-11-- -000---000 0-000-0--- --0-111010 0000000010 0000100000

0000000100 00100-1011 01100011z0 0000100000 0000101000 0000001010

1-00100010 0010000000 0110--0001 0001001000 111000-000 100000-100

010010--01 00--001?00 -0?00-111- -001010000 0110110001 1110000100

0201011110 0000101000 00110000-- -000011001 0100-10010 00z1100000

0-00000000 000?0110?0 ??11zz0

*Triportheus nematurus*

1000010000 001000010- 1010010010 0001000100 000111000- 1110101000

1000000010 0000010000 1000100010 0-11000010 0000000210 0000100000

0010101100 1011-1-010 0101001100 1000000101 0000100000 z000101000

0100101010 0011000000 0100100111 0000001100 0100001000 1000111110

0110001100 0000111100 00-001011- -001011000 001111z001 0010000110

0000111100 0101101000 010------- ---0011001 0000000010 0001110000

0-00000000 000?0010?0 ??11100

*Triportheus pantanensis*

1000010000 001000010- 1010010010 0001000100 000111000- 1110101000

1000000010 0000010000 1000100010 0-11000010 0000000210 0000100000

0010101100 1011-1-010 0101001100 1000000101 0000100000 z000101000

0100101010 0011000000 0100100111 0000001100 0100001000 1000111110

0110101100 0000111100 00-001011- -001011000 001111z001 0010000110

0000111100 0101101000 010------- ---0011001 0000000010 0001110000

0-00000000 000?0010?0 ???????

*Xenagoniates bondi*

10000--100 011011010- 1001-00100 0000001111 100101010- 1101-00?10

0010?000?0 ?0?0001100 0?10000-10 0-??1??010 0000110-?0 000?100100

00?0111100 0000----11 1100001111 1000z00000 000?100?10 0000001100

0110100010 0010100110 0100000100 ?100001100 0111000000 1001010100

011111??00 0011000000 0000000z1- -00001?000 0000110100 0010000010

0101111110 0100101000 010------- ---0011100 0000000011 1??11??0??

??00000000 000000?0?0 00?????

**References**

1. Mirande, JM*.* Phylogeny of the family Characidae (Teleostei: Characiformes): from characters to taxonomy. Neotrop Ichthyol. 2010; 8 (3): 385‒568.

2. Mirande JM, Jerep FC, Vanegas-Ríos JA. Phylogenetic relationships of the enigmatic *Carlastyanax aurocaudatus* (Eigenmann) with remarks on the phylogeny of the Stevardiinae (Teleostei: Characidae). Neotrop Ichthyol*.* 2013; 11(4): 747‒766.
