# Supplementary figures and images for: Phycocharax rasbora, a new genus and species of Brazilian tetra (Characiformes: Characidae) from Serra do Cachimbo, rio Tapajós basin
Source: PLoS One. 2017 Feb 15;12(2):e0170648. doi: 10.1371/journal.pone.0170648 (PMC5310855; doi:10.1371/journal.pone.0170648)

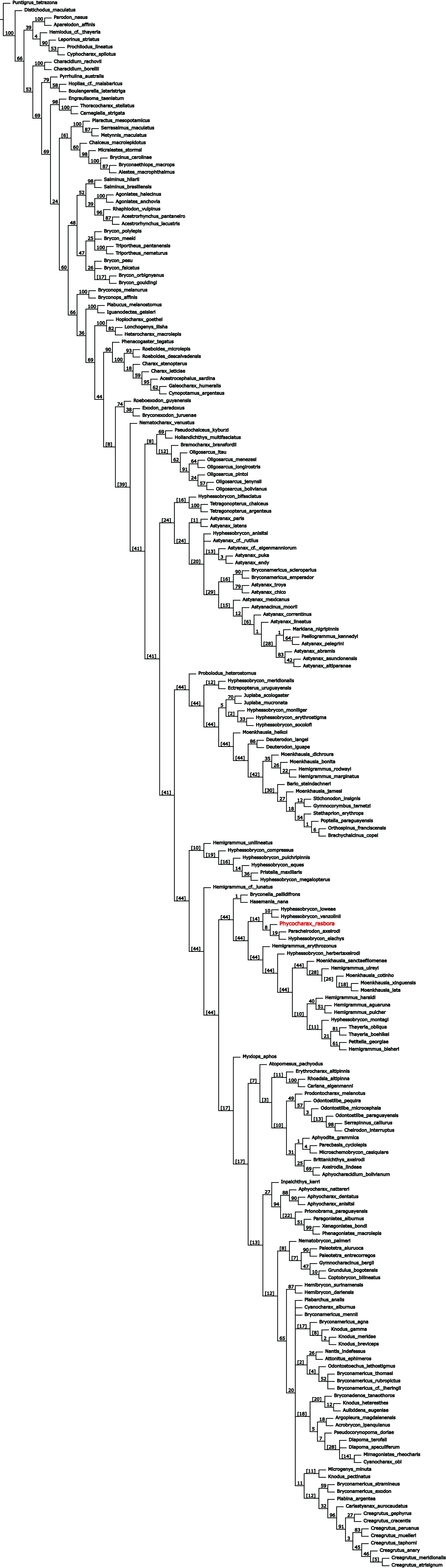

Supplement: S1 Fig — Numbers on nodes are GC values after a Symmetric Resampling. Clades with negative values (shown between brackets) are weakly supported. (TIF) [file pone.0170648.s002.tif]
