# Supplementary material for: Phycocharax rasbora, a new genus and species of Brazilian tetra (Characiformes: Characidae) from Serra do Cachimbo, rio Tapajós basin
Source: PLoS One. 2017 Feb 15;12(2):e0170648. doi: 10.1371/journal.pone.0170648 (PMC5310855; doi:10.1371/journal.pone.0170648)
Supplement: S1 Appendix — Only c&s and alcohol specimens are listed. (DOCX) [file pone.0170648.s003.docx]

**S1 Appendix**

Material Examined

*Acestrorhynchus lacustris*: MZUSP 27893, 6, 1 c&s; Brazil, Minas Gerais, Lagoa da Prata. *Agoniates halecinus*: MZUSP 34327, 4, 1 c&s; Brazil, Amazonas, Rio Marauiá. *Atopomesus pachyodus*: MZUSP 29613, 2 c&s; Brazil, Amazonas, Rio Arirará. *Boulengerella lateristriga*: MZUSP 47710, 1 c&s; Brazil, Amazonas, Rio Urubaxi em Praia de Alagadiço, Rio Negro basin. *Brachychalcinus copei*: MZUSP 38563, 2 c&s; Brazil, Mato Grosso, Rio Branco, tributary of Rio Guaporé. *Brittanichthys axelrodi*: UFRGS 11561, 144, 4 c&s; Brazil, Amazonas, Barcelos, Igarapé Bui-Bui. *Brycon gouldingi*: MZUSP 18069, 1 c&s; Brazil, Pará, Mocajuba, Igarapé Oxipucu, Rio Tocantins basin. *Brycon polylepis*: MZUSP 18142, 1 c&s; Brazil, Pará, Jatobal, Lagoa at Jatobal. *Bryconamericus stramineus*: CI-FML 7160 (ex-MZUEL 4846), 10, 2 c&s; Brazil, Paraná, Maringá, Rio Paraná, arquipélago de Ilha Grande. *Bryconella pallidifrons*: ZUEC 7800, 29, 1 c&s; Perú, Loreto, Iquitos, Quebrada Corrientillo, Río Nanay basin. *Charax leticiae*: CI-FML 7159, 2, 1 c&s; Argentina, Corrientes, San Cayetano, Río Riachuelo. *Ectrepopterus uruguayensis*: UFRGS 8578, 27, 4 c&s; Uruguay, Salto, Arroyo Tala, at ruta 31, between Salto and Artigas, tributary of Río Uruguay. *Erythrocharax altipinnis*: all from Brazil, Pará, rio Curuá. MZUSP 111000, 1, holotype; MZUSP 110999, 6, paratypes; MZUSP 119081, 2, 1 c&s. *Hemigrammus aguaruna*: MUSM 42326, 6, paratypes, all c&s, Peru, Loreto, Río Marañon basin. *Hemigrammus haraldi* ZUEC 10589, 7, 4 c&s; Brazil, Amazonas, Manaquiri, Lago Janauacá. *Hemigrammus* aff*. lunatus*, MUSM 3927, 158, 6 c&s, Peru, Madre de Díos, Rio Tambopata basin. *Hemigrammus marginatus*: CI-FML 7161 (ex-CPUFMT 50), 2, 1 c&s; Brazil, Minas Gerais, Riachinho, Lagoa marginal do Rio Urucuia, Rio S[ã](http://www.booking.com/hotel/br/pousada-sao-francisco-paraty.es.html?aid=354415)o Francisco basin. *Hemigrammus pulcher*: MUSM 34224, 10 from 21; Perú, Loreto, Río Nanay. MUSM 14796, 8, all c&s; Perú, Loreto. ZUEC 8671, 3; Perú, Loreto, Río Itaya. *Hemigrammus rodwayi*: UFRGS 12082, 238, 2 c&s; Brazil, Goiás, Piranhas, Riacho Buritizal, tributary of Rio Piranhas. *Hyphessobrycon compressus*: UFRGS 9683 (ex-UMMZ 143343), 50, 12 c&s; Guatemala, Izabal, Río Ixlu, boundary with Honduras. *Hyphessobrycon erythrostigma*: UFRGS 14510, 10, 2 c&s; aquarium specimens. *Hyphessobrycon loweae*: CI-FML 7162 (ex-ZUEC 12774), 10, 2 c&s; Brazil, Mato Grosso, Paranatinga, Rio Suspiro, tributary of Rio das Mortes. *Hyphessobrycon moniliger*: MZUSP 89117, 863, 5 c&s; Brazil, Goiás, Aruanã, lagoa between Aruanã and Cocalinho municipalities. *Hyphessobrycon montagi*: MPEG 10338, 136, 10 c&s; Brazil, Pará, Juruti, Igarapé Socó Barroso, tributary of Rio Arapiuns. *Hyphessobrycon vanzolinii*: MZUSP 18272, 17, 1 c&s, paratypes; Brazil, Pará, Itaituba, Rio Tapajós, Cachoeira Lombo de Anta, near São Luis. *Moenkhausia ceros*: CI-FML 7148 (ex-ZUEC 8912), 20, 2 c&s; Brazil, Pará, Santarém, Igarapé Capixauã, Vista Alegre. *Moenkhausia* cf. *cotinho*: MZUSP 62137, 1 c&s; Brazil, Amazonas, Santa Isabel do Rio Negro, Rio Negro. *Moenkhausia heikoi*: MZUSP 82460, 3, 1 c&s, paratypes of *Astyanax dnophos*; Brazil, Pará, Rio Xingu, Belo Monte. *Moenkhausia jamesi*: MZUSP 17352, 2 c&s; Brazil, Amazonas, Coari, Rio Solimões, Ilha Sorubim. *Moenkhausia lata*: MZUSP 34661, 2 c&s; Brazil, Amazonas, Anavilhanas, Rio Negro. *Myxiops aphos*: ZUEC 7798, 6, 1 c&s; Brazil, Bahia, Lençóis, Rio Lençóis, at Poço Halley, Rio Paraguaçu basin. *Orthospinus franciscensis*: MZUSP 19664, 16, 1 c&s; Brazil, Minas Gerais, Três Marias reservoir, Rio São Francisco. *Petitella georgiae*: CI-FML 7163 (ex-ZUEC 8314), 20, 2 c&s; Brazil, Amazonas, Iranduba, Lago Janauari, in front of Olaria. *Salminus hilarii:* MZUSP 40113, 8, 2 c&s; Brazil, São Paulo, Pereira Barreto, Rio Tietê. *Tetragonopterus chalceus*: MZUSP 36814, 1 c&s; Brazil, Pará, Altamira, Rio Xingu, Cachoeira do Espelho.
