# Supplementary material for: Phycocharax rasbora, a new genus and species of Brazilian tetra (Characiformes: Characidae) from Serra do Cachimbo, rio Tapajós basin
Source: PLoS One. 2017 Feb 15;12(2):e0170648. doi: 10.1371/journal.pone.0170648 (PMC5310855; doi:10.1371/journal.pone.0170648)
Supplement: S2 Appendix — (DOCX) [file pone.0170648.s004.docx]

S2 Appendix. List of morphological characters used in the phylogenetic analysis. Abbreviations in the character descriptions are references to the following papers: VH [1]; MM [2,3,4]; MW [5]; MT [6]; FE [7]. The number following those abbreviations refers to the number of that character in the cited paper. Cases in which a character definition was modified from its original description are denoted by a “m” after the number, and characters whose states are herein inverted are indicated by an “i”. Characters are reordered from Mirande [2,8] to accommodate the new ones following an anatomical order.

1. Anterior end of mesethmoid: (0) trifurcate, with processes inserted into depressions on premaxillae; (1) not trifurcate, with a triangular anterior spine and articular processes reduced or absent. (MM25).

2. Ventral projection of mesethmoid spine, forming a keel between premaxillae: (0) absent; (1) present. (MM26).

3. Form of mesethmoid spine: (0) long, extending between premaxillae; (1) relatively short, with premaxillae articulating with each other anterior to mesethmoid. (MM27).

4. Posterior portion of mesethmoid spine: (0) relatively slender; (1) as broad as lateral wings of mesethmoid. (MM28).

5. Lateral wings of mesethmoid: (0) present; (1) absent. (MM29).

6. Ventral diverging lamellae of mesethmoid: (0) absent; (1) present. (MM30).

7. Anterior convergence of ventral diverging lamellae with nasal septum of mesethmoid: (0) absent, or confluent near anterior end of nasal septum; (1) confluent at posterior end of nasal septum. (MM31).

8. Nasal septum of mesethmoid: (0) single longitudinal lamella; (1) two parallel lamellae apparently formed, in part, by ventral diverging lamellae. (MM32).

9. Nasal: (0) present; (1) absent. (MM33).

10. Bony lamellae bordering sensory canal of nasal: (0) absent or more slender than tubular region; (1) wider than tubular region at some point. (MM34).

11. Dorsolateral processes of vomer: (0) absent; (1) present. (MM54).

12. Posterior extension of ethmoid cartilage: (0) only to middle horizontal length of orbitosphenoid or less; (1) to vertical through region of articulation between orbitosphenoid and pterosphenoid. (MM49).

In this paper, the cartilage situated just dorsal to the anterior region of the parasphenoid is considered as part of the ethmoid cartilage, instead of being named rhinosphenoid cartilage, as in Mirande [2].

13. Form of anterior process of lateral ethmoid: (0) broad in ventral view, contacting posterior region of vomer in its entire length; (1) slender and separated from vomer. (MM14).

14. Lateral opening between ventral diverging lamellae of mesethmoid and anterior process of lateral ethmoid: (0) broad; (1) small, ovate and partially occluded by diverging lamellae of mesethmoid, dorsal projection of vomer, and anterior process of lateral ethmoid. (MM15).

15. Dorsal margin of lateral ethmoids: (0) aligned; (1) situated oblique in dorsal view, converging in an anterior angle. (MM16).

16. Articulation of medial region of lateral ethmoid dorsal margin: (0) mostly with ventral diverging lamellae of mesethmoid; (1) extensive articulation with mesethmoid dorsal lamella. (MM17).

17. Anterior extension of frontal: (0) reaching posterior margin of nasal opening; (1) extending between nasals and reaching middle length of nasal. (MM20).

18. Contact between frontals anterior to frontal fontanel: (0) absent; (1) present. (MM21).

19. Frontal fontanel: (0) present; (1) totally occluded by frontals. (MM22).

20. Relative length of frontal fontanel: (0) up to 2/3 length of parietal fontanel; (1) 3/4 or more of length of parietal fontanel. (MM23).

21. Dilatator fossa on lateral surface of frontal: (0) absent; (1) present. (MM24).

22. Posterior laminar expansion of epiphyseal bar: (0) absent; (1) present. (MM1).

23. Rhinosphenoid: (0) absent; (1) present. (MM47).

24. Dorsal expansion of rhinosphenoid or rhinosphenoid cartilage: (0) absent; (1) present and forming a bony wall between olfactory nerves. (MM48).

25. Ventral border of rhinosphenoid: (0) distinctly separate from parasphenoid; (1) almost contacting parasphenoid. (MM50).

26. Synchondral articulation between lateral ethmoid and anterodorsal border of orbitosphenoid: (0) present; (1) absent, with orbitosphenoid distant from lateral ethmoid. (MM35).

27. Lateral bony coverage of olfactory nerve: (0) absent; (1) by posterior expansion of lateral ethmoid; (2) by an anterior tubular projection of orbitosphenoid; (3) laterally and ventrally by orbitosphenoid and lateral ethmoid, which do not form canal. (MM36).

28. Form of orbitosphenoid: (0) slender, relatively small, and separate from parasphenoid; (1) massive, almost reaching parasphenoid ventrally. (MM37).

29. Distance between posterodorsal margin of ethmoid cartilage and lateral ethmoids: (0) contacting, or almost contacting, lateral ethmoids; (1) distant from lateral ethmoids. (MM38).

30. Opening between orbitosphenoid and pterosphenoid: (0) present, rounded or ovate, usually margined by frontal dorsally; (1) absent. (MM39).

31. Anterior paired projections of parasphenoid: (0) absent; (1) present. (MM40).

32. Large foramen on pterosphenoid: (0) absent; (1) present, well developed. (MM43).

33. Small foramen near posterior margin of pterosphenoid: (0) absent, or not pierced by nerves; (1) present, pierced by a branch of supraorbital nerve. (MM44).

34. Length of sphenotic spine: (0) not reaching to anterodorsal angle of hyomandibula; (1) reaching to horizontal through dorsal margin of hyomandibula. (MM10).

35. Form of distal tip of sphenotic spine: (0) slender or somewhat expanded; (1) notched, limiting *adductor operculi* anterior and dorsally. (MM366).

36. Position of sphenotic spine relative to hyomandibula: (0) rather aligned with anterior margin of hyomandibula; (1) displaced anteriorly relative to anterior margin of hyomandibula. (MM11).

37. Position of sphenotic spine relative to the orbit: (0) bordering orbit, aligned with anterior margin of infraorbitals; (1) distinctly posterior to orbital margin. (MM12).

38. Temporal fossa: (0) well developed; (1) absent or much reduced. (MM13).

39. Dorsal process of pterotic where tendon from epaxial musculature attach: (0) absent; (1) present, projecting dorsally from tube for semicircular canal. (MM45).

40. Relative length of pterotic spine: (0) projected more posteriorly than hyomandibular ligament; (1) restricted to attachment region of hyomandibular ligament. (MM46).

41. Trigemino-facialis foramen: (0) broad, largely limited by sphenotic dorsally; (1) narrow, as a cleft with sphenotic almost excluded from margin. (MM42).

42. Parietal fontanel in adult specimens: (0) present; (1) absent. (MM41).

43. Position of anterior margin of supraoccipital: (0) behind vertical through posterior orbital margin; (1) anterior to vertical through posterior orbital margin. (MM51).

44. Posterior extension of supraoccipital spine: (0) dorsal of entire neural complex of Weberian apparatus; (1) dorsal of one half or less of extent of neural complex. (MM52).

45. Length of supraoccipital spine: (0) reaching at least to middle length of neural complex; (1) reaching only to anterior limit of neural complex. (MM53).

46. Epioccipital bridge over posttemporal fossa: (0) absent; (1) present. (MM4).

47. Anterior articulation of epioccipital bridge: (0) with both parietal and prootic; (1) only with parietal. (MM6).

48. Posteriorly-oriented epioccipital spine: (0) present; (1) absent. (MM7).

49. Ventromedial opening of posttemporal fossa: (0) absent; (1) present. (MM8).

50. Position of ventromedial opening of posttemporal fossa: (0) between epioccipital and exoccipital; (1) bordered entirely by epioccipital. (MM9).

51. Ventral projection of lagenar capsule: (0) not reaching to plane through parasphenoid dorsal margin; (1) ventral to plane through parasphenoid dorsal margin. (MM3).

52. Position of exoccipital: (0) on middle ventral surface of exoccipital; (1) on articulation between pterotic and exoccipital. (MM18).

53. Ventral longitudinal lamellae of basioccipital: (0) falling short of posterior border of basioccipital; (1) reaching posterior border of cranium. (MM2).

54. Supraorbital: (0) present; (1) absent. (MM70).

55. Contact between supraorbital and sixth infraorbital: (0) absent; (1) present. (MM71).

56. Antorbital: (0) present; (1) absent or fused with first infraorbital. (MM55).

57. Position of antorbital relative to lateral ethmoid in lateral view: (0) antorbital entirely anterior to lateral ethmoid; (1) antorbital overlapping lateral ethmoid. (MM56).

58. Relative position of anterior margin of antorbital and first infraorbital: (0) antorbital either aligned with or anterior to first infraorbital; (1) anterior margin of antorbital posterior to first infraorbital. (MM57).

59. Bony lamellae bordering laterosensory canal of first infraorbital: (0) present; (1) absent. (MM58).

60. Extent of expansion of first infraorbital lateral to maxilla: (0) covering less than one half length of maxilla; (1) covering most of maxilla. (MM59).

61. Lateral overlap of first infraorbital by anterior margin of second infraorbital: (0) absent; (1) present. (MM60).

62. Overlap of maxilla by second infraorbital: (0) absent; (1) present. (MM61).

63. Articulation between second and third infraorbitals: (0) vertical; (1) anteroventrally angled; (2) posteroventrally angled. (MM62).

64. Expansion of anterior region of third infraorbital: (0) not much expanded relative to posterior region of second infraorbital; (1) abruptly expanded relative to posterior region of second infraorbital. (MM63).

65. Ventral extent of third infraorbital: (0) reaching horizontal arm of preopercle; (1) not reaching horizontal arm of preopercle, at least anteriorly. (MM64).

66. Posterior extent of third infraorbital: (0) covering angle of preopercle; (1) relatively reduced, angle of preopercle covered partially by fourth infraorbital. (MM65).

67. Fourth infraorbital: (0) present; (1) absent. (MM66m).

68. Form of fourth infraorbital: (0) reaching posterior margin of third and fifth infraorbitals; (1) absent or excluded from posterior margin of infraorbital series. (VH31; MM66m; MM385).

69. Form of fourth infraorbital: (0) approximately square or more developed longitudinally than dorsoventrally; (1) longer dorsoventrally than longitudinally. (MM67).

70. Posterior dorsoventral expansion of fourth infraorbital: (0) absent: (0) present. (MM68).

71. Lateral coverage of dilatator fossa by infraorbital series: (0) almost complete, at least in its ventral border; (1) leaving a conspicuous naked area in anterior region of dilatator fossa. (MM69).

72. Laterosensory canal in antorbital: (0) absent; (1) present. (MM72).

73. Laterosensory canal of first infraorbital: (0) projects dorsally from main body of first infraorbital; (1) absent or does not projects dorsally. (MM73).

74. Branching of laterosensory canals of fourth or fifth infraorbitals: (0) absent; (1) present. (MM74).

75. Direction of posterior branch of laterosensory canal of fourth or fifth infraorbitals: (0) to a pore on preopercle near hyomandibular condyle; (1) to a pore conspicuously ventral to hyomandibular condyle. (MM75).

76. Laterosensory canal of sixth infraorbital: (0) not branched; (1) branched. (MM76).

77. Position of opening on neurocranium communicating with laterosensory canal of sixth infraorbital: (0) between frontal and pterotic; (1) in frontal. (MM77).

78. Position of opening on neurocranium communicating with sixth infraorbital laterosensory canal: (0) lateral to or slightly anterior to vertical semicircular canal; (1) distinctly anterior to vertical semicircular canal. (MM78).

79. Length of laterosensory canal of dentary: (0) piercing almost entire length of dentary; (1) reduced or absent. (MM79).

80. Pores of laterosensory canal of lower jaw: (0) six or less; (1) seven or more. (MM80).

81. Lateral surface of vertical canal of preopercle: (0) canal uncovered and situated posteriorly to musculature and infraorbitals; (1) covered by musculature and/or infraorbitals. (MM81).

82. Dorsal end of laterosensory canal of preopercle and suprapreopercle: (0) not overlapping anterodorsal process of opercle; (1) overlapping anterodorsal process of opercle. (MM82).

83. Anterior region of laterosensory canal of frontal: (0) contained completely on frontal; (1) opens into a chamber limited dorsally by frontal and ventrally by lateral ethmoid. (MM83).

84. Epiphyseal branch of supraorbital canal: (0) present; (1) absent. (MM84).

85. Epiphyseal branch of corresponding supraorbital canals: (0) both aligned with epiphyseal bar; (1) oriented obliquely, opening posteriorly to epiphyseal bar. (MM85).

86. Opening of epiphyseal laterosensory canals: (0) along margin of cranial fontanel; (1) canals continue dorsomedially in soft tissue, opening over of just lateral to cranial fontanel. (MM86).

87. Laterosensory canal on sphenotic: (0) absent; (1) present. (MM87).

88. Posterior branch of posttemporal laterosensory canal: (0) present; (1) absent. (MM88).

89. Form of lateral line: (0) approximately straight; (1) curved ventrally in abdominal region. (MM89).

90. Degree of ventral curvature of lateral line: (0) straight or only slightly curved, with posterior portion aligned with medial caudal-fin rays; (1) distinctly curved and ventrally situated, with posterior portion lying within ventral half of caudal peduncle and aligned with lower lobe of caudal fin. (MM90).

91. Lateral line: (0) complete; (1) interrupted. (MM91).

92. Isolated pored scale in caudal peduncle: (0) absent; (1) present. (new).
Among the species with an incomplete lateral line, there is a group in which the posteriormost scale of the lateral series is, however, pored as noted by Tagliacollo et al. [9] in *Aphyocharax* Günther and *Prionobrama* Fowler (state 1). That scale is usually not pored in the species with an interrupted lateral line (state 0). This character is coded as inapplicable to species with a complete lateral line. The coding of *Paragoniates alburnus* Steindachner and *Phenagoniates macrolepis* (Meek & Hildebrand) for this character follows Tagliacollo et al. [9].

93. Canal of lateral line on caudal-fin membrane: (0) absent; (1) present. (MM92).

94. Length of caudal-fin canal of lateral line: (0) reaching only half of caudal-fin length; (1) almost reaching posterior margin of caudal fin. (MM93).

95. Interdigitations between premaxillae: (0) present; (1) absent. (MM103).

96. Length of ascending process of premaxilla: (0) reaching at least one-third of length of nasal; (1) reaching just anterior end of nasal. (MM104).

97. Alignment of ascending process of premaxilla: (0) aligned with medial margin of nasal; (1) medially shifted and separated from nasal. (MM105).

98. Form of posterolateral portion of premaxilla: (0) with notch; (1) with pedicle expanded laterally to maxilla. (MM106).

99. Anterior end of ascending process of maxilla: (0) with conspicuous notch; (1) pointed or rounded. (MM94).

100. Anterior process of maxilla: (0) slender relative to posterior region; (1) as thick as posterior region. (VH5; MM371).

101. Ligament from distal tip of ascending maxillary process to premaxilla: (0) absent; (1) present. (VH11; MM377).

102. Ligament from middle length of ascending maxillary process to premaxilla: (0) absent; (1) present. (VH12; MM378).

103. Ligament from maxilla to distal tip of premaxillary alveolar arm: (0) weak, premaxilla moderately bifurcated in its attachment point; (1) strong, premaxilla deeply bifurcated in its attachment point. (VH10; MM376).

104. Flexion on maxilla posterior to alveolar premaxillary arm: (0) absent or not pronounced, maxilla rather straight; (1) pronounced. (VH6; MM372).

105. Ventral margin of toothed region of maxilla; (1) approximately straight; (1) strongly concave. (MM95).

106. Margins of toothed region of maxilla: (0) roughly parallel; (1) dorsally divergent. (MM96).

107. Expansion of lamellar portion of maxilla just posterior to toothed region: (0) absent or not pronounced; (1) very pronounced. (MM97).

108. Tubules for passage of blood vessels on lamellar portion of maxilla: (0) a single tubule, parallel to dorsal margin of maxilla; (1) tubule with anterior branch running parallel to anterior margin of maxilla and reaching one third of its length; (2) anastomosed tubules. (MM98).

109. Posterior extent of maxilla: (0) not reaching second infraorbital; (1) reaching second infraorbital. (MM99).

110. Length of maxilla relative to dentary: (0) maxilla reaching posterior end of Meckelian cartilage; (1) maxilla not reaching posterior end of Meckelian cartilage. (MM100).

111. Ontogenetic lengthening of maxilla: (0) absent; (1) present. (MM101).

112. Form and attachment of primordial ligament: (0) flat and attached to ascending process of maxilla; (1) rotund and attached to distal half of maxillary lamella. (VH8; MM373).

113. Form and attachment of primordial ligament: (0) attached to medial portion of maxilla or dorsal to it; (1) attached near distal tip of maxilla. (VH8; MM374).

114. Dorsal projection of maxilla: (0) not overlaps second infraorbital; (1) overlaps second infraorbital. (MM102).

115. Interdigitations between dentaries: (0) absent; (1) present. (MM111).

116. Form of interdigitations between dentaries: (0) simple bony lamellae; (1) undulate lamellae. (MM112).

117. Form and dentition of anterior region of dentary: (0) toothed and not depressed anteriorly; (1) edentulous and much depressed anteriorly. (MM113).

118. Medial anteroventral notch of dentary: (0) absent; (1) present. (MM114).

119. Medial process of dentary bordering Meckelian cartilage dorsally and medially: (0) absent; (1) present, with mandibular accessory tendon inserted on it. (MM115).

120. Bony lamella covering dentary foramen laterally: (0) absent; (1) present. (MM116).

121. Longitudinal ridge covering laterosensory pores of dentary: (0) absent; (1) present. (MM117).

122. Expansion of dentary lateral to anguloarticular: (0) closer to Meckelian cartilage than to articular socket; (1) closer to articular socket than to Meckelian cartilage. (VH19; MM381).

123. Position of coronomeckelian: (0) situated mainly lateral to Meckelian cartilage; (1) situated mainly dorsal to Meckelian cartilage. (MM110).

124. Lateral ridge of anguloarticular: (0) absent; (1) present. (MM107).

125. Horizontal process of anguloarticular: (0) laterally covered by dentary only anteriorly; (1) broadly covered by dentary, which reaches posterior border of Meckelian cartilage. (MM108).

126. Ventral margin of horizontal process of anguloarticular: (0) posteroventrally angled relative to laterosensory canal of dentary from medial view; (1) perpendicular to laterosensory canal of dentary from medial view. (MM109).

127. Form of posterodorsal portion of anguloarticular: (0) vertical; (1) anterodorsally angled. (VH20; MM382).

128. Morphology of premaxillary, maxillary, and dentary teeth: (0) all teeth conical, caniniform or mamilliform; (1) some teeth multicuspidate or molariform. (MM118).

129. Premaxillary, maxillary, and dentary teeth: (0) not pedunculate, or pedunculate only in some of these bones; (1) pedunculate and uniformly shaped. (MM119).

130. Mamilliform teeth outside mouth: (0) absent; (1) present. (MM120).

131. Premaxillary dentition: (0) one or more series of similar teeth; (1) a recognizable triad of larger teeth with rounded base. (VH1; MM368).

132. A pair of large conical teeth in premaxilla: (0) absent; (1) present. (MM121).

133. Number of rows of premaxillary teeth: (0) one; (1) two or three. (MM122).

134. Number of rows of premaxillary teeth: (0) one or two; (1) three. (MM123).

135. Alignment of teeth on outer premaxillary row: (0) aligned; (1) not aligned, with one or two teeth situated anterior to remaining teeth. (MM124).

136. Gap between medial two teeth from outer premaxillary row: (0) distinctly greater than gap between remaining teeth; (1) equal to gap between remaining teeth. (VH2; MM369).

137. Position of lateralmost tooth from outer premaxillary row: (0) anteriorly displaced relative to remaining teeth of that row; (1) aligned with remaining teeth of that row. (VH4; MM370).

138. Cusps of teeth on outer premaxillary row: (0) one to three cusps; (1) five or more cusps. (MM125).

139. Form of teeth on inner premaxillary row: (0) molariform; (1) with aligned in straight series or anteriorly concave pattern of cusps; (2) with anteriorly concave pattern plus anterior cusps. (MM126).

140. Alignment of cusps of medial teeth on inner premaxillary row: (0) forming anteriorly concave semicircle from ventral view; (1) forming shallow arch or aligned in straight series from ventral view. (MM127).

141. Form of teeth of inner premaxillary tooth row: (0) with cusps forming anteriorly concave arch; (1) with cusps aligned in straight series and without anterior concavity. (MM128).

142. Number of teeth on inner premaxillary row: (0) four or fewer; (1) five or more. (MM129).

143. Number of teeth on inner premaxillary row: (0) seven or fewer; (1) eight or more. (MM130).

144. Polymorphism of teeth on inner premaxillary row: (0) absent; (1) present, with two medial teeth somewhat larger and usually separated from remaining ones by a gap. (MM131).

145. Number of replacement tooth rows on premaxilla: (0) one; (1) two or more. (MM132).

146. Fossa for inner row of replacement premaxillary teeth: (0) absent; (1) present. (MM133).

147. Maxillary teeth: (0) absent; (1) present. (MM134).

148. Number of maxillary teeth: (0) only one, or absent; (1) two or more. (MM135).

149. Number of maxillary teeth: (0) up to three; (1) four or more. (MM136).

150. Extent of implantation of teeth along maxilla: (0) not reaching middle of maxillary lamella; (1) extending across almost entire maxillary lamella. (MM137).

151. Number of cusps of anterior maxillary teeth: (0) conical, a single cusp; (1) three or more cusps. (MM138).

152. Number of cusps of anterior maxillary teeth: (0) up to three; (1) five or more cusps. (MM139).

153. Ontogenetic acquisition of conical teeth on maxilla: (0) absent; (1) present. (MM140).

154. Orientation of anterior dentary teeth: (0) oriented dorsally or anterodorsally; (1) oriented anteriorly, almost parallel to main axis of dentary. (MM141).

155. Number of dentary teeth on outer row: (0) eight or fewer; (1) ten or more. (VH17; MM379).

156. Size and number of anterior dentary teeth: (0) four or five relatively broad teeth at front of dentary; (1) eight or more small and slender teeth at front of dentary. (MM142).

157. Form of third dentary tooth: (0) similar to remaining teeth of dentary; (1) strongly decurved posteriorly. (MM367).

158. Number of cusps of anterior dentary teeth: (0) three or fewer; (1) five or more. (VH18; MM380).

159. Inner row of minute dentary teeth: (0) absent or restricted to lateral region of dentary; (1) present from anterior region of dentary. (MM143mi).

Mirande [2,8] considered the symphyseal dentary tooth (see following character) as part of the inner dentary row of teeth. Further observations revealed that the minute teeth situated just posterior to the fossa for the outer dentary teeth have extraosseus development, while the symphyseal tooth develops within the dentary. Therefore, both kinds of teeth are herein treated as different. Most species lack an inner dentary row of teeth, or if present, it is restricted to a few elements just medial to the posteriormost teeth of the outer row (state 0). In some species, the inner row of teeth is present posterior to all the outer row (state 1).

160. Symphyseal dentary tooth: (0) absent; (1) present. (MM144m).

In this paper, as explained in the preceding character, the symphyseal tooth is not considered as homologous to the teeth of the inner row of teeth, with extraosseous development. Thus, this character is coded with state 0 in the species lacking both kinds of teeth, instead of being coded as inapplicable.

161. Articulation between dentary teeth: (0) absent; (1) present with associated processes and fossae. (MM145).

162. Position of anterior teeth of dentary: (0) along margin of dentary; (1) internally situated with dentary forming anterior ridge. (MM146).

163. Abrupt decrease in size of dentary teeth: (0) absent; (1) present. (MM148).

164. Ethmopalatine cartilage: (0) absent or reduced in size; (1) present and conspicuous. (MM171).

165. Relative length of palatine: (0) approximately one-half length of ectopterygoid, or less; (1) distinctly longer than one-half length of ectopterygoid. (MM172).

166. Palatine foramen: (0) absent or reduced in size; (1) present and very conspicuous. (MM173).

167. Form of anterior portion of ectopterygoid: (0) broad and broadly articulating with palatine; (1) slender, articulating only to lateral margin of palatine. (MM157).

168. Dorsal process of ectopterygoid oriented towards lateral ethmoid: (0) absent; (1) present. (MM158).

169. Ectopterygoid teeth row: (0) absent; (1) present. (MM159).

170. Patch of ectopterygoid teeth: (0) absent; (1) present. (MM160).

171. Position of longitudinal cartilage dorsal to ectopterygoid: (0) bordered medially by mesopterygoid; (1) displaced laterally and separated from medial margin of mesopterygoid. (MM161).

172. Contact between ectopterygoid and anterodorsal region of quadrate: (0) present; (1) absent. (MM162).

173. Mesopterygoid teeth: (0) absent; (1) present. (MM165).

174. Posterior development of mesopterygoid: (0) overlapping metapterygoid; (1) separate from metapterygoid. (VH22; MM383).

175. Anterodorsal lobe of metapterygoid oriented towards mesopterygoid: (0) absent or small and dorsally oriented; (1) present, conspicuous and anteriorly oriented. (MM166).

176. Shape of metapterygoid-quadrate fenestra: (0) rounded or ovate, anteriorly limited by anterodorsal region of quadrate; (1) anteriorly collapsed by convergence of metapterygoid and ventral region of quadrate. (MM167).

177. Opening of metapterygoid for pseudobranch artery: (0) absent; (1) present. (MM168m).

178. Foramen in posterior region of metapterygoid: (0) in form of incomplete arch, bordered posteriorly by hyomandibula; (1) present, encircled by metapterygoid or bordered partially by cartilage. (MM168m).

179. Articulation between ventral margin of metapterygoid and posterodorsal margin of quadrate: (0) absent; (1) present. (MM155).

180. Foramen on articular condyle of quadrate: (0) absent; (1) present. (MM149).

181. Form of quadrate: (0) with ventral portion longer than anterodorsal region; (1) with anterodorsal portion equal or longer than ventral region. (MM150).

182. Posterior extent of ventral process of quadrate: (0) reaching vertical through posterior margin of symplectic; (1) falling short of posterior margin of symplectic. (MM151).

183. Longitudinal ridge in quadrate bordering *adductor mandibulae* muscle ventrally and, to some degree, laterally: (0) absent; (1) present. (MM152).

184. Medial dorsal ridge of quadrate: (0) absent; (1) present, covering symplectic from lateral view. (VH25; MM384).

185. Articulation between quadrate and anguloarticular: (0) anterior to or at vertical through lateral ethmoid; (1) posterior to lateral ethmoid. (MM153).

186. Articulation between quadrate and anguloarticular: (0) anterior to or at vertical through middle eye; (1) posterior to middle eye. (MM154).

187. Suprapreopercle: (0) fused to preopercle; (1) autogenous, separated from preopercle. (MM175).

188. Bony lamellae bordering laterosensory canal of suprapreopercle: (0) absent; (1) present. (MM176).

189. Shape of posteroventral corner of preopercle: (0) acute; (1) rounded. (MM174).

190. Anterior extension of interopercle: (0) extending anteriorly beyond terminus of horizontal arm of preopercle; (1) not extending anteriorly beyond terminus of horizontal arm of preopercle. (MM163).

191. Abrupt posterior expansion of interopercle: (0) absent; (1) present. (MM164).

192. Posterior directed radial striae from articular region of opercle: (0) absent; (1) present. (MM169).

193. Length of medial bony ridge of opercle: (0) 60% or greater than opercular length; (1) less than 50% of opercular length. (MM170).

194. Edentulous basihyal lamella: (0) absent; (1) present. (MM189).

195. Anterior development of basihyal: (0) slightly surpassing anterior margin of hypohyals; (1) broadly extending beyond anterior margin of hypohyals. (MM190).

196. Form of anterior expansion of basihyal: (0) slender, with anterior margin less than two-thirds of its length; (1) expanded, with anterior margin with two-thirds or more of its length. (MM191).

197. Anterior projection of anterior ceratohyal articulating laterally with hypohyals: (0) absent or much reduced; (1) present and achieving half length of hypohyals. (MM177).

198. Hyoid artery: (0) completely contained within anterior ceratohyal in passage from posterior ceratohyal to hypohyals; (1) emerging from anterior ceratohyal near its articulation with posterior ceratohyal. (MM178).

199. Ventral margin of anterior ceratohyal: (0) smooth and without notches; (1) with notches for articulation of branchiostegal rays. (MM179).

200. Number of notches along ventral border of anterior ceratohyal: (0) zero to two; (1) three. (MM180).

201. Articulation between anterior and posterior ceratohyals: (0) synchondral, without bony interdigitations; (1) with bony interdigitations. (MM181).

202. Number of branchiostegal rays: (0) three; (1) four or five. (MM212).

203. Number of branchiostegal rays: (0) three or four; (1) five. (MM213).

204. Anterior portions of branchiostegal rays: (0) broad near their articulation with ceratohyals; (1) slender near their articulation with ceratohyals. (MM214).

205. Attachment of first branchiostegal ray: (0) on proximal one-half length of anterior ceratohyal; (1) posterior to one-half length of anterior ceratohyal. (MM215).

206. Distance between attachment site of first and second branchiostegal rays: (0) equal or shorter than distance between second and third rays; (1) longer than distance between second and third rays. (MM216).

207. Number of branchiostegal rays attached to posterior ceratohyal: (0) one; (1) two. (MM217).

208. First basibranchial: (0) absent or much reduced, not articulating anteriorly with basihyal; (1) well developed and articulating anteriorly with basihyal. (MM182).

209. Contact between lamella on anterior portion of first basibranchial with lamella on posterior portion of second basibranchial: (0) absent; (1) present. (MM183).

210. Bony lamellae between second and third basibranchials: (0) absent; (1) present. (MM184).

211. Bony lamella dorsal to fourth basibranchial: (0) present; (1) absent. (MM185).

212. Main portion of fourth basibranchial: (0) completely cartilaginous; (1) ossified. (MM186).

213. Teeth on lamella dorsal to fourth basibranchial: (0) absent; (1) present. (MM187).

214. Teeth on third pharyngobranchial: (0) present; (1) absent. (MM206).

215. Teeth on fourth pharyngobranchial: (0) present; (1) absent. (MM207).

216. Teeth on fifth pharyngobranchial: (0) present; (1) absent. (MM208).

217. Contact between fourth and fifth pharyngobranchial dentigerous plates: (0) absent; (1) present. (MM209).

218. Shape of dentigerous plate of fifth ceratobranchial: (0) rounded, with posterior notch; (1) elongated, without posterior notch. (MM204).

219. Teeth on fifth ceratobranchial: (0) present; (1) absent. (MM205).

220. Rows of gill rakers on first ceratobranchial: (0) one; (1) two. (MM192).

221. Rows of gill rakers on second ceratobranchial: (0) one; (1) two. (MM193).

222. Rows of gill rakers on third and fourth ceratobranchials: (0) one; (1) two. (MM194).

223. Number of gill rakers on first hypobranchial and ceratobranchial: (0) 16 or more; (1) 15 or fewer. (MM195).

224. Number of gill rakers on first hypobranchial and ceratobranchial: (0) 11 or more; (1) ten or fewer. (MM196).

225. Form of first ceratobranchial gill rakers: (0) pointed and not anteroposteriorly compressed; (1) laminar and much compressed perpendicular to ceratobranchial; (2) short, broad and strongly denticulate. (MM197).

226. Form of anterior gill rakers on first ceratobranchial: (0) not fused; (1) with fused bases forming plates extensively articulated with ceratobranchial. (MM198).

227. Lateral base of gill rakers on first ceratobranchial: (0) slender; (1) broad and laminar at least on anteriormost gill rakers. (MM199).

228. Form and degree of ossification of first ceratobranchial gill rakers: (0) laminar and not ossified distally; (1) rather thick and completely ossified distal region. (MM200).

229. Denticles on gill rakers: (0) present; (1) absent. (MM201).

230. Distribution of denticles on gill rakers: (0) restricted to margins, or absent; (1) along entire surface of gill rakers. (MM202).

231. Rows of gill rakers on first epibranchial: (0) one; (1) two. (MM203).

232. Interhyal: (0) present; (1) absent. (MM210).

233. Length of interhyal: (0) shorter than one-third of symplectic length; (1) equal to or longer than one-half of symplectic length. (MM211).

234. Form and articulation of neural pedicle of third vertebra: (0) pedicle well developed and articulating synchondrally with neural complex; (1) pedicle much smaller and without an articular surface with neural complex. (MM218).

235. Development of transverse process of neural arch of third vertebra: (0) not reaching anterior margin of tripus; (1) well developed and extending beyond anterior margin of tripus. (MM219).

236. Ascending process of neural pedicle of third vertebra: (0) absent; (1) present. (MM220).

237. Dorsal development of dorsal process of neural pedicle of third vertebra: (0) not broadly overlapping neural complex; (1) broadly overlapping neural complex. (MM221).

238. Neural arch and vertebral centrum of fourth vertebra: (0) not fused, autogenous fourth neural arch; (1) fused. (MM222).

239. Anteriorly directed spine at base of first rib: (0) absent; (1) present. (MM223).

240. Laminar bony ridge on dorsal margin of abdominal ribs: (0) absent; (1) present. (MM224).

241. Abdominal ribs on anterior caudal vertebrae: (0) absent; (1) present, associated to first and occasionally second caudal vertebrae. (MM225).

242. Relative number of precaudal vertebrae: (0) exceeding caudal vertebrae in two or more elements; (1) equal or less numerous than caudal vertebrae. (MM226).

243. Total number of vertebrae: (0) 33 or fewer; (1) 34 or more. (VH56i; MM391).

244. Total number of vertebrae: (0) 40 or fewer; (1) 41 or more. (MM227).

245. Total number of transitional vertebrae: (0) four or more; (1) three or fewer. (MM228).

246. Transitional vertebrae with haemal canal: (0) present; (1) absent. (MM229).

247. Position of ventral margin of posttemporal: (0) anterior to lateral margin of epioccipital; (1) lateral or posterior to lateral margin of epioccipital. (MM252).

248. Position of ventral end of posttemporal: (0) anterior or lateral to lateral margin of epioccipital; (1) posterior to lateral margin of epioccipital. (MM253).

249. Ventral exit of laterosensory canal of supracleithrum: (0) covered by posterior lamella of supracleithrum and exiting medially; (1) ventral to lamella of supracleithrum and exiting on posterior margin of this bone. (MM254).

250. Fusion between posttemporal and supracleithrum: (0) absent; (1) present. (MM255).

251. Anterior margin of cleithrum: (0) slightly sinuous; (1) with anterior pointed projection. (MM232).

252. Form of posterior margin of cleithrum: (0) convex or slightly sinuous just dorsal to pectoral-fin insertion; (1) with notch just anterior to pectoral-fin insertion. (MM233).

253. Posterior margin of cleithrum: (0) without concavity ventral to first postcleithrum; (1) with concavity ventral to first postcleithrum. (MM234).

254. Posterior margin of cleithrum: (0) with concavity poorly pronounced or lacking; (1) with markedly concave margin, almost forming straight angle. (MM235).

255. Medial laminar expansion at dorsal tip of cleithrum: (0) absent; (1) present. (MM236).

256. Dorsal development of cleithrum: (0) much extended dorsally to mesocoracoid; (1) ending in a position just dorsal of tip of mesocoracoid. (MM237).

257. Development of medial lamella of coracoid: (0) not expanded; (1) expanded as a keel. (MM238).

258. Bony ridge of coracoid between base of mesocoracoid and ventral margin of interosseous space: (0) absent; (1) present. (MM239).

259. Anterior extension of coracoid ventral lamella: (0) reaching cleithrum; (1) not reaching cleithrum. (MM240).

260. Ventral extension of coracoid lamella: (0) reaching ventral margin of cleithrum; (1) falling short of ventral margin of cleithrum. (MM241).

261. Anterior limit of interosseous space: (0) formed by dorsal margins of coracoid medial lamella and dorsal margin of cleithrum; (1) formed by dorsal margin of coracoid medial lamella and an oblique bony ridge located just ventral to dorsal margin of cleithrum. (MM242).

262. Coracoid foramen: (0) absent or reduced to small pore; (1) well developed. (MM243).

263. Articulation between ventral process of mesocoracoid and dorsal margin of scapula: (0) absent or small; (1) present and broad. (MM245).

264. Ventral articulation of mesocoracoid: (0) anteriorly with coracoid and posteriorly with scapula; (1) only with coracoid. (MM246).

265. Process of scapula forming anterior border of scapular foramen: (0) present; (1) absent. (MM244).

266. Cartilage-filled space anterior to scapular foramen: (0) present, wider than anterior process of scapula; (1) much reduced by expansions of coracoid and cleithrum. (VH49; MM387).

267. First postcleithrum: (0) present; (1) absent. (MM247).

268. Second postcleithrum: (0) present; (1) absent. (MM248).

269. Third postcleithrum: (0) present; (1) absent. (MM249).

270. Form of third postcleithrum: (0) slender, without associated lamella; (1) with a posterior lamella. (MM250).

271. Dorsal development of third postcleithrum: (0) projects dorsally to posterior region of scapula; (1) not projects dorsally to posterior region of scapula. (MM251).

272. Margin of first pectoral ray in adult specimens: (0) not serrated; (1) conspicuously serrated. (MM230).

273. Pelvic bone: (0) not bifurcate anteriorly; (1) bifurcate with conspicuous notch. (MM260).

274. Anterior extension of pelvic bone along main axis: (0) not projecting anterior of lateral and medial lamellae; (1) projecting anterior of lateral and medial lamellae of pelvic bone. (MM262).

275. Anterior tip of pelvic bone: (0) rounded and capped by a small cartilage; (1) pointed, lacking associated cartilage, and frequently projecting outside body wall. (MM263).

276. Dorsal longitudinal ridge on medial lamella of pelvic bone: (0) present; (1) absent. (MM264).

277. Articulation between pelvic bones: (0) through ligaments; (1) with bony interdigitations between ischiatic processes. (MM261).

278. First pelvic-fin ray: (0) not branched; (1) branched. (MM256).

279. Relative length of first pelvic-fin ray of adult males: (0) not extending beyond margin of other rays; (1) extending beyond margin of other rays. (MM257).

280. Number of branched pelvic-fin rays: (0) six or less; (1) seven or more. (MM258).

281. Number of branched pelvic-fin rays: (0) seven or less; (1) eight or more. (MM259).

282. Dorsal myorhabdoi: (0) absent; (1) present. (MM273).

283. Position of anteriormost epineurals: (0) lateral to fourth or fifth vertebrae; (1) reaching to cranium. (MM274).

284. Supraneural anterior to neural spine of fourth vertebra: (0) absent or small; (1) present and vertically elongate. (MM279).

285. Number of supraneurals: (0) four or fewer; (1) five or more. (MM280).

286. Number of supraneurals: (0) seven or fewer; (1) eight or more. (MM281).

287. Bony lamellae associated with supraneurals: (0) absent or small; (1) wider than primary axis of supraneurals. (MM282).

288. Position of last supraneural: (0) located two or fewer vertebrae in front of first dorsal pterygiophore; (1) located more than two vertebrae in front of first dorsal pterygiophore. (MM283).

289. Predorsal spine formed by first dorsal pterygiophore: (0) absent; (1) present. (MM275).

290. Number of dorsal pterygiophores: (0) nine or fewer; (1) 10 or more. (MM276).

291. Number of dorsal pterygiophores: (0) 10 or fewer; (1) 11 or more. (MM277).

292. Number of dorsal pterygiophores: (0) 11 or fewer; (1) 12 or more. (MM278).

293. Relative position of dorsal-fin anterior insertion: (0) anterior to or at vertical through pelvic-fin origin; (1) posterior to vertical through pelvic-fin origin. (MM265).

294. Dorsal-fin rays articulating with first dorsal pterygiophore: (0) two; (1) three or four. (MM266).

295. Anteriorly oriented spine formed by first dorsal-fin ray: (0) absent; (1) present. (MM267).

296. Anterior rays of dorsal fin of adult males: (0) not elongate; (1) elongate and reaching posteriorly close to adipose fin. (MM268).

297. Last unbranched dorsal-fin ray of adult males: (0) approximately as long as first branched ray; (1) distinctly longer than first branched ray and in form of filament. (MM269).

298. Number of branched rays on dorsal fin: (0) eight or fewer; (1) nine or more. (MM270).

299. Relative length of anterior dorsal-fin rays: (0) not reaching tip of posterior rays when adpressed; (1) reaching tip of posterior rays when adpressed. (MM271).

300. Number of dorsal-fin rays on last pterygiophore: (0) one; (1) two, adnate. (MM272).

301. Number of anal pterygiophores anterior to first haemal spine: (0) three or fewer; (1) four or more. (MM293).

302. Proximal and medial radials of anal fins: (0) fused on anterior five pterygiophores; (1) fused in most pterygiophores; (2) medial radials absent or completely fused with proximal ones. (MM294).

303. Lateral lamellae on anterior anal pterygiophores: (0) absent; (1) present. (MM295).

304. Anal-fin position: (0) posterior or almost posterior to vertical through last dorsal-fin ray; (1) extended anteriorly ventral to dorsal fin. (MM284).

305. Number of unbranched anal-fin rays: (0) three or fewer; (1) four or more. (MM285).

306. Number of branched anal-fin rays: (0) ten or fewer; (1) 11 or more. (MM286).

307. Number of branched anal-fin rays: (0) 17 or fewer; (1) 18 or more. (MM287).

308. Number of branched anal-fin rays: (0) 24 or fewer; (1) 25 or more. (MM288).

309. Number of branched anal-fin rays: (0) 34 or fewer; (1) 35 or more. (MM289).

310. Form and length of anterior anal-fin rays: (0) similar to posterior rays; (1) longer and more compressed laterally than posterior rays. (MM290).

311. Number of rays on last anal pterygiophore: (0) two; (1) one. (MM291).

312. Number of epurals: (0) one; (1) two or three. (MM296).

313. Number of epurals: (0) one or two; (1) three. (MM297).

314. Uroneurals: (0) absent or just one pair; (1) two pairs. (MM306).

315. Fusion of hypural 2 to compound centrum: (0) absent; (1) present. (MM298).

316. Fusion between hypurals 1 and 2: (0) absent; (1) present. (MM299).

317. Posterior margin of hypural 3: (0) equal to or narrower than posterior margin of hypural 4; (1) deeper than posterior margin of hypural 4. (MM300).

318. Ventral procurrent caudal-fin rays of adult males: (0) slender; (1) laminar. (MM301).

319. Number of ventral procurrent caudal-fin rays: (0) 11 or fewer; (1) 12 or more. (MM302).

320. Ventral procurrent caudal-fin rays of adult males: (0) not projecting through musculature and skin of peduncle; (1) projecting ventrally through peduncle musculature and skin. (MM303).

321. Caudal-fin bony stays: (0) absent; (1) present. (MM304).

322. Anterior ventral procurrent caudal-fin rays: (0) paired, only distally fused; (1) fused in laminar medial bones. (MM305).

323. Bony hooks on fin rays: (0) absent; (1) present in adult males. (MM307).

324. Anal-fin bony hooks in adult males of species bearing hooks on fins: (0) absent; (1) present. (MM308).

325. Pelvic-fin bony hooks in adult males of species bearing hooks on fins: (0) absent; (1) present. (MM309).

326. Pectoral-fin bony hooks in adult males of species bearing hooks on fins: (0) absent; (1) present. (MM310).

327. Dorsal-fin bony hooks in adult males of species bearing hooks on fins: (0) absent; (1) present. (MM311).

328. Caudal-fin bony hooks in adult males of species bearing hooks on fins: (0) absent; (1) present. (MM312).

329. Bony hooks on base of pelvic-fin rays of adult males: (0) absent, or in small number compared to on segmented portion of rays; (1) as numerous as on segmented portion of rays. (MM313).

330. Bony hooks on last pelvic-fin ray of adult males: (0) absent or reduced in number; (1) as numerous as in other rays. (MM314).

331. Bony hooks on first pelvic-fin ray of adult males: (0) absent; (1) present. (MM315).

332. Position of anal-fin bony hooks of adult males: (0) paired and ordered laterally or posterolaterally; (1) medially positioned and oriented posteriorly; (2) asymmetrically disposed and irregularly arranged. (MM316).

333. Number of anal-fin hooks in adult males: (0) three or more on each ray with hooks (1) only one or two hooks on each ray. (new)

334. Scales: (0) cycloid; (1) ctenoid; (2) spinoid. (MM317).

335. Anterior margin of scales: (0) uniformly curved or slightly undulated; (1) with conspicuous undulations. (MM318).

336. *Circuli* on posterior field of scales: (0) present; (1) absent. (MM319).

337. *Radii* on scales: (0) absent or reduced in number; (1) present and numerous on most scales. (MM320).

338. *Radii* oriented towards anterior field of scales: (0) present; (1) absent. (MM321m).

339. Longitudinal groove on anterior field of scales: (0) absent; (1) present, rather sinuous and without defined margins. (MM321m).

340. *Radii* of scales: (0) not converging at focus; (1) converging at focus. (MM322).

341. Semicircular grooves on posterior field of scales: (0) absent; (1) present. (MM323).

342. Scales covering supraoccipital spine: (0) absent; (1) present and completely covering supraoccipital spine. (MM324).

343. Median predorsal scales: (0) covering entire predorsal region; (1) leaving naked area anterior to dorsal fin. (MM325).

344. Ventral serrae: (0) absent; (1) present. (MM326).

345. Number of scales in lateral series: (0) 33 or more; (1) 32 or fewer. (VH51; MM388).

346. Scales covering anal-fin base: (0) one or two rows of scales covering anal-fin base; (1) several rows covering basal third of anal fin. (MM327).

347. Scales covering caudal-fin lobes: (0) covering only their base; (1) covering one-third of their length. (MM328).

348. Hypertrophied ventral caudal-peduncle squamation: (0) absent; (1) present, restricted to scales below lateral line. (MM354).

349. Mandibular accessory tendon: (0) absent; (1) present. (MM329).

350. Longitudinal position of insertion of mandibular accessory tendon: (0) on vertical through Meckelian cartilage posterior half; (1) on vertical through Meckelian cartilage middle or anterior half. (MM330).

351. Longitudinal position of insertion of mandibular accessory tendon: (0) ventral to Meckelian cartilage; (1) anterior to Meckelian cartilage. (MM331m).
Mirande [2,8] analyzed this character including, as a third state, the insertion of the mandibular accessory tendon on a medial process of the dentary present, which is present in the Iguanodectinae, and was described in his character 115 (herein renumbered as 118). The presence of that process and the insertion of the *adductor mandibulae* tendon on it appear to be redundant, and only one of those characters were herein included. The present character is, thus, coded as inapplicable to the Iguanodectinae.

352. Origin of A1 section of *adductor mandibulae*: (0) principally on vertical arm of preopercle; (1) restricted or almost restricted to horizontal arm of preopercle. (MM332).

353. Insertion of facial tendon: (0) on quadrate near its articulation with preopercle; (1) on preopercle posterior to quadrate. (MM333).

354. Insertion of A1 section of *adductor mandibulae*: (0) on maxilla; (1) on lower jaw. (MM334).

355. Contact between dorsal margin of *adductor mandibulae* and ventral margin of *dilatator operculi*: (0) absent; (1) present. (MM335).

356. Anterior extension of *adductor arcus palatini*: (0) covering most of dorsal surface of mesopterygoid; (1) covering only half of dorsal surface of mesopterygoid. (MM336).

357. Posterior region of *levator arcus palatini*: (0) limited laterally by *adductor mandibulae* and medially by *adductor arcus palatini*; (1) limited lateral and medially by A2 and A3 sections of *adductor mandibulae*. (MM337).

358. Origin of *dilatator operculi*: (0) anterior to vertical through posterior margin of eye; (1) completely posterior to vertical through posterior margin of eye. (MM338).

359. Pseudotympanum lateral to anterior four vertebrae: (0) absent or reduced; (1) developed, limited dorsally by epaxial musculature or *obliquus superioris*. (MM339m; MT9m; MT10m; MT11m).

This and the following five characters expand the analyzed information about the different pseudotympani found in the Characiformes, relative to Mirande [2,8], who only considered the presence or absence of a pseudotympanum with the morphology described by Malabarba [10] for the Cheirodontinae. This set of characters reflects almost the same different states as those analyzed by Mattox & Toledo-Piza [6]. However, some differences in the definition of this transformation series relative with that paper are herein implemented. Mattox & Toledo-Piza [6] coded the presence or absence of a pseudotympanum (character 9), and coded details about its position and involved musculature in different characters (10 and 11, respectively), which they considered inapplicable to the species lacking a pseudotympanum. The inclusion of all kinds of pseudotympani in the state “present” of their character 9 involves the *a priori* assumption that all of them are homologous in the sense that can be considered “the same” at some point. Herein is preferred to consider a priori four kinds of pseudotympani as non homologous due to their positions and associated structures. One kind is a pseudotympanum restricted to the region anterior to the first pleural rib and limited dorsally by bounds of epaxial musculature or *obliquus superioris* muscle (treated in this character), a second kind is a pseudotympanum mainly or completely situated between the second and third pleural ribs and filled only with adipose tissue, a third kind is a longitudinal hiatus between the second and fifth pleural ribs, and a four kind is a deep hiatus between the first and second pleural ribs that is filled with sonic muscles. Those pseudotympani, however, could be considered as serial homologous and both the decision by Mattox & Toledo-Piza [6] or the one taken herein are rather subjective. When treated as different characters, as herein, the absence of each kind of pseudotympani is not redundant, as do happens when all of them are considered homologous. This character treats the presence of a well developed pseudotympanum dorsally margined by epaxial musculature or *obliquus superioris* and restricted to the region just anterior to the first pleural rib (state 1). In the alternative state the pseudotympanum is absent in the region just anterior to the first pleural rib, or if present it is dorsally limited by the *lateralis superficialis* and relatively reduced when compared with the pseudotympanum situated between the first and second pleural ribs (state 0).

360. Pseudotympanum between ribs of fifth and sixth vertebrae: (0) absent; (1) present. (MM339m; MT9m; MT10m).

361. Pseudotympanum between ribs of fifth and sixth vertebrae: (0) absent or restricted to region between these ribs; (1) small portion anterior to first rib limited by *lateralis superficialis*. (MM339m; MT9m; MT10m).

These two characters treat, as an additive series of transformation, the absence (state 0 of both) and, when present, the extension of the pseudotympanum situated principally (state 1 of both) or exclusively (states 0 of character 348 and state 1 of character 349, respectively) between the first and second pleural ribs. In the species coded with state 1 in these two characters, the portion just anterior to the first pleural rib is dorsally limited by the *lateralis superficialis*.

362. Anterodorsal limit of pseudotympanum between ribs of fifth and sixth vertebrae: (0) *lateralis superficialis* or bundles of epaxial musculature; (1) *obliquus superioris*. (MM339m; MT11m).

In the species having a pseudotympanum between the first and second pleural ribs, it is usually well developed and its dorsal margin is, at least partially, delimited by the *lateralis superficialis* or, in some measure, by the epaxial musculature and only the posterodorsal angle of the pseudotympanum is limited by the *obliquus superioris* (state 0). In some species, the pseudotympanum is relatively dorsoventrally reduced and its dorsal margin is completely formed by the *obliquus superioris* (state 1). Mattox & Toledo-Piza [6] distinguished in separate states the cases in which the dorsal margin of the pseudotympanum is limited by the *lateralis superficialis* or by bundles of the epaxial musculature. However, as these two muscles are in different layers, being the *lateralis superficialis* much more lateral than the bundles of epaxial musculature, the differences between these two states are not always unambiguous distinguishable. Therefore, it is herein preferred to consider both cases within the same state. This character is coded as inapplicable to the species lacking a pseudotympanum between the first and second pleural ribs.

363. Pseudotympanum filled with sonic muscles: (0) absent or not filled with sonic muscles; (1) present. (MM339m; MT9m; MT10m).

In the species coded with the state 1 of the three preceding characters, the pseudotympanum, when present, is either restricted to the region anterior to first pleural rib or situated mainly or completely between the first and second pleural ribs. In those species, the pseudotympanum is only filled by adipose tissue and the anterior camera of the swim bladder is visible through it (state 0). In the species of *Serrasalmus* Lacepède, as noted by Mattox & Toledo-Piza [6], there is a large hiatus from the first to the third pleural ribs which is filled by sonic muscles (state 1) [11].

364. Pseudotympanum between ribs of sixth and ninth vertebrae: (0) absent; (1) present, as a longitudinal hiatus. (MM339m; MT9m; MT10m).

Most examined species lack any pseudotympanum between the second and fifth pleural ribs (state 0). Only in *Rhaphiodon* Agassiz, as noted by Mattox & Toledo-Piza [6], there is a longitudinal musculature hiatus between these ribs (state 1).

365. Insertion of pterotic aponeurosis: (0) on pterotic spine, semicircular canal, or just dorsal to it; (1) on pterotic or sphenotic, at middle depth of temporal fossa. (MM340m).
This character was modified from Mirande [2,8] due to redundancy between the state 1, as defined in those papers and the character 56, as herein numbered.

366. Humeral spot: (0) absent or vertically-elongate; (1) horizontally-ovate. (MM341).

367. Second humeral spot: (0) absent or diffuse; (1) present as a conspicuous vertical bar. (MM342).

368. Dark conspicuous spot on dorsal fin: (0) absent; (1) present. (MM343).

369. Horizontal line of chromatophores just dorsal to anal-fin base: (0) absent; (1) present. (MM344).

370. Color of caudal-fin lobes: (0) symmetrically colored; (1) ventral lobe orange or reddish and dorsal lobe hyaline; (2) ventral lobe dark brown or black and dorsal lobe hyaline (MM345).

371. Diffuse spots on flanks: (0) absent; (1) present, especially in young specimens. (MM346).

372. Little spot on each scale of flanks: (0) absent; (1) present. (MM347).

373. Dark spot covering the entire depth of caudal-fin base: (0) absent; (1) present. (MM348).

374. Form of sperm nucleus: (0) spherical or ovoid; (1) much elongated. (MM359; FE16m).

375. Papillae on tongue: (0) not aligned; (1) forming longitudinal rows anteriorly. (MM357).

376. Ventral union of gill membranes: (0) joined anteriorly, but not covering isthmus; (1) joined along length of isthmus but not attached to it; (2) joined to each other and with isthmus. (MM349).

377. Sclerotic bones: (0) single anteroventrally open bone; (1) two bones separated by cartilages. (MM350).

378. Nostrils: (0) rounded and divided only by skin fold; (1) nostrils distinctly separate. (MM351).

379. Fused gill filaments associated with gland cells on males: (0) absent; (1) present. (MM352).

380. Adipose fin: (0) present; (1) absent. (MM356).

381. Insemination: (0) absent; (1) present. (MM358).

382. Sperm storage area on testes: (0) absent or small; (1) present, as broad as spermatogenic area. (MM360).

383. Number of 2n chromosomes: (0) 36 to 42; (1) 46 or more. (MM361).

384. Number of 2n chromosomes: (0) 48 or fewer; (1) 50 or more. (MM362).

385. Number of 2n chromosomes: (0) 50 or fewer; (1) 52 or more. (MM363).

386. Number of 2n chromosomes: (0) 52 or fewer; (1) 54 or more. (MM364).

387. Number of 2n chromosomes: (0) 54 or fewer; (1) 58 or more. (MM365).

**References**

1. Vari RP, Harold AS. Phylogenetic study of the Neotropical fish genera *Creagrutus* Günther and *Piabina* Reinhardt (Teleostei: Characiformes: Characidae), with a revision of the cis-Andean species. Smithson Contrib Zool. 2001: 613: 1‒239.

2. Mirande, JM. Phylogeny of the family Characidae (Teleostei: Characiformes): from characters to taxonomy. Neotrop Ichthyol. 2010; 8 (3): 385‒568.

3. Mirande JM, Jerep FC, Vanegas-Ríos JA. Phylogenetic relationships of the enigmatic *Carlastyanax aurocaudatus* (Eigenmann) with remarks on the phylogeny of the Stevardiinae (Teleostei: Characidae). Neotrop Ichthyol. 2013; 11(4): 747‒766.

4. Mirande JM, Aguilera G, Azpelicueta MM. A threatened new species of *Oligosarcus* and its phylogenetic relationships, with comments on *Astyanacinus* (Teleostei: Characidae). Zootaxa. 2011; 2994: 1‒20.

5. Menezes NA, Weitzman SH. Systematics of the Neotropical fish subfamily Glandulocaudinae (Teleostei: Characiformes: Characidae). Neotrop Ichthyol. 2009; 7(3): 295‒370.

6. Mattox GMT, Toledo-Piza M. Phylogenetic study of the Characinae

(Teleostei: Characiformes: Characidae). Zool J Linn Soc. 2012; 165: 809‒915.

7. Ferreira KM, Menezes NA, Quagio-Grassiotto I. A new genus and two new species of Stevardiinae (Characiformes: Characidae) with a hypothesis on their relationships based on morphological and histological data. Neotrop Ichthyol. 2011; 9(2): 281‒298.

8. Mirande JM. Weighted parsimony phylogeny of the family Characidae (Teleostei: Characiformes). Cladistics. 2009; 25(6): 574‒613. doi: 10.1111/j.1096-0031.2009.00262.x

9. Tagliacollo VA, Souza-Lima R, Benine RC, Oliveira C. Molecular phylogeny of Aphyocharacinae (Characiformes, Characidae) with morphological diagnoses for the subfamily and recognized genera. Mol Phylogenet Evol. 2012; 64(2): 297‒307.

10. Malabarba LR. Monophyly of the Cheirodontinae, characters and major clades (Ostariophysi: Characidae). In: Malabarba LR, Reis RE, Vari RP, Lucena ZMS, Lucena CAS, editors. Phylogeny and classification of Neotropical fishes. Edipucrs: Porto Alegre; 1998. pp. 193‒233.

11. Ladich F, Bass AH. Sonic motor pathways in piranhas with a reassessment of phylogenetic patterns of sonic mechanisms among teleosts. Brain Behav Evol. 2005; 66: 167‒176.
